# Structural Relationships in the Lysozyme Superfamily: Significant Evidence for Glycoside Hydrolase Signature Motifs

**DOI:** 10.1371/journal.pone.0015388

**Published:** 2010-11-09

**Authors:** Alexandre Wohlkönig, Joëlle Huet, Yvan Looze, René Wintjens

**Affiliations:** 1 Structural Biology Brussels and Molecular and Cellular Interactions, VIB, Brussels, Belgium; 2 Laboratoire de Chimie Générale, Institut de Pharmacie, Université Libre de Bruxelles, Brussels, Belgium; 3 Interdisciplinary Research Institute, USR 3078 CNRS, Villeneuve d'Ascq, France; Indiana University, United States of America

## Abstract

**Background:**

Chitin is a polysaccharide that forms the hard, outer shell of arthropods and the cell walls of fungi and some algae. Peptidoglycan is a polymer of sugars and amino acids constituting the cell walls of most bacteria. Enzymes that are able to hydrolyze these cell membrane polymers generally play important roles for protecting plants and animals against infection with insects and pathogens. A particular group of such glycoside hydrolase enzymes share some common features in their three-dimensional structure and in their molecular mechanism, forming the lysozyme superfamily.

**Results:**

Besides having a similar fold, all known catalytic domains of glycoside hydrolase proteins of lysozyme superfamily (families and subfamilies GH19, GH22, GH23, GH24 and GH46) share in common two structural elements: the central helix of the all-α domain, which invariably contains the catalytic glutamate residue acting as general-acid catalyst, and a β-hairpin pointed towards the substrate binding cleft. The invariant β-hairpin structure is interestingly found to display the highest amino acid conservation in aligned sequences of a given family, thereby allowing to define signature motifs for each GH family. Most of such signature motifs are found to have promising performances for searching sequence databases. Our structural analysis further indicates that the GH motifs participate in enzymatic catalysis essentially by containing the catalytic water positioning residue of inverting mechanism.

**Conclusions:**

The seven families and subfamilies of the lysozyme superfamily all have in common a β-hairpin structure which displays a family-specific sequence motif. These GH β-hairpin motifs contain potentially important residues for the catalytic activity, thereby suggesting the participation of the GH motif to catalysis and also revealing a common catalytic scheme utilized by enzymes of the lysozyme superfamily.

## Introduction

Due to a worldwide effort of structural genomics projects, the number of known three-dimensional protein structures rapidly increases [1]. It is now even frequent that structures are determined prior to any knowledge of their biological function [2]. The ability to predict details of protein function and their biological role from structure becomes thus of great importance. To date, several methods are available for this purpose [3]–[8]. Many of them are based on the occurrence of particular clusters of residues, in protein sequence or in protein 3D structure that could give a functional role to the unknown protein [9]–[14]. Such clusters can be also called patterns, motifs, signatures or fingerprints, and were accumulated from various protein families in freely accessible databases, such as PROSITE [15], PRINTS [16], BLOCKS [17], MSDmotif [18] or FunClust [19]. The signature search is also an effective alternative for the detection of remote protein homologues from low-similarity sequences.

The present work was initiated by our previous observation of a highly conserved sequence motif which characterizes glycoside hydrolase family 19 chitinase [20]. We wondered whether the GH families structurally related to GH19 also possess a similar signature motif. The 5 studied GH families, designated as the lysozyme superfamily [21]–[22], are plant chitinase GH19 family, C-type lysozyme GH22 family, G-type lysozyme GH23 family, V-type lysozyme GH24 and the chitosanase GH46 family (http://www.cazy.org/) [23].

Lysozymes (E.C. 3.2.1.17) and chitinases (E.C. 3.2.1.14) represent an important class of polysaccharide-hydrolyzing enzymes. Chitinase enzymes catalyse the breakdown of chitin, a linear polymer found in insects, crustaceans and fungi cell walls consisting of β-1-4 linked N-acetylglucosamine (GlcNAc), while the lysozymes hydrolyse peptidoglycans present in bacterial cell walls which contain alternating β-1-4 linked residues of GlcNAc and N-acetylmuramic acid [24]. The chemical similitude between the two polysaccharide substrates leads to the fact that some lysozymes can hydrolyse chitin, but less efficiently than their natural substrate and *vice versa* [25]–[27]. Thus, some lysozymes could be considered as good chitinases and reciprocally some chitinases can cleave peptidoglycan, the natural substrate of lysozymes [28]. However there is no obvious amino acid sequence similarity found between these two types of enzymes [22]. On the other hand, a different enzyme, chitosanase (E.C. 3.2.1.132), also hydrolyses polymer of GlcNAc, but with specificity for a partial (over 60%) or full deacetylation of chitin, named chitosan. The differences in substrate specificity of these enzymes, and occasionally in their catalytic mechanism, make them belong to different protein families with different E.C. number [23]. All these proteins could be considered, to a large extent, as chitinolytic enzymes, i.e. enzymes that are able to hydrolyze derivatives of chitin [29].

Chitinolytic enzymes are widely distributed in the tissues and body fluids of animals, plants and microorganisms and also in the soil- and bio-spheres of the earth. Chitinases are key enzymes in plant defence systems against fungal infection [30]–[31]. They are classified on the basis of amino acid sequence in two different GH families, namely GH18 and GH19 [32]. Chitinases of GH18 are encountered in all living organisms whereas those of GH19 are mainly found in plants. Proteins of these two GH families significantly differ both in their three-dimensional structures and in their enzymatic mechanisms [33]–[34].

Lysozymes are widely spread throughout nature. They are used by plants and higher organisms as a first defence mechanism against bacterial invasion [35]. Since its discovery by Fleming in 1922, lysozyme has been extensively studied. It was one of the first proteins to be completely sequenced [36] and one of the first enzymes for which the X-ray structure was determined [37]. Several classes of lysozymes have been identified on the basis of their sequence similarities [35]. The best known ones are of the C-type (chicken-type or GH22), the G-type (goose-type or GH23) and the V-type (viral type or GH24).

Chitosanases are classified in GH46 [38]. Most of these enzymes are found in microorganisms and few are found in virus (http://www.cazy.org/). Although chitinases and chitosanases hydrolyze chemically similar substrates that differ only by an acetyl group, no sequence similarities were found between members of these two families.

Polysaccharide-hydrolyzing enzymes commonly use two catalytic residues, a general-acid (proton donor) and a nucleophile/base residue, and they basically perform their function through two different reaction mechanisms, a single-displacement mechanism with a net inversion of an anomeric carbon configuration (inverting enzymes) and a double-displacement mechanism with a net retention of a substrate configuration (retaining enzymes). Whereas the catalytic general-acid residue is localized in equivalent positions in the lysozyme superfamily, the general-base residues are not well structurally conserved in the five families, and even in an extreme case, such as in GH23 and GH46 families, no residue with general base function has been identified. Finally, with the exceptions of GH22 lysozymes that are retaining enzymes, all the other proteins of lysozyme superfamily are inverting enzymes.

## Results

### Structural Relationships in the Lysozyme Superfamily

**Figure 1:**
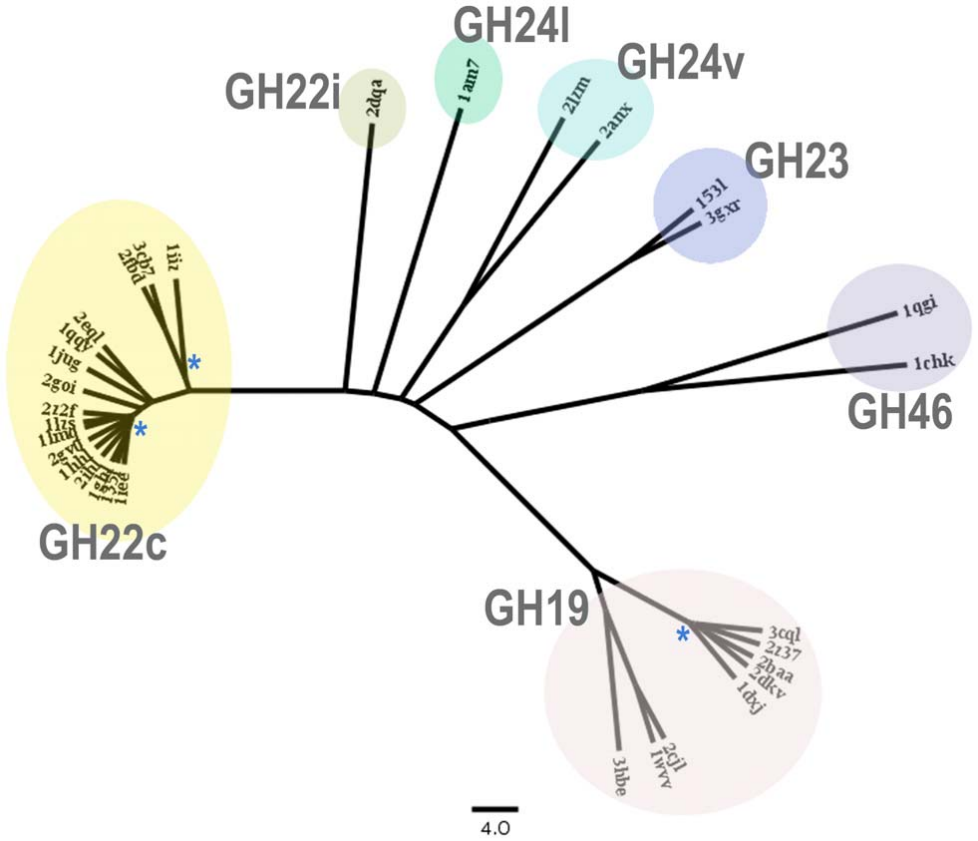
Clustering tree of the 32 structures of lysozyme superfamily. Each enzyme is labelled by its protein code. GH families are indicated. A blue asterisk (*) is set to indicate an unreliable internal node according the jackknife test of Lanyon [70]. The scale below the tree indicated a length of 4.0 in the modified DaliLite scoring scheme (see method).

As protein families of the lysozyme superfamily do not share any sequence similarity, in order to highlight the relationships among these proteins, we compared their structures by computing pairwise structural similarity scores using the DaliLite program [39]. 32 X-ray structures were selected from the protein structure databank (see Methods for the selection criteria). The obtained values of normalized structural similarity score, called DaliLile Z-score, ranged from 1.2 to 47.3, and the superimposition rms values from 0.6 to 4.6 Å. The matrix of Z-score values was transformed in distance metric index and a clustering tree was generated (see Methods). A jackknife procedure has been applied to test the reliability of the resulting tree, which indicated that, except for some internal nodes within the GH19 and GH22c clusters, all of the nodes were stable (Fig. 1).

The obtained tree (Fig. 1) shows the similarities and differences among proteins of the lysozyme superfamilies, but also indicates structural relationships in a given GH family: (i) lysozyme superfamily exhibits a structural continuum with the different GH families roughly structurally equidistant from each other; (ii) according to our structural similarity index, the range of distances between the five GH families was 63–82; (iii) the mean distance between the two more distant GH families, namely GH46 and GH19 families, was 82.4, whereas this distance was 63.4 between GH23 and GH24, the two closest families; (iv) although they are grouped in a same GH family, a large distance was found between the two classes of both GH22 and GH24 families, i.e. the distance between GH22c and GH22i was 46.7 and 57.7 between GH24l and GH24v; (v) the chitinase GH19 family showed two structurally distinct clusters and a mean distance between all members of 23.3 (Table 1). One cluster grouped the plant chitinases and the second one the bacterial chitinases, except the recent structure of Norway spruce chitinase which was curiously grouped with the bacterial chitinases; this could be explained by the fact that the latter chitinase is a class IV chitinase while the other plant chitinase are class I or II; (vi) although the two structures of GH46 chitosanase family are bacterial proteins, the distance between them was high (46.2), in the same order of magnitude as distance between the two classes GH22c and GH22i.

**Table 1:** Data summary of GH motif.

GH family	#struct^a^	<Dij>^b^	Rep struct^c^	#seq^d^	GH motif range^e^	HMM performance^f^
GH19	8	23.32	3cql	998	111–126	0.99/0.10
GH22c	16	13.46	1iee	286	52–64	1.00/0.37
GH22i	1	–	2dqa	39	34–42	1.00/0.02
GH23	2	14.20	153l	103	89–98	0.43/0.18
GH24v	2	36.90	2lzm	190	17–32	0.98/0.50
GH24l	1	–	1am7	175	61–69	0.77/0.05
GH46	2	46.20	1qgi	46	55–71	1.00/0.04

a number of structures analysed in the work. The complete list of structures is given as supplementary materials.

b mean structural distance (see methods) computed on all structure members of the given GH family.

c protein code of the representative structure.

d number of sequences used in the work. Multiple global alignments are given as supplementary materials.

e limits of the GH motif according numbering of the representative structure.

f profile HMM search performance given as TPR/FPR where TPR and FPR are true and false positive rates, respectively.

Note that the topology of the clustering tree was further confirmed using MAMMOTH-mult server [40], another structure-comparison tool, which produced a very similar result.

### Common Structural Features in the Lysozyme Superfamily

**Figure 2:**
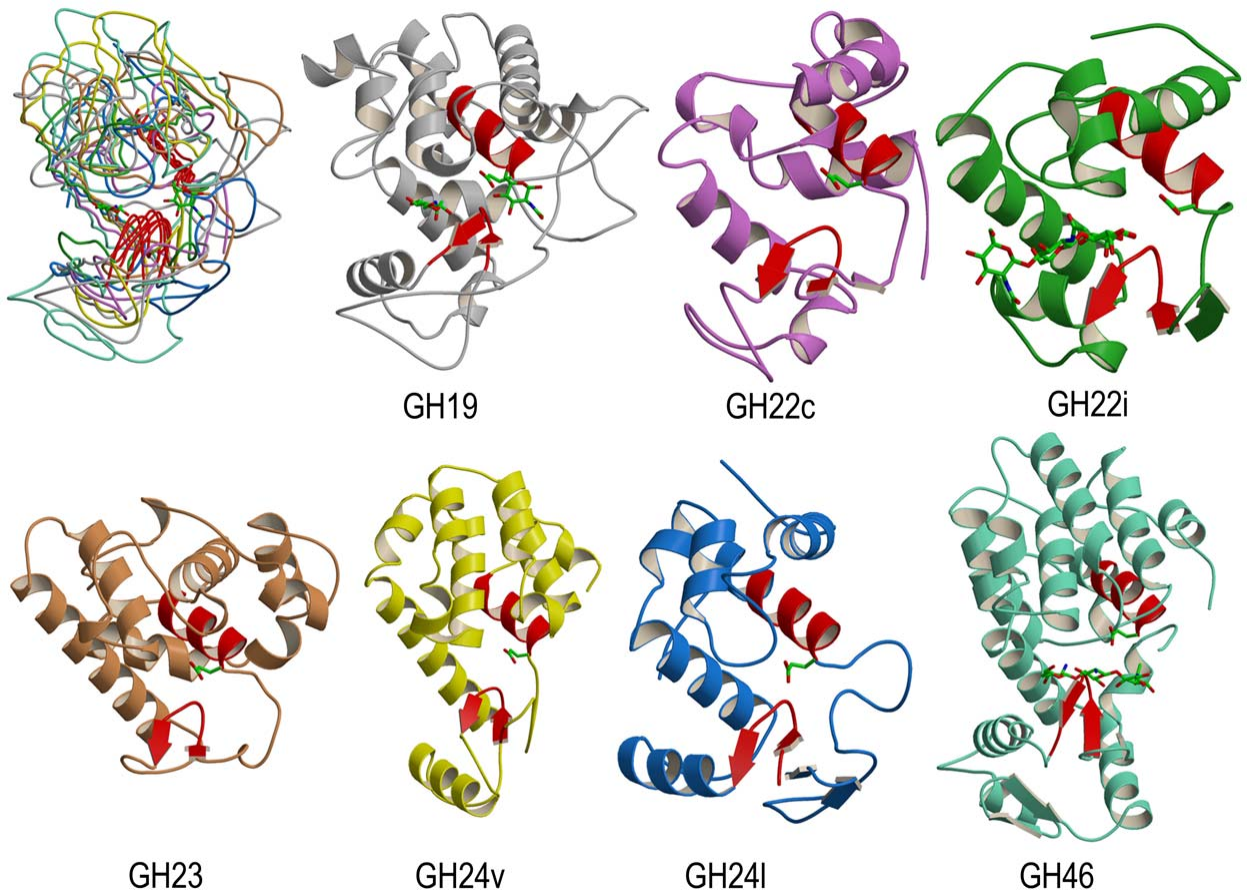
Structural superposition of the seven GH representative structures. Top left picture shows a superimposition of the 7 representative structures. Each representative GH structure is further shown in coloured ribbon. The protein colour scheme is grey for GH19 structure (protein code: 3cql), purple for GH22c (1iee), green for GH22i (2dqa), brown for GH23 (153l), yellow for GH24v (2lzm), blue for GH24l (1am7) and aquamarine for GH46 (1qgi). To locate the substrate binding site, the conserved catalytic glutamic acid (E67, E35, E18, E73, E11, E19 and E37 for GH19, GH22c, GH22i, GH23, GH24v, GH24l and GH46, respectively), as well as the sugar moieties found as ligands in X-ray representative structures of GH19, GH22i and GH46 are depicted in stick representation. The two regions of common structural core are showed in red colour. Limits of these regions in representative structure are 58-67/112-121 for GH19, 26-35/51-60 for GH22c, 9-18/34-43 for GH22i, 64-73/89-98 for GH23, 2-11/24-33 for GH24v, 10-19/62-71 for GH24l and 28-37/59-68 for GH46.

Even though the seven representative structures of the lysozyme superfamily have no sequence similarity and vary considerably in length, pairwise structure superimpositions showed that they share a common fold, consisting of two domains separated by the binding cleft (Fig. 2) [21]–[22]. The large domain is mainly α-helix and the second one essentially contains three antiparallel β-strands which form a β-sheet. By listing the structurally equivalent residue ranges obtained by the superposition programs, two regions can be defined as the common structural core of the lysozyme superfamily (shown in red colour in Fig. 2). The first region is the C-terminal part of the central helix of all-α domain which contains the conserved glutamic acid proposed to act as general-acid catalyst. The second element of the common core is a β-hairpin structure located close to the catalytic site.

### Structural Motif Containing High Sequence Conservation

**Figure 3:**
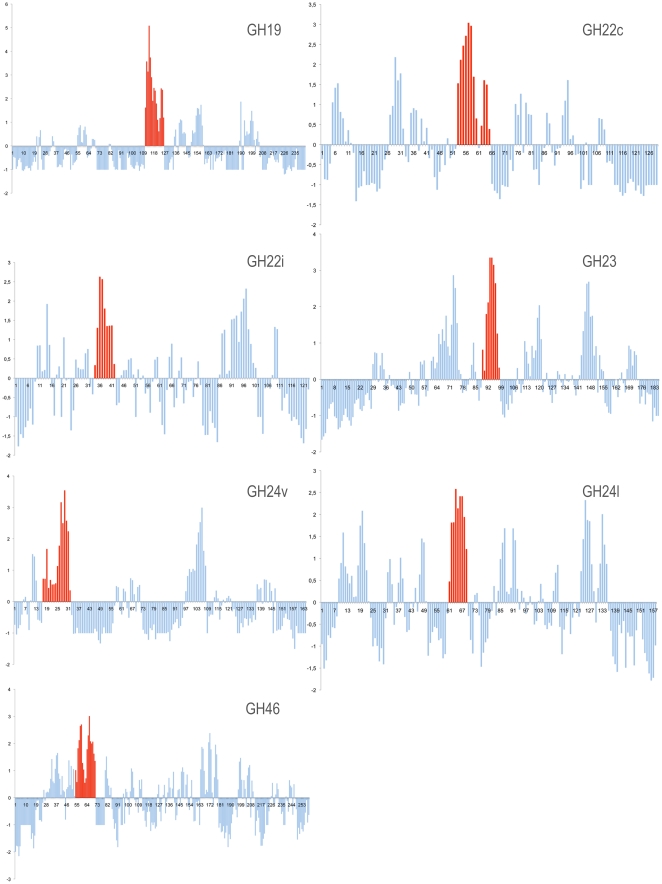
Amino acid conservation profiles in aligned sequences of GH families. AL2CO conservation indices [41] at each position in multiple sequence alignment for each GH families are mapped on the sequence of the corresponding representative protein. The regions displaying the highest conservation indices are showed in red.

For the five studied GH families, here subdivided into seven different sub-families, amino acid sequences were collected and aligned (multiple alignments are given in Supplementary Figures S1–S7). Using AL2CO program [41] on multiple alignments, a conservation profile was then derived for each family (Fig. 3). This program estimates a conservation index at each position in a multiple sequence alignment, based on amino acid frequencies at each position. Positions of functional and/or structural importance generally tend to be more conserved in a given protein family and therefore to have high conservation indices. We observed that, for each GH family, the region with higher sequence conservation was located in the β-hairpin of the common structural core (Fig. 3). This is clearly visible in sequence conservation profiles of GH19, GH22c and GH24v, and though to a lesser extent, similar results were observed in profiles derived with the less populated families such as GH22i, GH23 and GH46.

### GH Sequence Signatures

**Figure 4:**
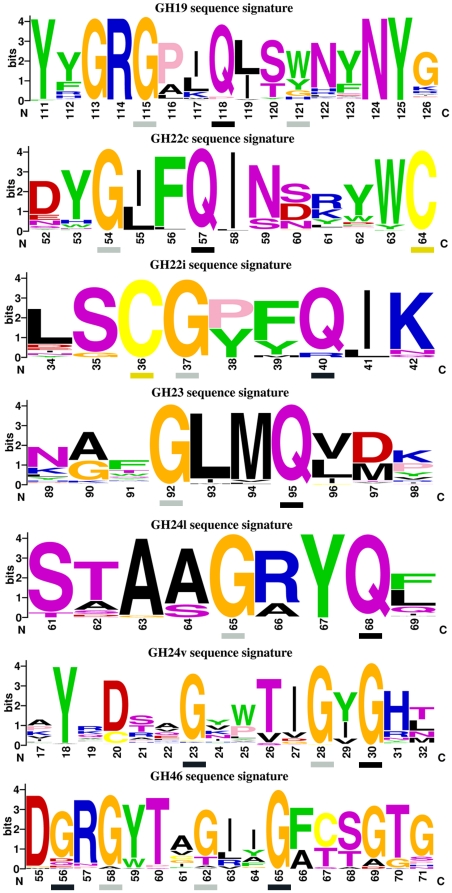
WebLogo sequence signatures for GH motifs. Basic amino acid (K, R, H) are coloured in blue, acidic (D, E) in red, aliphatic (A, V, L, I, M) in black, aromatic (W, F, Y) in green, polar (N, S, T, Q) in purple, glycine in orange, cysteine in yellow and proline in pink. Amino acid sequence numbering is based on the one of the representative structure of each GH family. Residues displaying backbone positive ϕ angle in X-ray structures are indicated by a grey or black box for extended or helical left handed conformations, respectively. Cysteine residues participating in disulfide bond are indicated by yellow boxes.

Using the conservation sequence profiles, a GH sequence signature for each family was defined by the region of strongest sequence conservation and containing the common β-hairpin. To better visualize the high degree of conservation of these regions, a weblogo figure [42] showing coloured amino acid distribution at each position along each GH signature motif was derived (Fig. 4). All obtained weblogos were significantly different between the different GH families. The only common feature was the presence of conserved Gln and/or Gly at particular positions, i.e. Gly113, Gly115 and Gln118 in GH19, Gly54 and Gln57 in GH22c, Gly37 and Gln40 in GH22i, Gly92 and Gln95 in GH23, etc… For a given GH family, the high degree of conservation for a particular residue at specific positions may indicate a requirement for a specific structural conformation or for a functional role. This point will be discussed later.

### Search Performances of GH Sequence Signatures

To benchmark the sensitivity (true positive rate) and selectivity (false positive rate) performance of each GH signature, profile hidden Markov models (profile HMMs) were derived using HMMER3 software [43]. We tested how well each profile HMM could identify the members of its GH family from all sequences in uniprot-trembl databases and how many false recognitions were found. The results showed that GH sequence signatures have high sensitivity except GH23 and GH24l signatures (Table 1). False positive rates were relatively low for most of GH signatures, and signatures having higher false positive scores were due to sequence identification of other related protein families that were not included in the starting sequence data sets, i.e. GH22c signature detected many sequences of α-lactalbumins, GH23 signature identified several lytic murein transglycosylase sequences and GH24v found several members of *E. coli* endolysin protein family. Note also that the obtained false positive rates were probably overestimated as many false positive are sequences of putative uncharacterized proteins, which could indeed be GH enzymes.

### GH Structure Signatures

Besides displaying specific sequence signature, the GH motifs have several structural features in common. All contain at least a β-hairpin structure and are located close the catalytic binding site of the enzymes (Fig. 2). A type I β-turn was systematically found in the β-hairpin, except for GH24v motif. The β-hairpin is classified as type 4∶4 hairpin, a class of hairpins usually found in protein structures to contain a type I β-turn [44]. The first (i) and the last (i+4) position of the β-turn were mostly occupied by glycine and glutamine, respectively (Fig. 4). In 3D structures, these two residues always adopt positive ϕ torsion angle or left-handed conformation, but whereas the first residue (i) is in left-handed extended conformation, the second one (i+4) exhibits left handed helical backbone conformation. This was observed in almost all GH motifs except in GH24v and GH46 motifs (Fig. 4).

### Variations Around a Common Active Site Configuration

GH β-hairpin motifs certainly participate in the mechanisms of action of the glycosidases, not only due to its spatial proximity to active site but probably also due to the presence of catalytically important residues. In particular, two residues, which are almost invariant in all lysozyme superfamily motifs, appear important: the Ser/Thr residue knowing to interact with the catalytic water molecule of inverting mechanism, and a Gln residue whose exact role in catalysis was not studied yet. Active site configurations of the 32 glycosidase structures are summarized in Table 2 by calculating separation distances between key or putative catalytic residues.

**Table 2:** Active site configuration of lysozyme superfamily^a^.

	GH19	GH22c	GH22i	GH23	GH24v	GH24l	GH46
(A) General-acid catalyst residue	Glu67	Glu35	Glu18	Glu73	Glu11	Glu19	Glu37
(B) General-base catalyst residue	Glu89	Asp52	Asp30	Asp97^b^	Asp20	/	Asp55
(C) Catalytic water positioning residue	Ser120	/	Ser35^c^	/	Thr26	Ser61^c^	Thr60
(D) Putative accessory active site residue	Gln118	Gln57	Gln40	Gln95	/	Gln68	/
Mean distance (A)–(B) (Å)	8.88(0.24)	5.81(0.11)	6.26(–)	8.63(0.19)	8.56(1.80)	/	11.51(0.86)
Mean distance (A)–(C) (Å)	7.66(0.20)	/	6.74(–)	/	8.81(1.90)	8.27(–)	10.62(1.38)
Mean distance (B)–(C) (Å)	4.50(0.22)	/	2.67(–)	/	4.79(0.26)	/	4.83(0.07)
Mean distance (A)–(D) (Å)	4.27(0.22)	3.50(0.05)	3.33(–)	3.37(0.13)	/	3.65(–)	/
Mean distance (B)–(D) (Å)	6.33(0.07)	4.77(0.08)	5.66(–)	7.85(0.34)	/	/	/

a distances were calculated as the shortest separation observed in the X-ray structures of the considered GH family between side chain oxygen atoms of residues Asp, Glu, Ser and Thr. For Gln residue, only the atom Cβ is used. Standard deviations are given in parentheses.

b this residue is proposed as the putative general base.

c besides Gln residue (D), these residues were suggested in this work to participate to catalytic process.

Glycosidases of lysozyme superfamily show flexibility in their active site configuration mainly as all putative catalytic residues are not always present (Table 2). Nevertheless, some features are shared. First, distances between carboxyl groups are consistent with the principle that a short separation is observed in retaining glycosidases whereas inverting enzymes have longer distances [45]. The conserved Gln amino acid is found between the two catalytic carbonyl groups. A hydroxyl group is also found between the two carbonyls, but much closer to general-base carbonyl than the general-acid one. Note that Ser61 in GH24l spatially occupies the position of the lacking general-base catalyst residue, suggesting its participation in catalysis.

## Discussion

### Lysozyme Superfamily GH Motifs and Catalytic Function

The seven GH families and sub-families of the lysozyme superfamily share not only a common global fold but also a common β-hairpin structural motif that exhibits the highest amino acid conservation in aligned sequences and that is positioned in spatial structures close to the substrate binding site. Sequence signatures derived from the regions including the common β-hairpin are found to be specific for their corresponding GH sub-family. The different GH signatures show very little resemblance between them, thereby underlining the high sequence plasticity of the common β-hairpin structure between the GH families of the lysozyme superfamily.

Amino acids defining a sequence signature are generally conserved in protein family to fulfil structural and/or functional roles. Here, many residues of GH signature motifs clearly play a structural role while others are key residues for glycoside hydrolase activity (Table 2). In particular, many Gly residues of the GH signatures are conserved due to the intrinsic property of this amino acid to easily accept to adopt positive dihedral ϕ angle, in the same manner as Cys residues implicated in disulfide bond are also highly conserved (Fig. 4).

Previous site-directed mutagenesis and structural studies have emphasized the importance of many residues constituting the here-described GH motifs for the protein function. In GH19 family, Thr/Ser120 (throughout this paper, residues are labelled according to numbering of the representative structure) plays an essential role in the enzymatic mechanism [46], being found in X-ray structures H-bonded with the presumed catalytic water molecule [20], [47]. Gln118 and Asn124 were also described as key residues for protein activity [46], [48]. Tyr123 does not participate directly to catalysis, but is relevant for productive substrate binding [48]–[51]. In fact, all these latter residues were found to participate in substrate binding interactions in GH19 family [20].

The GH22c signature motif contains the second catalytic Asp/Glu52 that takes part in enzymatic reaction by stabilizing the oxocarbonium ion intermediate in the dissociated form [52]. The functional role of Trp/Tyr62 has been extensively studied [53]–[55]. This residue is a major determinant of substrate binding specificity toward a productive binding mode. Ile/Leu55, Gln57 and Leu58 are involved in protein stability [56], [57]. On the contrary, no information is available on the functional role of residues of GH22i signature motif. For the GH23 motif, X-ray structures of G-type lysozyme in complex with GlcNAc molecules showed the participation of Asp97 to position the catalytic water molecule for nucleophilic attack [58], [59]. Note however that this Asp97 is only partially conserved among GH23 sequences and therefore its function as catalytic residue could not be generalized to all members of GH23 family (Fig. 4).

T4 lysozyme, the archetype for viral-type lysozyme GH24v family, has been extensively studied by mutagenesis experiments mainly for investigations of protein folding and stability mechanisms. The most interesting insight in relation with this work is the pivotal role played by Thr26, a key residue of the GH24v motif. The substitution Thr26 → His changes the catalytic properties of the T4 lysozyme from an inverting to a retaining enzyme [60]. The Gln68 of GH24l motif is observed in interaction with substrate [61]. Finally, Thr45 of GH46 motif was found to be essential to perform catalysis [46].

### Lysozyme Superfamily Evolution

It is generally accepted that proteins of lysozyme superfamily have diverged from a hypothetical common ancestor [21], [22], [62]. Even though their amino acid sequences appear to be unrelated, it could be reasonable to argue that overall structural similarities between lysozyme superfamily proteins are a good indication that they have evolved from the same precursor. The high sequence similarity regions that we identified here and that are part of catalytic sites show strong specificities towards their corresponding GH families. The GH signature motifs all are different, having in common only few residues. In the hypothesis of divergent evolution this means that the ancestral fold, including the GH β-hairpin motif, has been conserved across species and during evolution while the complete sequences have diverged.

### Conclusion

The seven families and subfamilies of the lysozyme superfamily have all in common a β-hairpin structure close to their substrate binding cleft. In each considered family, the region containing the β-hairpin structure shows the higher conservation score among aligned protein family sequences. Each β-hairpin motif further displays a family-specific sequence motif. The presence of residues expected to be catalytically important in the β-hairpin motifs suggests the participation of this GH motif to catalysis. Finally, many of the GH motifs contain a glutamine residue in left-handed conformation; its precise role in the protein function has yet to be defined.

## Material and Methods

### Data Collection

Sequences of the five GH families (GH19, GH22, GH23, GH24 and GH46) of lysozyme superfamily were retrieved by blast searches [63] on uniprot-trembl database release 2010_4 [64] using as query sequences a representative sequence for each considered GH family. As the family GH22 contains two distinct types of lysozymes which share no sequence similarity, this family was subdivided into GH22c (for the C-type) and GH22i (for the I-type) lysozymes. For the same reason GH24 was also subdivided in two different subgroups, namely GH24v (viral-type lysozyme) and GH24l (lambda-type lysozyme). Representative sequences for GH19, GH22c, GH22i, GH23, GH24v, GH24l and GH46 family were, respectively, *papaya* endochitinase (swiss-prot(sw):chit_carpa), hen egg-white lysozyme (sw:lysc_chick), *Tapes japonica* lysozyme (UniProt:q8iu26_venph), goose lysozyme (sw:lyg_ansan), phage T4 lysozyme (sw:lys_bpt4), lambda phage lysozyme (sw:lys_lambd) and bacillus chitosanase (sw:chis_bacci). Note also that the GH22-related α-lactalbumins were not included in this study. 998, 286, 39, 104, 191, 176 and 47 sequences were collected for family GH19, GH22c, GH22i, GH23, GH24v, GH24l and GH46, respectively. The X-ray structures of the representative sequences were used throughout this study as representative structures for the corresponding GH family. A multiple alignment was achieved within each GH family. Initial multiple alignments were obtained with clustal program [65] and further manually adjusted with BioEdit program [66].

Structures were also retrieved by blast queries [63] on sequences of Protein Data Bank [67] using the seven representative sequences. Only X-ray structures were considered. However, lysozyme structures were over-represented in the Protein DataBank. For instance, 660 and 556 structures were initially obtained for family GH22c and GH24v, respectively. To avoid redundancy in structure data, pairwise sequence comparisons [68] were performed in each GH family. Structures displaying more than 95% of identity on a sequence alignment length of more than 90% were ruled out. By this procedure, the number of structure of family GH22c and GH24v decreased to 16 and 2, respectively. In total, 32 X-ray structures have been selected for this study; a table containing all the structures is given in supplementary material Table S1. Protein codes of representative structure for family GH19, GH22c, GH22i, GH23, GH24v, GH24l and GH46 were 3cql, 1iee, 2dqa, 153l, 2lzm, 1am7 and 1qgi, respectively.

### Sequence Analysis

Analysis of amino acid conservation in GH family sequence alignments were performed with AL2CO program [41], using as parameters a smooth length of 3 residues, BLOSUM62 as scoring matrix, the independent count for sequence weighting scheme and entropy as conservation calculation method [41]. WebLogo server [42] was used to plot the amino acid distribution at each position of GH motifs, for which the height of each letter is proportional to its relative frequency at that position and the overall height of the stack indicates the sequence conservation at that position.

Sensitivity and specificity of each GH motif was assessed by profile Hidden Markov Model (HMM) search against uniprot-trembl database (release 2010_06) using a profile HMM derived from GH signature motif and HMMER 3.0 package with all default parameters [43]. The HMMER software package is commonly used to search a sequence database for homologues of a protein family of interest. In evaluation of search performance of profiles HMM, true positives were correct identifications of initial GH motif sequences, whereas false positive scores were corrected by removing sequences assigned to belong to the considered GH family according the Cazy database (http://www.cazy.org/).

### Structure Analysis

Structural comparisons between the selected 32 crystal structures were done with DaliLite program [39], resulting in a matrix of similarity Z-scores. DaliLite is a widely used program for pairwise protein structure comparison and for deriving an optimal protein structural alignment. The quality of the structural alignment is assessed by the value of DaliLite Z-score, which is a structural similarity score normalized with respect to protein domain size. As a general rule, a DaliLite Z-score above 20 means the two structures are definitely homologous, between 8 and 20 means the two are probably homologous, and a Z-score below 2 is not significant. In this work, when several chains were present in the X-ray structure, only the first one was used (generally named chain A). The similarity Z-score matrix was modified into distance matrix in order to infer clustering tree using the unweighted pair-group method with arithmetric averages (UPGMA) of PHYLIP package [69]. For structures i and j, the DaliLite Z-score was transformed as follow:



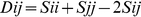
(1)


where *Dij* is the distance between structures i and j, and *Sij* is the DaliLite Z-score computed between structures i and j.

The reliability of the tree was assessed on the basis of the jackknife test of Lanyon so as to identify the unreliable internal nodes [70]. The jackknifing procedure, which is a resampling method like bootstrapping, proceeded in the following way: each structure in the dataset is singled out in turn as an independent test sample, and a tree is derived from all the remaining structures. An internal node was estimated as reliable only if it was found in all possible trees. Trees were drawn using FigTree program [71]. Web services on MAMMOTH-mult server [40] were used to compute multiple structure alignments and additional pairwise structural alignments were made with SoFiSt program [72].

Secondary structure assignments were performed by DSSP program [73], except for GH19 and GH24l where definitions from PDB files were used as DSSP failed to correctly assign β-strands in these structures. All structures were analysed with the Promotif program [44]. All figures of 3D structures were produced using successively MolScript [74] and Raster3D [75] programs.

## Supporting Information

Table S1 List of the 32 X‐ray structures used in this study. (PDF)

Figure S1 Multiple alignment of GH19 motif sequences. (PDF)

Figure S2 Multiple alignment of GH22c motif sequences. (PDF)

Figure S3 Multiple alignment of GH22i motif sequences. (PDF)

Figure S4 Multiple alignment of GH23 motif sequences. (PDF)

Figure S5 Multiple alignment of GH24v motif sequences. (PDF)

Figure S6 Multiple alignment of GH24l motif sequences. (PDF)

Figure S7 Multiple alignment of GH46 motif sequences. (PDF)
