# Supplementary material for: Structural Relationships in the Lysozyme Superfamily: Significant Evidence for Glycoside Hydrolase Signature Motifs
Source: PLoS One. 2010 Nov 9;5(11):e15388. doi: 10.1371/journal.pone.0015388 (PMC2976769; doi:10.1371/journal.pone.0015388)
Supplement: Table S1 — List of the 32 X‐ray structures used in this study. (PDF) [file pone.0015388.s001.pdf]

Table T1. List of the 32 X-ray structures used in this study.

GH family	PDB code	res[a]	source
GH19	3cql	1 .50	*Carica papaya*
	2baa	1.80	*Hordeum vulgare*
	2dkv	2.00	*Oryza sativa*
	1qxj	1.80	*Jack bean*
	1z37	1.53	*Brassica juncea*
	2cjl	1.50	*Streptomyces coelicolor*
	1wvv	2.00	*Streptomyces griseus*
	3hbe	1.55	*Picea abies*
GH22c	1iee	0.94	*Gallus gallus*
	2ihl	1.40	*Coturnix coturnix jap*
	135l	1.30	*Meleagris gallopavo*
	1hhl	1.90	*Numida meleagris*
	1ghl	2.10	*Phasianus colchicus*
	2gv0	1.90	*Pelodiscus sinensis*
	1lmq	1.60	*Oncorhynchus mykiss*
	1lzs	1.60	*Homo sapiens*
	2z2f	1.50	*Bos taurus*
	1qqy	1.85	*Canis familiaris*
	2goi	2.30	*Mus musculus*
	2fbd	1.90	*Musca domestica*
	1jug	1.90	*Tachyglossus aculeatus*
	2eql	2.50	*Equus caballus*
	1iiz	2.40	*Antheraea mylitta*
	3cb7	1.90	*Musca domestica*
GH22i	2dqa	1.60	*Tapes japonica*
GH23	153l	1.60	*Anser anser anser*
	3gxr	1.70	*Gadus morhua*
GH24v	2lzm	1.70	*Enterobacteria phage T4*
	2anx	1.04	*Enterobacteria phage p22*
GH24l	1am7	2.30	*Enterobacteria phage lambda*
GH46	1qgi	1.60	*Bacillus circulans*
	1chk	2.40	*Streptomyces sp. n174*

[a] x-ray solution
