# Supplementary material for: Structural Relationships in the Lysozyme Superfamily: Significant Evidence for Glycoside Hydrolase Signature Motifs
Source: PLoS One. 2010 Nov 9;5(11):e15388. doi: 10.1371/journal.pone.0015388 (PMC2976769; doi:10.1371/journal.pone.0015388)
Supplement: Figure S1 — Multiple alignment of GH19 motif sequences. (PDF) [file pone.0015388.s002.pdf]

Figure S1. Multiple alignment of GH19 motif sequences.

```
                                     10
                            ....|....|....|.
tr|C7PAL6|C7PAL6_CHIPD  YHGRGPIQLSWNYNYG
tr|A4CA61|A4CA61_9GAMM  YQGRGAIQLSYNYNYG
tr|D3EGU1|D3EGU1_9BACI  YYGRGPIMLSWNFNYG
tr|A9KHM6|A9KHM6_CLOPH  YHGRGPIQLSYNYNYG
tr|Q12JG0|Q12JG0_SHEDO  YYGRGALQLSYQSNYQ
tr|C9QEL9|C9QEL9_VIBOR  YYGRGIIQLSWNYNYG
tr|D0IKD1|D0IKD1_9VIBR  YYGRGVIQLSWNYNYG
tr|B5YMF3|B5YMF3_THAPS  FFGRGAIQTSWNYNYR
tr|B8C6K1|B8C6K1_THAPS  FFGRGAIQLSWNYNYI
tr|B5YN07|B5YN07_THAPS  FYGRGAIQLSWNYNYI
tr|B8LD35|B8LD35_THAPS  YFGRGSIQLSWNYNYI
tr|B8LD51|B8LD51_THAPS  FFGRGAIQLSWNYNYI
tr|B8BUZ0|B8BUZ0_THAPS  YFGRGAIQLSWNSNYL
tr|B8LD34|B8LD34_THAPS  FFGRGSIQLSHNFNYI
tr|Q8H6Y7|Q8H6Y7_PHYIN  YHGRGPIQLSWNYNYY
tr|D0NXB9|D0NXB9_PHYIN  YHGRGPIQLSWNYNYY
tr|D0NXB7|D0NXB7_PHYIN  YHGRGPIQIRWNYNYY
tr|Q23JY1|Q23JY1_TETTH  YYGRGPLQLSWNYNYA
tr|Q235U7|Q235U7_TETTH  YYGRGPLQLSWDYNYI
tr|A4VCP5|A4VCP5_TETTH  YYGRGPIQLSWDYNYK
tr|Q23VY2|Q23VY2_TETTH  YFGRGPVQLSWNYNYA
tr|Q8MD06|Q8MD06_LEUGL  YYGRGPMQLSWNYNYG
tr|Q8LK49|Q8LK49_LEUGL  YYGRGPMQLSWNYNYG
tr|Q9FUH3|Q9FUH3_VIGUS  YYGRGPIQISWNYNYG
tr|Q43685|Q43685_VIGUN  YYGRGPIQISWNYNYG
tr|Q6UZ78|Q6UZ78_PHAVU  YYGRGPIQISWNYNYG
tr|Q9LEB2|Q9LEB2_PHAVU  YYGRGPIQLSWNYNYA
tr|Q9FXL8|Q9FXL8_PSOTE  YYGRGPIQISWNYNYG
tr|O65331|O65331_ELAUM  YYSRGPMQLSWNYNYG
tr|Q5I6L0|Q5I6L0_HUMLU  YYGRGPMQLSWNYNYG
tr|B9HQY8|B9HQY8_POPTR  YYGRGPVQLSWNYNYG
tr|B9HQY7|B9HQY7_POPTR  YYGRGPVQLSWNYNYG
tr|A9PJS5|A9PJS5_9ROSI  YYGRGPVQLSWNYNYG
tr|B9HQZ2|B9HQZ2_POPTR  YYGRGPVQLSWNYNYG
tr|B9H1P7|B9H1P7_POPTR  YYGRGPMQLSWNYNYG
tr|B9SIC4|B9SIC4_RICCO  YYGRGPIQLTWNYNYG
tr|B0ZC08|B0ZC08_CASGL  YYGRGPIQLSWNYNYG
tr|Q9SDW1|Q9SDW1_PRUPE  YYGRGPIQLSWNYNYG
tr|Q4PJV8|Q4PJV8_9ROSA  YFGRGPIQLSWNYNYG
tr|Q43853|Q43853_ULMAM  YFGRGPIQLSWNYNYG
tr|Q8GUD7|Q8GUD7_HEVBR  YYGRGPIQLSWNYNYG
tr|Q949H3|Q949H3_HEVBR  YYGRGPIQLSWNYNYG
tr|Q9ZTT8|Q9ZTT8_GOSHI  YFGRGPMQLSWNYNYG
tr|Q8VXF1|Q8VXF1_MUSAC  YYGRGPIQISFNYNYG
tr|B5TYQ0|B5TYQ0_MUSAC  YYGRGPIQISFNYNYG
tr|Q8VXF0|Q8VXF0_MUSAC  YYGRGPIQISFNYNYG
tr|Q93WX9|Q93WX9_MUSAC  YYGRGPIQISFNYNYG
tr|B6UYK7|B6UYK7_MUSPR  YYGRGPIQISFNYNYG
tr|B6UYK6|B6UYK6_MUSPR  YYGRGPIQISFNYNYG
tr|C3VD22|C3VD22_MUSPR  YYGRGPIQISFNYNYG
tr|B5TYQ1|B5TYQ1_MUSAC  YYGRGPIQISYNYNYG
tr|B9ZZZ5|B9ZZZ5_VACCO  YYGRGPIQISYNYNYG
tr|Q9SPU0|Q9SPU0_PETCR  YYGRGPIQISYNYNYG
tr|B2DD07|B2DD07_CITUN  YFGRGPIQISYNYNYG
tr|Q6RH76|Q6RH76_CAPAN  YFGRGPIQISYNYNYG
tr|P93154|P93154_GOSHI  YFGRGPIQISYNYNYG
tr|Q9FS45|Q9FS45_VITVI  YYGRGPIQISYNYNYG
tr|A5AT01|A5AT01_VITVI  YYGRGPIQISYNYNYG
tr|D1HC88|D1HC88_VITVI  YYGRGPIQISYNYNYG
tr|Q9ZTK4|Q9ZTK4_VITVI  YYGRGPIQISYNYNYG
tr|A5AT00|A5AT00_VITVI  YYGRGPIQISYNYNYG
tr|D0EWF1|D0EWF1_CAMSI  YYGRGPIQISHNYNYG
tr|P93680|P93680_PERAE  YYGRGPIQISYNYNYG
tr|A3QRB6|A3QRB6_VITVI  YYGRGPIQISYNYNYG
tr|Q546P8|Q546P8_VITVI  YYGRGPIQISYNYNYG
tr|A3QRB7|A3QRB7_VITVI  YYGRGPIQISYNYNYG
tr|Q6IVX2|Q6IVX2_9CARY  YYGRGPIQISYNFNYG
tr|Q6IVX4|Q6IVX4_9CARY  YYGRGPIQISYNFNYG
tr|P94084|P94084_MEDSA  YYGRGPIQISWNYNYG
tr|P93327|P93327_MEDTR  YYGRGPIQISWNYNYG
tr|Q5RLX9|Q5RLX9_MEDSA  YYGRGPIQISWNYNYG
tr|Q7X9F5|Q7X9F5_9FABA  YYGRGPIQISWNYNYG
tr|Q9ZP10|Q9ZP10_CICAR  YYGRGPIQLSWNYNYG
```

```
tr|Q4JLT7|Q4JLT7_CAPAN  YFGRGPIQISYNYNYG
tr|Q9AVA9|Q9AVA9_CUCME  YFGRGPIQISYNYNCG
tr|B9VRK7|B9VRK7_CAPAN  YFGRGPIQISYNYNYG
tr|Q9FEW1|Q9FEW1_NICSY  YFGRGPIQISHNYNYG
tr|Q5W1I6|Q5W1I6_NICGL  YFGRGPIQISHNYNYG
tr|O81144|O81144_SOLTU  YFGRGPIQISYNYNYG
tr|O81145|O81145_SOLTU  YFGRGPIQISHNYNYG
tr|Q84LQ7|Q84LQ7_SOLDU  YFGRGPIQISHNYNYG
tr|Q6IV10|Q6IV10_DRORT  YFGRGPIQISHNYNYG
tr|Q2HPK8|Q2HPK8_SOLTU  YFGRGPIQISHNYNYG
tr|Q9SQL3|Q9SQL3_POAPR  YFGRGPIQISYNYNYG
tr|Q9SQL4|Q9SQL4_POAPR  YFGRGPIQISFNYNYG
tr|Q6T484|Q6T484_WHEAT  YFGRGPIQISHNYNYG
tr|Q8W427|Q8W427_WHEAT  YFGRGPIQISHNYNYG
tr|Q41539|Q41539_WHEAT  YFGRGPIQISYNYNYG
tr|Q9AXR9|Q9AXR9_SECCE  YFGRGPIQISYNYNYG
tr|B1B6T0|B1B6T0_BROIN  YFGRGPIQISHNYNYG
tr|B8R3R6|B8R3R6_FESAR  YYGRGPIQISYNYNYG
tr|Q4Z8L7|Q4Z8L7_WHEAT  YYGRGPIQLSHNYNYG
tr|Q4Z8L8|Q4Z8L8_WHEAT  YYGRGPIQLSHNYNYG
tr|Q6JBN6|Q6JBN6_ZEAMP  YYGRGPIQISYNYNYG
tr|Q6JBM9|Q6JBM9_ZEAMP  YYGRGPIQISYNYNYG
tr|Q6JBN3|Q6JBN3_ZEAMP  YYGRGPIQISYNYNYG
tr|B8QVK2|B8QVK2_ZEAMP  YYGRGPIQISYNYNYG
tr|Q6JBN5|Q6JBN5_ZEAMP  YYGRGPIQISYNYNYG
tr|B8QVF2|B8QVF2_ZEAMP  YYGRGPIQISYNYNYG
tr|B8QVF1|B8QVF1_ZEAMP  YYGRGPIQISYNYNYG
tr|B8QVK0|B8QVK0_ZEAMP  YYGRGPIQISYNYNYG
tr|B8QVG9|B8QVG9_ZEAMP  YYGRGPIQISYNYNYG
tr|B8QVL4|B8QVL4_ZEAMP  YYGRGPIQISYNYNYG
tr|B8QVH2|B8QVH2_ZEAMP  YYGRGPIQISYNYNYG
tr|B8QVF8|B8QVF8_ZEAMP  YYGRGPIQISYNYNYG
tr|B8QVM1|B8QVM1_ZEAMP  YYGRGPIQISYNYNYG
tr|Q6JBM4|Q6JBM4_ZEADI  YYGRGPIQISYNYNYG
tr|Q6JBN0|Q6JBN0_ZEAMP  YYGRGPIQISYNYNYG
tr|B8QVL5|B8QVL5_ZEAMP  YYGRGPIQISYNYNYG
tr|B8QVH0|B8QVH0_ZEAMP  YYGRGPIQISYNYNYG
tr|B8QVI0|B8QVI0_ZEAMP  YYGRGPIQISYNYNYG
tr|B8QVK5|B8QVK5_ZEAMP  YYGRGPIQISYNYNYG
tr|B8QVF9|B8QVF9_ZEAMP  YYGRGPIQISYNYNYG
tr|Q6JBN1|Q6JBN1_ZEAMP  YYGRGPIQISYNYNYG
tr|B8QVF0|B8QVF0_ZEAMP  YYGRGPIQISYNYNYG
tr|B8QVH5|B8QVH5_ZEAMP  YYGRGPIQISYNYNYG
tr|B8QVI5|B8QVI5_ZEAMP  YYGRGPIQISYNYNYG
tr|B8QVH1|B8QVH1_ZEAMP  YYGRGPIQISYNYNYG
tr|B8QVI2|B8QVI2_ZEAMP  YYGRGPIQISYNYNYG
tr|Q6JBN4|Q6JBN4_ZEAMP  YYGRGPIQISYNYNYG
tr|B8QVM0|B8QVM0_ZEAMP  YYGRGPIQISYNYNYG
tr|B8QVH4|B8QVH4_ZEAMP  YYGRGPIQISYNYNYG
tr|B8QVH3|B8QVH3_ZEAMP  YYGRGPIQISYNYNYG
tr|B8QVG8|B8QVG8_ZEAMP  YYGRGPIQISYNYNYG
tr|B8QVL7|B8QVL7_ZEAMP  YYGRGPIQISYNYNYG
tr|B8QVI1|B8QVI1_ZEAMP  YYGRGPIQISYNYNYG
tr|B8QVJ5|B8QVJ5_ZEAMP  YYGRGPIQISYNYNYG
tr|B8QVH7|B8QVH7_ZEAMP  YYGRGPIQISYNYNYG
tr|B8QVH8|B8QVH8_ZEAMP  YYGRGPIQISYNYNYG
tr|Q6JBM3|Q6JBM3_ZEADI  YYGRGPIQISYNYNYG
tr|Q6JBM1|Q6JBM1_ZEADI  YYGRGPIQISYNYNYG
tr|Q6JBL7|Q6JBL7_ZEADI  YYGRGPIQISYNYNYG
tr|Q6JBM5|Q6JBM5_ZEADI  YYGRGPIQISYNYNYG
tr|Q6JBL9|Q6JBL9_ZEADI  YYGRGPIQISYNYNYG
tr|Q41794|Q41794_MAIZE  YYGPRAIQISININIG
tr|C5YYH5|C5YYH5_SORBI  YYGRGPIQISYNYNYG
tr|C6JSV0|C6JSV0_SORBI  YYGRGPIQISYNYNYG
tr|C5YGD9|C5YGD9_SORBI  YYGRGPIQISYNYNYG
tr|A2YH55|A2YH55_ORYSI  YYGRGPIQISYNYNYG
tr|Q42995|Q42995_ORYSA  YYGRGPIQISYNYNYG
tr|Q40667|Q40667_ORYSA  YYGRGPIQISYNYNYG
tr|Q6U5G8|Q6U5G8_ORYSJ  YYGRGPIQITYNYSYG
tr|Q0D9D3|Q0D9D3_ORYSJ  YYGRGPIQITYNYNYG
tr|A2YH54|A2YH54_ORYSI  YYGRGPIQITYNYNYG
tr|Q40668|Q40668_ORYSA  YYGRGPIQITYNYNYG
tr|Q2HJJ5|Q2HJJ5_MUSPR  YYGRGPIQLSYNFNYG
tr|A7UC81|A7UC81_ORYSI  YYGRGPIQLSYNFNYG
tr|A2XI16|A2XI16_ORYSI  YYGRGPIQLSYNFNYG
tr|A3AJ48|A3AJ48_ORYSJ  YYGRGPIQLSYNFNYG
tr|Q6DUK0|Q6DUK0_9CARY  YYGRGPIQISYNYNYG
```

```
tr|Q38777|Q38777_ALLSA  YYGRGPIQISYNYNYG
tr|Q38776|Q38776_ALLSA  YYGRGPIQISYNYNYG
tr|A2V800|A2V800_ANACO  YYGRGPIQISYNYNYG
tr|Q6IVX8|Q6IVX8_9CARY  YYGRGPIQLSYNYNYG
tr|Q6SZS3|Q6SZS3_ORYSA  YFGRGPIQLSYNYNYG
tr|A2Y4F6|A2Y4F6_ORYSI  YFGRGPIQLSYNYNYG
tr|Q8VWZ5|Q8VWZ5_ORYSA  YFGRGPIQLSYNYNYG
tr|B9FPH6|B9FPH6_ORYSJ  YFGRGPIQLSYNYNYG
tr|Q42992|Q42992_ORYSA  YFGRGPIQLSYNYNYG
tr|Q6SPQ7|Q6SPQ7_BAMOL  YFGRGPIQLSYNYNYG
tr|C5YXM4|C5YXM4_SORBI  YFGRGPIQISFNYNYG
tr|B6TR38|B6TR38_MAIZE  YFGRGPIQISFNYNYG
tr|Q8W428|Q8W428_WHEAT  YYGRGPIQLTHNYNYG
tr|Q42970|Q42970_ORYSA  YYGRGPIQLSFNFNYG
tr|Q42839|Q42839_HORVU  YYGRGPIMLSWNYNYG
tr|C0LNR1|C0LNR1_9MAGN  YYGRGPMQISYNYNYG
tr|B8YPL6|B8YPL6_9MAGN  YYGRGPMQISYNYNYG
tr|Q9AVA7|Q9AVA7_CUCME  YYGRGPFQITYNYNYG
tr|Q9FPM1|Q9FPM1_FRAAN  YYGRGPIQLTHNYNYG
tr|Q944U5|Q944U5_FRAAN  YYGRGPIQLTHNYNYG
tr|B9VQ31|B9VQ31_PYRPY  YYGRGPIQLTHNYNYG
tr|Q8RVN0|Q8RVN0_MALDO  YYGRGPIQLTHNYNYG
tr|Q8RVN2|Q8RVN2_MALDO  YYGRGPIQLTHNYNYG
tr|B8XR33|B8XR33_9ROSA  YYGRGPIQLTHNYNYA
tr|Q8RVN1|Q8RVN1_MALDO  YYGRGPIQLTHNYNYG
tr|B9S6S0|B9S6S0_RICCO  YYGRGPIQLTHNYNYG
tr|B5TYQ3|B5TYQ3_9ROSI  YFGRGPIQLTHNYNYG
tr|Q42428|Q42428_CASSA  YYGRGPIQLTHNYNYG
tr|Q7X9F4|Q7X9F4_9FABA  YYGRGPIQLTYNYNYG
tr|Q7X9F6|Q7X9F6_9FABA  YYGRGPIQITHNYNYG
tr|Q9SDY6|Q9SDY6_SOYBN  YYGRGPIQLTHNYNYG
tr|Q1W6C5|Q1W6C5_9CARY  YYGRGPIQLTHNYNYA
tr|B3VFX0|B3VFX0_9CARY  YYGRGPIQLTHNYNYA
tr|O80404|O80404_9ROSI  YYGRGPIQLTHNYNYG
tr|A7UGA7|A7UGA7_CUCMO  YYGRGPIQLTHNYNYG
tr|Q207U1|Q207U1_MOMCH  YYGRGPIQLTHNYNYG
tr|Q9AVA8|Q9AVA8_CUCME  YYGRGPIQLTHNYNYG
tr|A1BQQ0|A1BQQ0_CUCSA  YYGRGPIQLTHNYNYG
tr|Q9XFK6|Q9XFK6_HUMLU  YYGRGPIQLTHNYNYN
tr|Q43184|Q43184_SOLTU  YFGRGPIQISYNYNYG
tr|Q42878|Q42878_SOLLC  YFGRGPIQISYNYNYG
tr|Q9ZWS3|Q9ZWS3_TOBAC  YFGRGPIQISYNYNYG
tr|Q7X9R8|Q7X9R8_EUOEU  YYGRGPLQLSWNYNYA
tr|Q7Y237|Q7Y237_EUOEU  YYGRGPLQLSWNYNYA
tr|Q7Y238|Q7Y238_EUOEU  YYGRGPIQLTWNYNYE
tr|Q7X9R9|Q7X9R9_EUOEU  YYGRGPLQLTWNYNYA
tr|Q9M7F5|Q9M7F5_BOEPA  YYGRGPMQLSWNYNYG
tr|Q9M7H2|Q9M7H2_BOEDR  YYGRGPMQLSWNYNYG
tr|Q9M7G6|Q9M7G6_ARAGU  YYGRGPMQLSWNYNYG
tr|Q9M7H5|Q9M7H5_ARAGU  YYGRGPMQLSWNYNYG
tr|Q9M7H0|Q9M7H0_ARAFE  YYGRGPMQLSWNYNYG
tr|Q9M7G3|Q9M7G3_9BRAS  YYGRGPMQLSWNYNYG
tr|Q9M7G0|Q9M7G0_ARALI  YYGRGPMQLSWNYNYG
tr|Q9M7F7|Q9M7F7_ARAMC  YYGRGPMQLSWNYNYG
tr|Q9M7F9|Q9M7F9_9BRAS  YYGRGPMQLSWNYNYG
tr|Q9M7G1|Q9M7G1_ARALI  YYGRGPIQLSWNYNYG
tr|Q9M7F4|Q9M7F4_BOEPA  YYGRGPMQLSWNYNYG
tr|Q9M7F6|Q9M7F6_ARAMC  YYGRGPXQLSWNYNYG
tr|Q9M7F8|Q9M7F8_ARAMC  YYGRGPMQLSWNYNYG
tr|Q9M7G4|Q9M7G4_9BRAS  YYGRGPMQLSWNYNYG
tr|Q9M7H7|Q9M7H7_ARAHO  YYGRGPIQLSWNYNYG
tr|Q9M7G2|Q9M7G2_ARALI  YYGRGPIQLSWNYNYG
tr|Q9M7H1|Q9M7H1_ARAFE  YYGRGPIQLSWNYNYG
tr|Q9M7G7|Q9M7G7_ARAGL  YYGRGPMQLSWNYNYG
tr|Q9M7H9|Q9M7H9_ARAAL  YYGRGPMQLSWNYDYG
tr|Q9M7H3|Q9M7H3_ARABL  YYGRGPMQLSWNYNYG
tr|Q9SXJ1|Q9SXJ1_ARAGE  YYGRGPMQLTWNYNYG
tr|Q9M7G5|Q9M7G5_9BRAS  YYGRGPIQLSWNYNYG
tr|Q9M7G9|Q9M7G9_ARAGL  YYGRGPMQLTWNYNYG
tr|Q9M7H4|Q9M7H4_ARABL  YYGRGPMQLTWNYNYG
tr|Q1L0Q6|Q1L0Q6_BOEDR  YYGRGPMQLSWNYNYG
tr|Q9M7G8|Q9M7G8_ARAGL  YYGRGPMQLLWNYNYG
tr|A9P2N2|A9P2N2_PICSI  YYGRGPVQISWNYNYG
tr|A9NPF6|A9NPF6_PICSI  YYGRGPVQISWNYNYG
tr|A9NUA7|A9NUA7_PICSI  YYGRGPVQISWNYNYG
tr|A9NLC7|A9NLC7_PICSI  YYGRGPIQISWNYNYG
tr|Q76JH6|Q76JH6_CRYJA  YYGRGPIQLSWNYNYI
```

```
tr|Q403K0|Q403K0_CRYJA  YYGRGPIQLSWNYNYI
tr|Q76JK0|Q76JK0_CRYJA  YYGRGPIQLSWNYNYI
tr|Q76JJ7|Q76JJ7_CRYJA  YYGRGPIQLSWNYNYI
tr|A2PZ52|A2PZ52_CRYJA  YYGRGPIQLSWNYNYI
tr|Q76IR2|Q76IR2_TAXDI  YYGRGPIQLSWNYNYI
tr|A2PZ63|A2PZ63_CHAOB  YYGRGPIQLSWNYNYI
tr|A2PZ64|A2PZ64_CHAOB  YYGRGPIQLSWNYNYI
tr|A2PZ61|A2PZ61_9CONI  YYGRGPIQLSWNYNYI
tr|A2PZ59|A2PZ59_9CONI  YYGRGPIQLSWNYNYI
tr|A2PZ60|A2PZ60_9CONI  YYGRGPIQLSWNYNYI
tr|A2PZ58|A2PZ58_9CONI  YYGRGPIQLSWNYNYI
tr|Q6E6M9|Q6E6M9_PICAB  YHGRGPIQLTGDYNYK
tr|Q6SSF1|Q6SSF1_PICAB  YHGRGPIQLAGDYNYK
tr|C0PQM5|C0PQM5_PICSI  YHGRGPIQLTGDYNYK
tr|A9NXF9|A9NXF9_PICSI  YHGRGPIQLTGDYNYK
tr|O04276|O04276_PINST  YRGRGPIQLTGDYNYK
tr|C0PST0|C0PST0_PICSI  YYGRGPIQLTGKNNYE
tr|A9NTQ9|A9NTQ9_PICSI  YYGRGPIQLTGKNNYE
tr|A9NU92|A9NU92_PICSI  YYGRGPIQLTGKSNYE
tr|C0P3M6|C0P3M6_MAIZE  YYGRGPIQLTHEYNYR
tr|B6SZC6|B6SZC6_MAIZE  YYGRGPIQLTHEYNYR
tr|C5WZU7|C5WZU7_SORBI  YYGRGPIQLTHEYNYR
tr|C0HEI0|C0HEI0_MAIZE  YYGRGPIQLTHKYNYR
tr|B4F9D5|B4F9D5_MAIZE  YYGRGPIQLTHKYNYR
tr|B6T6W1|B6T6W1_MAIZE  YYGRGPIQLTHKYNYR
tr|A2XC48|A2XC48_ORYSI  YYGRGPIQLTHKYNYQ
tr|O04272|O04272_ORYSA  YYGRRPYTLLISTTTS
tr|Q8H0C9|Q8H0C9_VIGUN  YYGRGPIQLTHNYNYN
tr|O81934|O81934_CANEN  YYGRGPIQLTHNYNYA
tr|Q42515|Q42515_ARAHY  YYGRGPIQLTSNSNYQ
tr|Q43752|Q43752_CITSI  YYGRGPIQLSWNYNYL
tr|Q8H986|Q8H986_CITJA  YYGRGPIQLTWNYNYL
tr|Q8H985|Q8H985_CITJA  YYGRGPIQLTWNCNYL
tr|O04222|O04222_HELAN  YFGRGPIQLTNNNNYG
tr|Q8W4Z2|Q8W4Z2_ORYSI  YYGRGPIQLTGQSNYQ
tr|O24007|O24007_ORYSA  YYGRGPIQLTGQSNYQ
tr|A2Z9V6|A2Z9V6_ORYSI  YYGRGPIQLTGQSNYQ
tr|O04271|O04271_ORYSA  YYGRGPIQLTGQSNYQ
tr|Q7G1L6|Q7G1L6_ORYSA  YYGRGPIQLTGQSNYQ
tr|D2CVR3|D2CVR3_HORVD  YYGRGPIQLTGQSNYD
tr|Q43765|Q43765_HORVU  YYGRGPIQLTGQSNYD
tr|Q9AXR8|Q9AXR8_SECCE  YYGRGPIQLTGRSNYD
tr|Q8W429|Q8W429_WHEAT  YYGRGPIQLTGRSNYD
tr|Q43764|Q43764_HORVU  YYGRGPIQLTGRSNYD
tr|C8CG67|C8CG67_9POAL  YYGRGPIQLTGRSNYD
tr|Q9LEH7|Q9LEH7_HORVU  YYGRGPIQLTGQSNYD
tr|Q43834|Q43834_SOLTU  YYGRGPIQLTHQSNYE
tr|Q9SBJ9|Q9SBJ9_SOLTU  YYGRGPIQLTHQSNYE
tr|Q43835|Q43835_SOLTU  YYGRGPIQLTHQSNYE
tr|O22568|O22568_SOLTU  YYGRGPIQLTHQSNYE
tr|Q7Y0S1|Q7Y0S1_SOLLC  YYGRGPIQLTHQSNYE
tr|O82552|O82552_CAPAN  YYGRGPIQLTHRSNYE
tr|B2NIX6|B2NIX6_CAPCH  YYGRGPIQLTHRSNYE
tr|C5IDR3|C5IDR3_CAPCH  FYGRGPIQLTGQSNYE
tr|Q6IV09|Q6IV09_DRORT  YYGRGPIQLTNRNNYE
tr|Q41795|Q41795_MAIZE  YFGRGPIQLSYNYNYG
tr|B9HQY9|B9HQY9_POPTR  YCGRGPIQLSWNYNYG
tr|B9HQY6|B9HQY6_POPTR  YCGRGPIQLSWNYNYG
tr|B9HQZ1|B9HQZ1_POPTR  YCGRGPIQLSWNYNYG
tr|O65330|O65330_ELAUM  YYGRGPIQLSWNYNYG
tr|Q9SQF7|Q9SQF7_BRAJU  YYGRGPMMLSWNYNYG
tr|Q2HZ51|Q2HZ51_BRAJU  YYGRGPIMLSWNYNYG
tr|Q42421|Q42421_BETVU  YYGRGPVQLTWNFNYG
tr|B8QVG3|B8QVG3_ZEAMP  YYGRGPIQISYNYNYG
tr|Q43852|Q43852_WHEAT  YYGRGPIQLTHNYNYG
tr|Q9LE03|Q9LE03_CUCME  YYGRGPMQLKGNQNYG
tr|Q6QNA9|Q6QNA9_MUSBA  YFGRGPIQISHNYNYG
tr|Q6QNB2|Q6QNB2_MUSAC  YFGRGPIQISHNYNYG
tr|Q6R8K3|Q6R8K3_MUSAC  YFGRGPIQISHNYNYG
tr|C1K2M3|C1K2M3_PANGI  YYGRGPIQLTHNYNYG
tr|Q5I707|Q5I707_LINUS  YYGRGPIQLTHNYNYG
tr|B9FPH7|B9FPH7_ORYSJ  YHARGPMQIAYNYNYG
tr|B9H1P8|B9H1P8_POPTR  YYGRGPLQLRWNYNYG
tr|Q9ZT61|Q9ZT61_SAMNI  YNRRGPIQLTHNYNYG
tr|Q9SYS5|Q9SYS5_SAMNI  YNRRGPIQLTHNYNYG
tr|Q9SYS4|Q9SYS4_SAMNI  YNRRGPIQLTHNYYYG
tr|Q944B9|Q944B9_SAMNI  YNSRGPIQLTHNYNYG
```

tr|Q9ZT60|Q9ZT60_SAMNI	YNSRGPIQLTHNYYYG
tr|Q944B8|Q944B8_SAMNI	YNSRGPIQLTHNYNYG
tr|A7XQ02|A7XQ02_MORAL	YNSRGAVQLTHNYNYG
tr|Q84LK1|Q84LK1_FICCA	YNSRGPIQLTHNHNYG
tr|Q2VAC7|Q2VAC7_FICAW	YNRRGPIQLTHNHNYG
tr|B9GTA5|B9GTA5_POPTR	YKGRGPIQLSWNYNYG
tr|B9I9A3|B9I9A3_POPTR	YKGRGPIQLSWNYNYG
tr|C6TI96|C6TI96_SOYBN	YKGRGPIQLSWNYNYG
tr|A5BE14|A5BE14_VITVI	YKGRGPIQLSWNYNYG
tr|D1IUL3|D1IUL3_VITVI	YKGRGPIQLSWNYNYG
tr|B9RN36|B9RN36_RICCO	YKGRGPIQLSWNFNYG
tr|Q43322|Q43322_ARAHY	YKGRGPIQLSWNYNYG
tr|C6TNB0|C6TNB0_SOYBN	YKGRGPIQLSWNYNYG
tr|Q9SPT9|Q9SPT9_PETCR	YQGRGPIQLSWNYNYG
tr|Q9SPT8|Q9SPT8_PETCR	YQGRGPIQLSWNYNYG
tr|B2X051|B2X051_FRAAN	YKGRGPIQLSWNYNYG
tr|Q9SBT5|Q9SBT5_FRAAN	YKGRGPIQLSWNYNYG
tr|A5JW48|A5JW48_FRAAN	YKGRGPIQLSWNYNYG
tr|Q9ZSI6|Q9ZSI6_ARATH	YKGRGPIQLSWNYNYG
tr|Q9FZ25|Q9FZ25_ARATH	YQGRGPIQLSWNYNYG
tr|Q6AT95|Q6AT95_ORYSJ	YHGRGPIQLSWNFNYG
tr|A2Y074|A2Y074_ORYSI	YHGRGPIQLSWNFNYG
tr|C5YZL4|C5YZL4_SORBI	YHGRGPIQLSWNFNYG
tr|Q9ZSE8|Q9ZSE8_CYNDA	YHGRGPIQLSWNFNYG
tr|O22520|O22520_CYNDA	YHGRGPIQLSWNFNYG
tr|Q9ZSE7|Q9ZSE7_CYNDA	YHGRGPIQLSWNFNYG
tr|C5XHZ4|C5XHZ4_SORBI	YHGRGPIQLSWNFNYG
tr|A2WNN5|A2WNN5_ORYSI	YHGRGPIQISWNFNYG
tr|O22425|O22425_ORYSA	YHGQGPIQISWNFNYG
tr|A9NS04|A9NS04_PICSI	YHGRGALPLYWNYNYG
tr|C0PPT3|C0PPT3_PICSI	YHGRGALPLYWNYNYG
tr|A9NT20|A9NT20_PICSI	YHGRGALPVYWNYNYG
tr|B4G1H3|B4G1H3_MAIZE	YYGRGAIPVYWNYNYG
tr|C5X4J4|C5X4J4_SORBI	YYGRGAIPVYWNYNYG
tr|Q0J0L1|Q0J0L1_ORYSJ	YYGRGAIPVFWNYNYG
tr|A2Z2P0|A2Z2P0_ORYSI	YYGRGAIPVFWNYNYG
tr|D2KZ17|D2KZ17_9ARAE	YHGRGALPVYWNYNYG
tr|A2Q575|A2Q575_MEDTR	YYGRGAIPIYWNYNYG
tr|Q76KW5|Q76KW5_PEA	YYGRGAIPIYWNYNYG
tr|C6TD22|C6TD22_SOYBN	YYGRGAIPIFWNYNYG
tr|C0SUT2|C0SUT2_ARATH	YYGRGALPIYWNFNYG
tr|Q9MA41|Q9MA41_ARATH	YYGRGALPIYWNFNYG
tr|A8IXW1|A8IXW1_BRACM	YYGRGALPIYWNFNYG
tr|A8IXW2|A8IXW2_BRACM	YYGRGALPIYWNFNYG
tr|Q7X7Q1|Q7X7Q1_GOSHI	YYGRGALPIYWNYNYG
tr|Q6JX03|Q6JX03_GOSHI	YHGRGALPIYWNYNYG
tr|Q6UA09|Q6UA09_GOSBA	YHGRGALPIYWNYNYG
tr|B5G510|B5G510_GOSBA	YHGRGALPIYWNYNYG
tr|Q6JX04|Q6JX04_GOSHI	YHGRGALPIYWNYNYG
tr|B5G507|B5G507_GOSBA	YHGRGALPIYWNYNYG
tr|B5G509|B5G509_GOSBA	YHGRGALPIYWNYNYG
tr|B5G508|B5G508_GOSBA	YHGRGALPIYWNYNYG
tr|B9VQ32|B9VQ32_PYRPY	YHGRGALPLYWNYNYG
tr|A9P7W2|A9P7W2_POPTR	YHGRGALPLYWNYNYG
tr|B9RZM8|B9RZM8_RICCO	YHGRGALPLYWNYNYG
tr|A5B1C7|A5B1C7_VITVI	YFGRGALPIYWNYNYG
tr|D1HNU6|D1HNU6_VITVI	YFGRGALPIYWNYNYG
tr|B9RPJ5|B9RPJ5_RICCO	YYGRGALPIYWNYNYG
tr|D1J1H7|D1J1H7_VITVI	YYGRGALPIFWNYNYG
tr|Q9LSP9|Q9LSP9_ARATH	YHGRGALPVYWNYNYG
tr|C6TAW6|C6TAW6_SOYBN	YYGRGAIPLYWNYNYG
tr|C6TIR8|C6TIR8_SOYBN	YYGRGAIPLYWNYNYG
tr|B9IA90|B9IA90_POPTR	YYGRGAIPIFWNYNYG
tr|A9PHL3|A9PHL3_POPTR	YYGRGAIPIFWNYNYG
tr|Q1W6C4|Q1W6C4_9CARY	YYGRGALPIYWNYNYG
tr|B3VFX1|B3VFX1_9CARY	YYGRGALPIYWNYNYG
tr|A2YX68|A2YX68_ORYSI	YYGRGALPVYWNYNYG
tr|Q84QQ7|Q84QQ7_ORYSJ	YYGRGALPVYWNYNYG
tr|C5YIM8|C5YIM8_SORBI	YYGRGALPVYWNYNYG
tr|A9TSG6|A9TSG6_PHYPA	YYGRGAFPLYWNYNYG
tr|A9SXI5|A9SXI5_PHYPA	YYGRGAFPLYWNYNYG
tr|A9TSE2|A9TSE2_PHYPA	YHGRGAFPLYWNFNYG
tr|C5KAJ7|C5KAJ7_9ALVE	YYGRGALQLSYNFNYG
tr|C5LPC3|C5LPC3_9ALVE	YYGRGALQLSWNYNYG
tr|C5KT12|C5KT12_9ALVE	YSGRGAKQISYNYNYG
tr|C5KMD1|C5KMD1_9ALVE	YFGRGAAQISWNYNYG
tr|Q2SMW1|Q2SMW1_HAHCH	YFGRGAKQLSYNYNYG

tr|A4BH92|A4BH92_9GAMM YFGRGAKQLSYNYNYG
tr|A8AIM5|A8AIM5_CITK8 YFGRGAKQLSYNYNYG
tr|D2ZAE8|D2ZAE8_9ENTR YFGRGAKQLSYNYNYG
tr|B4TYE6|B4TYE6_SALSV YFGRGAKQLSYNYNYG
tr|B5C920|B5C920_SALET YFGRGAKQLSYNYNYG
tr|B5NMR8|B5NMR8_SALET YFGRGAKQLSYNYNYG
tr|B3YM59|B3YM59_SALET YFGRGAKQLSYNYNYG
tr|Q8ZRN8|Q8ZRN8_SALTY YFGRGAKQLSYNYNYG
tr|D0ZKY9|D0ZKY9_SALT1 YFGRGAKQLSYNYNYG
tr|B5R425|B5R425_SALEP YFGRGAKQLSYNYNYG
tr|B5FJ33|B5FJ33_SALDC YFGRGAKQLSYNYNYG
tr|B5C3J2|B5C3J2_SALET YFGRGAKQLSYNYNYG
tr|B5MUQ3|B5MUQ3_SALET YFGRGAKQLSYNYNYG
tr|B5F8U7|B5F8U7_SALA4 YFGRGAKQLSYNYNYG
tr|B5PN43|B5PN43_SALET YFGRGAKQLSYNYNYG
tr|B4TK61|B4TK61_SALHS YFGRGAKQLSYNYNYG
tr|B5P9D5|B5P9D5_SALET YFGRGAKQLSYNYNYG
tr|B5R5I9|B5R5I9_SALG2 YFGRGAKQLSYNYNYG
tr|C0Q6K9|C0Q6K9_SALPC YFGRGAKQLSYNYNYG
tr|B4SV15|B4SV15_SALNS YFGRGAKQLSYNYNYG
tr|A9N0T7|A9N0T7_SALPB YFGRGAKQLSYNYNYG
tr|B5Q115|B5Q115_SALHA YFGRGAKQLSYNYNYG
tr|B4A398|B4A398_SALNE YFGRGAKQLSYNYNYG
tr|Q8Z999|Q8Z999_SALTI YFGRGAKQLSYNYNYG
tr|Q57T22|Q57T22_SALCH YFGRGAKQLSYNYNYG
tr|B5MSD4|B5MSD4_SALET YFGRGAKQLSYNYNYG
tr|Q5PD79|Q5PD79_SALPA YFGRGAKQLSYNYNYG
tr|B5BAP3|B5BAP3_SALPK YFGRGAKQLSYNYNYG
tr|B5N8A4|B5N8A4_SALET YFGRGAKQLSYNYNYG
tr|C9X780|C9X780_SALTD YFGRGAKQLSYNYNYG
tr|A9MPH5|A9MPH5_SALAR YFGRGAKQLSYNYNYG
tr|C9XWB9|C9XWB9_CROTZ YFGRGAKQLSYNYNYG
tr|A7MEM6|A7MEM6_ENTS8 YFGRGAKQLSYNYNYG
tr|B0U0H9|B0U0H9_FRAP2 YFGRGAKQLSYNYNYG
tr|C6YSN8|C6YSN8_9GAMM YFGRGAKQLSYNYNYG
tr|Q5E5Z2|Q5E5Z2_VIBF1 YFGRGAKQLSYNYNYG
tr|B5FDD8|B5FDD8_VIBFM YFGRGAKQLSYNYNYG
tr|C9Q4P1|C9Q4P1_9VIBR YFGRGAKQLSYNYNYG
tr|Q1ZMW5|Q1ZMW5_PHOAS YFGRGAKQLSYNYNYG
tr|Q0ZKA9|Q0ZKA9_AERHY YFGRGAKQLSYNYNYG
tr|A0KGX5|A0KGX5_AERHH YFGRGAKQLSYNYNYG
tr|A4SQY0|A4SQY0_AERS4 YFGRGAKQLSYNYNYG
tr|B1KH86|B1KH86_SHEWM YFGRGAKQLSYNYNYG
tr|C8Q541|C8Q541_9ENTR YFGRGAKQLSYNFNYG
tr|Q7NWT2|Q7NWT2_CHRVO YYGRGAKQLSYNYNYG
tr|A6D262|A6D262_9VIBR YFGRGAKQLSYHFNYG
tr|D0Z4N1|D0Z4N1_LISDA YFGRGAKQLSYHFNYG
tr|Q6LQ48|Q6LQ48_PHOPR YFGRGAKQLSYHFNYG
tr|Q1Z8D8|Q1Z8D8_PHOPR YFGRGAKQLSYHFNYG
tr|A0KKT5|A0KKT5_AERHH YFGRGAKQLSYHFNYG
tr|A4SMT5|A4SMT5_AERS4 YFGRGAKQLSYHFNYG
tr|D0HRW5|D0HRW5_VIBCH YFGRGAKQLSYHFNYG
tr|C6RVV6|C6RVV6_VIBCH YFGRGAKQLSYHFNYG
tr|A1EIH9|A1EIH9_VIBCH YFGRGAKQLSYHFNYG
tr|Q9KTW1|Q9KTW1_VIBCH YFGRGAKQLSYHFNYG
tr|C3NTC5|C3NTC5_VIBCJ YFGRGAKQLSYHFNYG
tr|A1F0S9|A1F0S9_VIBCH YFGRGAKQLSYHFNYG
tr|A2PEJ3|A2PEJ3_VIBCH YFGRGAKQLSYHFNYG
tr|A3GK58|A3GK58_VIBCH YFGRGAKQLSYHFNYG
tr|C3LT22|C3LT22_VIBCM YFGRGAKQLSYHFNYG
tr|A3GWU6|A3GWU6_VIBCH YFGRGAKQLSYHFNYG
tr|C6YDY1|C6YDY1_VIBCH YFGRGAKQLSYHFNYG
tr|C2JA78|C2JA78_VIBCH YFGRGAKQLSYHFNYG
tr|C2IIH7|C2IIH7_VIBCH YFGRGAKQLSYHFNYG
tr|A5F3F2|A5F3F2_VIBC3 YFGRGAKQLSYHFNYG
tr|A6XTA3|A6XTA3_VIBCH YFGRGAKQLSYHFNYG
tr|D0I240|D0I240_VIBCH YFGRGAKQLSYHFNYG
tr|A2PP35|A2PP35_VIBCH YFGRGAKQLSYHFNYG
tr|C2HVC2|C2HVC2_VIBCH YFGRGAKQLSYHFNYG
tr|A6Y174|A6Y174_VIBCH YFGRGAKQLSYHFNYG
tr|D0H692|D0H692_VIBCH YFGRGAKQLSYHFNYG
tr|C2CD72|C2CD72_VIBCH YFGRGAKQLSYHFNYG
tr|C2IB96|C2IB96_VIBCH YFGRGAKQLSYHFNYG
tr|C9Q8B6|C9Q8B6_9VIBR YFGRGAKQLSYHFNYG
tr|A2PCK1|A2PCK1_VIBCH YFGRGAKQLSYHFNYG
tr|A6AA89|A6AA89_VIBCH YFGRGAKQLSYHFNYG
tr|A5ZZR1|A5ZZR1_VIBCH YFGRGAKQLSYHFNYG

tr|A3EI83|A3EI83_VIBCH	YFGRGAKQLSYHFNYG
tr|C2IPD1|C2IPD1_VIBCH	YFGRGAKQLSYHFNYG
tr|D0IGQ8|D0IGQ8_9VIBR	YFGRGAKQLSYHFNYG
tr|D2YJW8|D2YJW8_VIBMI	YFGRGAKQLSYHFNYG
tr|D2YIE0|D2YIE0_VIBMI	YFGRGAKQLSYHFNYG
tr|D0GSC2|D0GSC2_VIBMI	YFGRGAKQLSYHFNYG
tr|D0HEX5|D0HEX5_VIBMI	YFGRGAKQLSYHFNYG
tr|B8K769|B8K769_VIBPA	YFGRGAKQLSYHFNYG
tr|C9QJ20|C9QJ20_VIBOR	YFGRGAKQLSYHFNYG
tr|C9NQM1|C9NQM1_9VIBR	YFGRGAKQLSYHFNYG
tr|A6AQ45|A6AQ45_VIBHA	YFGRGAKQLSYHFNYG
tr|A7MU32|A7MU32_VIBHB	YFGRGAKQLSYHFNYG
tr|D0XDG9|D0XDG9_VIBHA	YFGRGAKQLSYHFNYG
tr|Q87S05|Q87S05_VIBPA	YFGRGAKQLSYHFNYG
tr|A6B4H6|A6B4H6_VIBPA	YFGRGAKQLSYHFNYG
tr|A7K170|A7K170_VIBSE	YFGRGAKQLSYHFNYG
tr|D0WU94|D0WU94_VIBAL	YFGRGAKQLSYHFNYG
tr|Q1VAD4|Q1VAD4_VIBAL	YFGRGAKQLSYHFNYG
tr|A8T7I7|A8T7I7_9VIBR	YFGRGAKQLSYHFNYG
tr|Q8DF08|Q8DF08_VIBVU	YFGRGAKQLSYHFNYG
tr|Q7MNE0|Q7MNE0_VIBVY	YFGRGAKQLSYHFNYG
tr|B7VJU9|B7VJU9_VIBSL	YFGRGAKQLSYHFNYG
tr|A3XVP4|A3XVP4_9VIBR	YFGRGAKQLSYHFNYG
tr|A3USZ8|A3USZ8_VIBSP	YFGRGAKQLSYHFNYG
tr|A5L6K2|A5L6K2_9GAMM	YFGRGAKQLSYHFNYG
tr|Q25BT4|Q25BT4_VIBPR	YFGRGAKQLSYHFNYG
tr|B5FEC6|B5FEC6_VIBFM	YFGRGAKQLSYHFNYG
tr|Q5E511|Q5E511_VIBF1	YFGRGAKQLSYHFNYG
tr|C9P6B1|C9P6B1_VIBME	YFGRGAKQLSYHFNYG
tr|Q2C9C9|Q2C9C9_9GAMM	YFGRGAKQLSYHFNYG
tr|Q1ZTF9|Q1ZTF9_PHOAS	YFGRGAKQLSYHFNYG
tr|A6FHW3|A6FHW3_9GAMM	YFGRGAKQLSYHFNYG
tr|A4C3H5|A4C3H5_9GAMM	YFGRGAKQLSYHFNYG
tr|Q9WXI9|Q9WXI9_9GAMM	YFGRGSKQLSWNYNFG
tr|Q65ZB0|Q65ZB0_CAEEL	YFGRGAIQISYNYNYG
tr|Q17816|Q17816_CAEEL	YFGRGAIQISYNYNYG
tr|Q95Y59|Q95Y59_CAEEL	YFGRGAIQISYNYNYG
tr|A8X8X1|A8X8X1_CAEBR	YFGRGAIQISYNYNYG
tr|P92013|P92013_CAEEL	YFGRGGIQISYNYNYG
tr|A8XBA9|A8XBA9_CAEBR	YFGRGAIQISYNYNYG
tr|Q962Y5|Q962Y5_ASCSU	YFGRGALQISYNYNYG
tr|A8XUD2|A8XUD2_CAEBR	YFGRGALQLSWNYNYG
tr|O16512|O16512_CAEEL	YFGRGALQLSWNYNYG
tr|B8QUT2|B8QUT2_ZEAMP	YYGRGPLQISWNYNYG
tr|B8QUY7|B8QUY7_ZEAMP	YYGRGPLQISWNYNYG
tr|B8QUX1|B8QUX1_ZEAMP	YYGRGPLQISWNYNYG
tr|B8QUU3|B8QUU3_ZEAMP	YYGRGPLQISWNYNYG
tr|B8QUX8|B8QUX8_ZEAMP	YYGRGPLQISWNYNYG
tr|B8QUS6|B8QUS6_ZEAMP	YYGRGPLQISWNYNYG
tr|B8QUY4|B8QUY4_ZEAMP	YYGRGPLQISWNYNYG
tr|B8QUX5|B8QUX5_ZEAMP	YYGRGPLQISWNYNYG
tr|B8QUT5|B8QUT5_ZEAMP	YYGRGPLQISWNYNYG
tr|B8QUS5|B8QUS5_ZEAMP	YYGRGPLQISWNYNYG
tr|B8QUX2|B8QUX2_ZEAMP	YYGRGPLQISWNYNYG
tr|B8QUX6|B8QUX6_ZEAMP	YYGRGPLQISWNYNYG
tr|B8QUT1|B8QUT1_ZEAMP	YYGRGPLQISWNYNYG
tr|B8QUX9|B8QUX9_ZEAMP	YYGRGPLQISWNYNYG
tr|B8QUY1|B8QUY1_ZEAMP	YYGRGPLQISWNYNYG
tr|B8QUW8|B8QUW8_ZEAMP	YYGRGPLQISWNYNYG
tr|B8QUY6|B8QUY6_ZEAMP	YYGRGPLQISWNYNYG
tr|B8QUS9|B8QUS9_ZEAMP	YYGRGPLQISWNYNYG
tr|B8QUS2|B8QUS2_ZEAMP	YYGRGPLQISWNYNYG
tr|B8QUS3|B8QUS3_ZEAMP	YYGRGPLQISWNYNYG
tr|B8QUT0|B8QUT0_ZEAMP	YYGRGPLQISWNYNYG
tr|B8QUY2|B8QUY2_ZEAMP	YYGRGPLQISWNYNYG
tr|B8QUZ0|B8QUZ0_ZEAMP	YYGRGPLQISWNYNYG
tr|B8QUT7|B8QUT7_ZEAMP	YYGRGPLQISWNYNYG
tr|B8QUS8|B8QUS8_ZEAMP	YYGRGPLQISWNYNYG
tr|B8QUU0|B8QUU0_ZEAMP	YYGRGPLQISWNYNYG
tr|B8QUT9|B8QUT9_ZEAMP	YYGRGPLQISWNYNYG
tr|B8QUZ1|B8QUZ1_ZEAMP	YYGRGPLQISWNYNYG
tr|B8QUZ6|B8QUZ6_ZEAMP	YYGRGPLQISWNYNYG
tr|B8QUU6|B8QUU6_ZEAMP	YYGRGPLQISWNYNYG
tr|B8QUV5|B8QUV5_ZEAMP	YYGRGPLQISWNYNYG
tr|B8QUU9|B8QUU9_ZEAMP	YYGRGPLQISWNYNYG
tr|B8QUV1|B8QUV1_ZEAMP	YYGRGPLQISWNYNYG
tr|B8QUS1|B8QUS1_ZEAMP	YYGRGPLQISWNYNYG

```
tr|Q6JBK8|Q6JBK8_ZEAMP  YYGRGPLQISWNYNYG
tr|B8QUZ2|B8QUZ2_ZEAMP  YYGRGPLQISWNYNYG
tr|B8QUV3|B8QUV3_ZEAMP  YYGRGPLQISWNYNYG
tr|B8QUZ8|B8QUZ8_ZEAMP  YYGRGPLQISWNYNYG
tr|B8QUV0|B8QUV0_ZEAMP  YYGRGPLQISWNYNYG
tr|B8QV00|B8QV00_ZEAMP  YYGRGPLQISWNYNYG
tr|B8QUX4|B8QUX4_ZEAMP  YYGRGPLQISWNYNYG
tr|B8QUT8|B8QUT8_ZEAMP  YYGRGPLQISWNYNYG
tr|B8QUY0|B8QUY0_ZEAMP  YYGRGPLQISWNYNYG
tr|B8QUZ3|B8QUZ3_ZEAMP  YYGRGPLQISWNYNYG
tr|B8QUY5|B8QUY5_ZEAMP  YYGRGPLQISWNYNYG
tr|B8QV01|B8QV01_ZEAMP  YYGRGPLQISWNYNYG
tr|B8QUX0|B8QUX0_ZEAMP  YYGRGPLQISWNYNYG
tr|B8QUY3|B8QUY3_ZEAMP  YYGRGPLQISWNYNYG
tr|Q6JBK4|Q6JBK4_ZEAMP  YYGRGPLQISWNYNYG
tr|B6TEL0|B6TEL0_MAIZE  YYGRGPLQISWNYNYG
tr|B8QUV9|B8QUV9_ZEAMP  YYGRGPLQISWNYNYG
tr|B8QUW0|B8QUW0_ZEAMP  YYGRGPLQISWNYNYG
tr|B8QUW9|B8QUW9_ZEAMP  YYGRGPLQISWNYNYG
tr|B8QUW4|B8QUW4_ZEAMP  YYGRGPLQISWNYNYG
tr|D0EM57|D0EM57_MAIZE  YYGRGPLQISWNYNYG
tr|Q6JBK6|Q6JBK6_ZEAMP  YYGRGPLQISWNYNYG
tr|Q6JBL1|Q6JBL1_ZEAMP  YYGRGPLQISWNYNYG
tr|Q6JBL0|Q6JBL0_ZEAMP  YYGRGPLQISWNYNYG
tr|Q6JBK7|Q6JBK7_ZEAMP  YYGRGPLQISWNYNYG
tr|Q6JBL4|Q6JBL4_ZEAMP  YYGRGPLQISWNYNYG
tr|Q6JBK9|Q6JBK9_ZEAMP  YYGRGPLQISWNYNYG
tr|Q6JBK1|Q6JBK1_ZEADI  YYGRGPLQISWNYNYG
tr|Q6JBK0|Q6JBK0_ZEADI  YYGRGPLQISWNYNYG
tr|Q6JBL5|Q6JBL5_ZEAMP  YYGRGPLQISWNYNYG
tr|Q6JBL3|Q6JBL3_ZEAMP  YYGRGPLQISWNYNYG
tr|B4FTS6|B4FTS6_MAIZE  YYGRGPLQISWNYNYG
tr|Q6JBL2|Q6JBL2_ZEAMP  YYGRGPLQISWNYNYG
tr|Q6JBK3|Q6JBK3_ZEAMP  YYGRGPLQISWNYNYG
tr|Q6JBL6|Q6JBL6_TRIDA  YYGRGPLQISWNYNYG
tr|A9XB16|A9XB16_SACOF  YYGRGPLQISWNYNYG
tr|A9XAV3|A9XAV3_SACOF  YYGRGPLQISWNYNYG
tr|A9XB14|A9XB14_SACOF  YYGRGPLQISWNYNYG
tr|Q94EL5|Q94EL5_SACOF  YYGRGPLQISWNYNYG
tr|Q3HWG5|Q3HWG5_SORHL  YHGRGPLQISWNYNYG
tr|Q94EL3|Q94EL3_SORHL  YYGRGPLQISWNYNYG
tr|Q3HWG6|Q3HWG6_SORHL  YYGRGPLQISWNYNYG
tr|Q3HWG7|Q3HWG7_SORHL  YYGRGPLQISWNYNYG
tr|C5YBE8|C5YBE8_SORBI  YYGRGPLQISWNYNYG
tr|C5YBE9|C5YBE9_SORBI  YYGRGPLQISWNYNYG
tr|Q93WT2|Q93WT2_SORBI  YYGRGPLQISWNYNYG
tr|Q93WT1|Q93WT1_SORBI  YYGRGPLQISWNYNYG
tr|Q94EL4|Q94EL4_SORBI  YYGRGPLQISWNYNYG
tr|Q941I5|Q941I5_SORBI  YYGRGPLKISWDYNYG
tr|Q6JBQ2|Q6JBQ2_ZEAMP  YYGRGPLQISWNYNYG
tr|B8QV71|B8QV71_ZEAMP  YYGRGPLQISWNYNYG
tr|B8QV49|B8QV49_ZEAMP  YYGRGPLQISWNYNYG
tr|B8QV22|B8QV22_ZEAMP  YYGRGPLQISWNYNYG
tr|B8QV52|B8QV52_ZEAMP  YYGRGPLQISWNYNYG
tr|Q6JBQ3|Q6JBQ3_ZEAMP  YYGRGPLQISWNYNYG
tr|Q6JBQ5|Q6JBQ5_ZEAMP  YYGRGPLQISWNYNYG
tr|Q6JBQ7|Q6JBQ7_ZEAMP  YYGRGPLQISWNYNYG
tr|Q6JBQ1|Q6JBQ1_ZEAMP  YYGRGPLQISWNYNYG
tr|Q6JBQ9|Q6JBQ9_ZEAMP  YYGRGPLQISWNYNYG
tr|B8QV62|B8QV62_ZEAMP  YYGRGPLQISWNYNYG
tr|B8QV66|B8QV66_ZEAMP  YYGRGPLQISWNYNYG
tr|B8QV48|B8QV48_ZEAMP  YYGRGPLQISWNYNYG
tr|B8QV63|B8QV63_ZEAMP  YYGRGPLQISWNYNYG
tr|B8QV29|B8QV29_ZEAMP  YYGRGPLQISWNYNYG
tr|B8QV69|B8QV69_ZEAMP  YYGRGPLQISWNYNYG
tr|B8QV57|B8QV57_ZEAMP  YYGRGPLQISWNYNYG
tr|Q6JBP2|Q6JBP2_ZEADI  YYGRGPLQISWNYNYG
tr|B8QV61|B8QV61_ZEAMP  YYGRGPLQISWNYNYG
tr|B8QV56|B8QV56_ZEAMP  YYGRGPLQISWNYNYG
tr|Q6JBQ6|Q6JBQ6_ZEAMP  YYGRGPLQISWNYNYG
tr|B8QV26|B8QV26_ZEAMP  YYGRGPLQISWNYNYG
tr|B8QV03|B8QV03_ZEAMP  YYGRGPLQISWNYNYG
tr|B8QV04|B8QV04_ZEAMP  YYGRGPLQISWNYNYG
tr|B8QV08|B8QV08_ZEAMP  YYGRGPLQISWNYNYG
tr|B8QV41|B8QV41_ZEAMP  YYGRGPLQISWNYNYG
tr|B8QV06|B8QV06_ZEAMP  YYGRGPLQISWNYNYG
tr|B8QV12|B8QV12_ZEAMP  YYGRGPLQISWNYNYG
```

tr|B8QV09|B8QV09_ZEAMP	YYGRGPLQISWNYNYG
tr|B8QV53|B8QV53_ZEAMP	YYGRGPLQISWNYNYG
tr|B8QV35|B8QV35_ZEAMP	YYGRGPLQISWNYNYG
tr|C0P451|C0P451_MAIZE	YYGRGPLQISWNYNYG
tr|Q6JBP8|Q6JBP8_ZEAMP	YYGRGPLQISWNYNYG
tr|B8QV28|B8QV28_ZEAMP	YYGRGPLQISWNYNYG
tr|Q6JBP7|Q6JBP7_ZEADI	YYGRGPLQISWNYNYG
tr|Q6JBQ4|Q6JBQ4_ZEAMP	YYGRGPLQISWNYNYG
tr|Q6JBQ0|Q6JBQ0_ZEAMP	YYGRGPLQISWNYNYG
tr|B8QV18|B8QV18_ZEAMP	YYGRGPLQISWNYNYG
tr|B8QV65|B8QV65_ZEAMP	YYGRGPLQISWNYNYG
tr|B8QV14|B8QV14_ZEAMP	YYGRGPLQISWNYNYG
tr|B8QV47|B8QV47_ZEAMP	YYGRGPLQISWNYNYG
tr|B8QV74|B8QV74_ZEAMP	YYGRGPLQISWNYNYG
tr|B8QV19|B8QV19_ZEAMP	YYGRGPLQISWNYNYG
tr|Q6JBP9|Q6JBP9_ZEAMP	YYGRGPLQISWNYNYG
tr|Q6JBP5|Q6JBP5_ZEADI	YYGRGPLQISWNYNYG
tr|B8QV58|B8QV58_ZEAMP	YYGRGPLQISWNYNYG
tr|B8QV68|B8QV68_ZEAMP	YYGRGPLQISWNYNYG
tr|B8QV73|B8QV73_ZEAMP	YYGRGPLQISWNYNYG
tr|B8QV67|B8QV67_ZEAMP	YYGRGPLQISWNYNYG
tr|Q6JBQ8|Q6JBQ8_ZEAMP	YYGRGPLQISWNYNYG
tr|B8QV42|B8QV42_ZEAMP	YYGRGPLQISWNYNYG
tr|B8QV46|B8QV46_ZEAMP	YYGRGPLQISWNYNYG
tr|Q6JBR1|Q6JBR1_ZEAMP	YYGRGPLQISWNYNYG
tr|B8QV34|B8QV34_ZEAMP	YYGRGPLQISWNYNYG
tr|B8QV37|B8QV37_ZEAMP	YYGRGPLQISWNYNYG
tr|Q6JBP3|Q6JBP3_ZEADI	YYGRGPLQISWNYNYG
tr|B8QV24|B8QV24_ZEAMP	YYGRGPLQISWNYNYG
tr|B8QV05|B8QV05_ZEAMP	YYGRGPLQISWNYNYG
tr|B8QV64|B8QV64_ZEAMP	YYGRGPLQISWNYNYG
tr|B8QV32|B8QV32_ZEAMP	YYGRGPLQISWNYNYG
tr|Q6JBR2|Q6JBR2_TRIDA	YYGRGPLQISWNYNYG
tr|C5YBF0|C5YBF0_SORBI	YYGRGPLQISWNYNYG
tr|C5XJT5|C5XJT5_SORBI	YYGRGPLQLSWNYNYG
tr|C0PKN5|C0PKN5_MAIZE	YYGRGPLQVSWNYNYG
tr|C5YBF1|C5YBF1_SORBI	YYGRGPLQISWNYNYG
tr|C5YBE7|C5YBE7_SORBI	YYGRGPLQISWNYNYG
tr|C5YBE6|C5YBE6_SORBI	YYGRGPLQISWNFNYG
tr|B6SZA3|B6SZA3_MAIZE	YYGRGPLQISWNFNYG
tr|B8ARC6|B8ARC6_ORYSI	YYGRGPLQISWNYNYG
tr|A0B9Y6|A0B9Y6_ORYSA	YYGRGPLQISWNYNYG
tr|C6F3C7|C6F3C7_ORYGR	YYGRGPLQISWNYNYG
tr|Q7GC32|Q7GC32_ORYSI	YYGRGPLQISWNFNYG
tr|Q25AB8|Q25AB8_ORYSA	YYGRGPLQISWNFNYG
tr|C5XX83|C5XX83_SORBI	YYGRGPLQISWNYNYG
tr|B6TT00|B6TT00_MAIZE	YYGRGPLQISWNFNYG
tr|A2X6Z9|A2X6Z9_ORYSI	YYGRGPLQISWNFNYG
tr|D1MJ99|D1MJ99_9ROSI	YYGRGPLQLTWNYNYG
tr|O82547|O82547_CITSI	YYGRGPIQLTGNGNYG
tr|A7Y7L3|A7Y7L3_PRUDU	-YGRGPLQLTWNYNYG
tr|Q9XEN3|Q9XEN3_WHEAT	YYGRGPLQLTWNYNYG
tr|Q9XEN6|Q9XEN6_WHEAT	YYGRGPLQLSWNYNYG
tr|Q7M1Q9|Q7M1Q9_PHYAM	YYGRGPLQLSWNYNYG
tr|C1K2E8|C1K2E8_ELAOL	YFGRGPLQLSWNYNYG
tr|A0A3A1|A0A3A1_ARTAN	YYGRGPIQLSWNFNYG
tr|D0QU15|D0QU15_LACSA	YYGRGPIQLSWNYNYG
tr|B9IQS7|B9IQS7_POPTR	YYGRGPIQLSWNYNYG
tr|B9N605|B9N605_POPTR	YYGRGPIQLSWNFNYG
tr|B9VQ34|B9VQ34_PYRPY	YYGRGPIQLSWNFNYG
tr|O23248|O23248_ARATH	YYGRGPIQLSWNFNYG
tr|Q9M2U5|Q9M2U5_ARATH	YYGRGPIQLSWNFNYG
tr|D1HU25|D1HU25_VITVI	YYGRGPIQLSWNFNYG
tr|A5BSD0|A5BSD0_VITVI	YYGRGPIQLSWNFNYG
tr|C6TK83|C6TK83_SOYBN	YYGRGPIQLTWNFNYG
tr|Q43686|Q43686_VIGUN	YT-VVPIQLSWNFNYG
tr|C6T397|C6T397_SOYBN	YYGRGPIQLSWNFNYG
tr|C6T7J9|C6T7J9_SOYBN	YYGRGPIQLSWNFNYG
tr|Q6RV28|Q6RV28_MEDTR	YYGRGPIQLSWNYNYG
tr|B8Y647|B8Y647_MEDSA	YYGRGPIQLSWNYNYG
tr|A1IJ67|A1IJ67_TOBAC	YYGRGPIQLSWNFNYG
tr|Q96409|Q96409_DAUCA	YFGRGPLQLTWNYNYI
tr|Q7XAF6|Q7XAF6_DAUCA	YFGRGPLQLTWNYNYI
tr|Q96408|Q96408_DAUCA	YFGRGPLQLTWNYNYI
tr|Q96411|Q96411_DAUCA	YFGRGPLQLTWNYNYI
tr|Q96410|Q96410_DAUCA	YFGRGPLQLTWNYNYI
tr|B9T8H9|B9T8H9_RICCO	YYGRGPIQLTWNYNYG

```
tr|D1HU30|D1HU30_VITVI  YYGRGPLQISWNYNYG
tr|A5C2S9|A5C2S9_VITVI  YYGRGPLQISWNYNYG
tr|D1HU29|D1HU29_VITVI  YYGRGPIQISWNYNYG
tr|A5BSD2|A5BSD2_VITVI  YYGRGPIQLSWNYNYG
tr|D1HU35|D1HU35_VITVI  YYGRGPIQLSWNYNYG
tr|A5AJB3|A5AJB3_VITVI  YYGRGPIQLSWNYNYG
tr|Q7XB39|Q7XB39_VITVI  YYGRGPIQLSWNYNYG
tr|A5BKN8|A5BKN8_VITVI  YYGRGPIQLSWNYNYG
tr|D1HU23|D1HU23_VITVI  YYGRGPIQLSWNYNYG
tr|A5AS02|A5AS02_VITVI  YYGRGPIQLSWNYNYG
tr|D1HU28|D1HU28_VITVI  YYGRGPLQITWNFNYG
tr|A5BCX7|A5BCX7_VITVI  YYGRGPLQITWNFNYG
tr|A5BSD1|A5BSD1_VITVI  YYGRGPIQISWNYNYG
tr|D1HU27|D1HU27_VITVI  YYGRGPIQLSWNYNYG
tr|Q710B9|Q710B9_ALNGL  YYGRGPFQLSWNYNYG
tr|D1HU32|D1HU32_VITVI  YYGRGPIQITWNYNYG
tr|B9IQS9|B9IQS9_POPTR  YYGRGPLQLSWNYNYG
tr|A9PH35|A9PH35_POPTR  YYGRGPLQLSWNYNYG
tr|B9IQT0|B9IQT0_POPTR  YHGRGPIQLSWNYNYG
tr|B9N604|B9N604_POPTR  YHGRGPLQISWNFNYG
tr|B9IQS8|B9IQS8_POPTR  YFGRGPLQLTWNYNYG
tr|B9PF44|B9PF44_POPTR  YFGRGPLQLTWNYNYG
tr|B9SX44|B9SX44_RICCO  YFGRGPIQLTWNYNYG
tr|Q7X9F8|Q7X9F8_9FABA  YYGRGPLQITWNYNYG
tr|Q43150|Q43150_SAMNI  YFGRGPLQLSWNYNYG
tr|Q43151|Q43151_SAMNI  YHGRGPLQLSWNYNYG
tr|O23804|O23804_9CARY  YYGRGPIQITWNYNYG
tr|O23803|O23803_9CARY  YYGRGPIQITWNYNYG
tr|O23806|O23806_9CARY  YYGRGPIQITWNYNYG
tr|O23805|O23805_9CARY  YYGRGPLQLTWNYNYG
tr|Q9XFW7|Q9XFW7_BETVU  YYGRGPLQITWNYNYI
tr|A9ZMK1|A9ZMK1_NEPAL  YYGRGPIQISWNYNYG
tr|B0FZ27|B0FZ27_9ROSI  YYGRGPLQLTWNYNYG
tr|Q7XAU6|Q7XAU6_VITVI  YYGRGPLQLTWNYNYG
tr|O24530|O24530_VITVI  YYGRGPLQLTWNYNYG
tr|B0FZ26|B0FZ26_9ROSI  YYGRGPLQLTWNYNYG
tr|O24531|O24531_VITVI  YYGRGPLQLTWNYNYG
tr|A5AK36|A5AK36_VITVI  YYGRGPLQLTWNYNYG
tr|D1HU34|D1HU34_VITVI  YYGRGPLQISWNYNYG
tr|Q3HUM0|Q3HUM0_LINUS  YYGRGPLQLTWNYNYQ
tr|Q852P9|Q852P9_9LILI  YFGRGPMQLSWNYNYI
tr|Q94C47|Q94C47_BRANA  YYGRGAIQLSWNYNYG
tr|Q43391|Q43391_BRANA  YYGRGAIQLSWNYNYG
tr|A5JVZ1|A5JVZ1_BRAJU  YYGRGAIQLSWNYNYG
tr|O24603|O24603_ARATH  YYGRGAIQLSWNYNYG
tr|O04881|O04881_LINUS  YYGRGPLQLTWNYNYQ
tr|C0PRG2|C0PRG2_PICSI  YHGRGPLQLSWNYNYG
tr|A9NSX6|A9NSX6_PICSI  YHGRGPLQLSWNYNYG
tr|A9NP56|A9NP56_PICSI  YHGRGPLQLSWNYNYG
tr|A9NQV7|A9NQV7_PICSI  YHGRGPLQLSWNYNYG
tr|Q4VSV1|Q4VSV1_PICAB  YHGRGPLQISWNYNYG
tr|C3VP99|C3VP99_PSEMZ  YHGRGPLQLSWNYNYG
tr|C3VPA0|C3VPA0_PSEMZ  YHGRGPLQLSWNYNYG
tr|Q6WSR8|Q6WSR8_PICAB  YHGRGPLQLSWNYNYG
tr|C0JR29|C0JR29_PICAB  YHGRGPLQLSWNYNCG
tr|C0JR28|C0JR28_PICAB  YHGRGPLQLSWNYNYG
tr|Q40838|Q40838_PICGL  YHGRGPLQLSWNYNYG
tr|A9P2F9|A9P2F9_PICSI  YHGRGPLQLSWNYNYG
tr|Q6WSR9|Q6WSR9_PICAB  YHGRGPLQLSWNYNYG
tr|Q6WSS1|Q6WSS1_PICAB  YHGRGPLQLSWNYNYG
tr|Q6E6N0|Q6E6N0_PICAB  YHGRGPLQLSWNYNYG
tr|A9NLX3|A9NLX3_PICSI  YHGRGPLQLSWNYNYG
tr|Q6WSS0|Q6WSS0_PICAB  YHGRGPLQLSWNYNYG
tr|B8LPT4|B8LPT4_PICSI  YHGRGPLQLSWNYNYG
tr|Q596H9|Q596H9_PINMO  YHGRGPLQLSWNYNYG
tr|Q596H8|Q596H8_PINMO  YHGRGPLQLSWNYNYG
tr|Q596H7|Q596H7_PINMO  YHGRGPLQLSWNYNYG
tr|B8LQM1|B8LQM1_PICSI  YHGRGPLQLSWNYNYG
tr|Q5NTA4|Q5NTA4_CRYJA  YHGRGPLQLSWNYNYG
tr|A9NRW0|A9NRW0_PICSI  YYGRGPLQLTGNGNYG
tr|A9NM89|A9NM89_PICSI  YYGRGPLQLTGNGNYG
tr|A9NM41|A9NM41_PICSI  YYGRGPLQLTGNANYG
tr|A9NTE7|A9NTE7_PICSI  YYGRGPLQLTGNANYG
tr|A9NPI4|A9NPI4_PICSI  YYGRGPLQLTGNANYG
tr|C0PQ29|C0PQ29_PICSI  YYGRGPLQLTGNANYG
tr|B8LNR4|B8LNR4_PICSI  YYGRGPLQLTGNANYG
tr|A9NP79|A9NP79_PICSI  YYGRGPLQLTWNYNYG
```

```
tr|A9NT56|A9NT56_PICSI  YYGRGPLQLKWNYNYG
tr|B7X6W0|B7X6W0_BRARC  YFGRGPIQLSWNYNYG
tr|Q05K38|Q05K38_BRARP  YFGRGPIQLSWNYNYG
tr|A8IXF7|A8IXF7_BRACM  YFGRGPIQLSWNYNYG
tr|Q9AUF7|Q9AUF7_BRANA  YFGRGPIQLSWNYNYG
tr|Q9AUF4|Q9AUF4_BRACM  YFGRGPIQLSWNYNYG
tr|Q9AUF6|Q9AUF6_BRANA  YFGRGPIQLSWNYNYG
tr|Q9AUF5|Q9AUF5_BRAOL  YFGRGPIQLSWNYNYG
tr|O24658|O24658_ARATH  YFGRGPIQLSWNYNYG
tr|O24598|O24598_ARATH  YHGRGPLLLSWNFNYG
tr|O22841|O22841_ARATH  YYGRGPIQITWNYNYG
tr|O22842|O22842_ARATH  YYGRGPIQITWNYNYG
tr|O24654|O24654_ARATH  YYGRGLLQSITWNEYY
tr|Q9FXB8|Q9FXB8_ARATH  YYGRGLLQSIKWNEFY
tr|A9RL26|A9RL26_PHYPA  YYSRGPLQLSWNYNYK
tr|Q4A3V2|Q4A3V2_PHYPA  YYGRGPIQLSWNYNYG
tr|A9T8R4|A9T8R4_PHYPA  YYGRGPIQLSWNYNYG
tr|A9ZSX9|A9ZSX9_9BRYO  YYGRGPIQLSWNYNYG
tr|A9TQE8|A9TQE8_PHYPA  YFGRGPIQLSWNYNYG
tr|A9S7X6|A9S7X6_PHYPA  YFGRGPIQLFWNYNYG
tr|A9T7E8|A9T7E8_PHYPA  YFGRGPLQISWNYNYG
tr|A9SXW7|A9SXW7_PHYPA  YFGRGPLQLSWNFNYG
tr|A9TSW7|A9TSW7_PHYPA  YNGRGPLQLSWNYNYL
tr|A9AX07|A9AX07_HERA2  YFGRGPIQLSWNYNYG
tr|B3PB24|B3PB24_CELJU  YYGRGPIQLSWNGNYC
tr|A4BFB3|A4BFB3_9GAMM  YYGRGPIQLSWNGNYC
tr|Q08TW9|Q08TW9_STIAU  YYGRGPIQLSWNGNYC
tr|Q08MN3|Q08MN3_STIAU  YYGRGPIQLSWNGNYC
tr|Q1D3S1|Q1D3S1_MYXXD  YYGRGPIQLSWNGNYC
tr|Q59I46|Q59I46_BACCI  YYGRGPIQLSWNYNYG
tr|D3BFR3|D3BFR3_POLPA  YYGRGPLQLSWNYNYN
tr|D3BIG8|D3BIG8_POLPA  YYGRGPIQLSWNYNYA
tr|Q9LBM0|Q9LBM0_BURGA  YYGRGPMQLSWDYNYK
tr|C7PZK0|C7PZK0_CATAD  YYGRGPLQLSWNFNYY
tr|C7Q712|C7Q712_CATAD  YYGRGPLQLSWNFNYY
tr|Q9RHK5|Q9RHK5_9ACTO  YYGRGPVQLSWNFNYK
tr|Q9RHK6|Q9RHK6_STRLI  YYGRGPVQLSWNFNYK
tr|Q8CK55|Q8CK55_STRCO  YYGRGPVQLSWNFNYK
tr|Q9Z9M4|Q9Z9M4_STRCO  YYGRGPVQLSWNFNYK
tr|B8XA70|B8XA70_9ACTO  YYGRGPVQLSWNFNYK
tr|Q9RHK1|Q9RHK1_9ACTO  YYGRGPVQLSWNFNYK
tr|A3KIM3|A3KIM3_STRAM  YYGRGPVQLSWNFNYK
tr|D1XV00|D1XV00_9ACTO  YYGRGPIQLSWNFNYK
tr|Q9RHU5|Q9RHU5_STRTL  YYGRGPIQLSWNYNYK
tr|Q9RHU4|Q9RHU4_STRTL  YYGRGPIQLSWNFNYK
tr|Q75UW8|Q75UW8_9ACTO  YYGRGPLQLSWNFNYK
tr|Q9X5U3|Q9X5U3_STRLA  YYGRGPLQISWNFNYK
tr|B5HZA0|B5HZA0_9ACTO  YYGRGPIQLSWNFNYK
tr|Q9RHK0|Q9RHK0_STRSQ  YYGRGPIQLSWNFNYK
tr|Q9RHK9|Q9RHK9_STRCO  YYGRGPIQLSWNFNYK
tr|Q9RHK8|Q9RHK8_STRLI  YYGRGPIQLSWNFNYK
tr|Q9Z9M6|Q9Z9M6_STRCO  YYGRGPIQLSWNFNYK
tr|Q9S6T0|Q9S6T0_STRCO  YYGRGPIQLSWNFNYK
tr|C6KLH5|C6KLH5_9ACTO  YYGRGPIQLSWNFNYK
tr|Q9RHK7|Q9RHK7_STRLI  YYGRGPIQLSWNFNYK
tr|A3KJH0|A3KJH0_STRAM  YYGRGPIQLSWNFNYK
tr|Q9RHJ9|Q9RHJ9_STRSQ  YYGRGPIQLSWNFNYK
tr|Q9RHK2|Q9RHK2_9ACTO  YYGRGPIQLSWNFNYK
tr|B3XZQ2|B3XZQ2_STRCN  YYGRGPIQLSWNFNYK
tr|Q9RHJ7|Q9RHJ7_STRSQ  YYGRGPIQLSWNFNYK
tr|Q9Z4P2|Q9Z4P2_STROI  YYGRGPIQLSWNFNYK
tr|C9Z1I5|C9Z1I5_STRSW  YYGRGPIQLSWNFNYK
tr|Q75UW9|Q75UW9_9ACTO  YYGRGPIQLSWNFNYK
tr|Q75UX3|Q75UX3_9ACTO  YYGRGPIQLSWNFNYK
tr|D2AUP6|D2AUP6_STRRD  YYGRGPIQLSWNFNYK
tr|C6W984|C6W984_ACTMD  YYGRGPIQLSWNFNYK
tr|Q75UX4|Q75UX4_AMYOR  YYGRGPIQLSWNFNYK
tr|Q75UW4|Q75UW4_9ACTO  YYGRGPVQLSWNFNYK
tr|Q75UW6|Q75UW6_PLARO  YYGRGPIQLSWNFNYK
tr|D1XKV1|D1XKV1_9ACTO  YYGRGPIQLSWNFNYK
tr|Q9RHK3|Q9RHK3_9ACTO  YYGRGPIQLSWNFNYK
tr|C9NH04|C9NH04_9ACTO  YYGRGPIQLSWNFNYK
tr|B8XA68|B8XA68_9ACTO  YYGRGPIQLSWNFNYK
tr|Q75UW2|Q75UW2_9MICO  YYGRGPVQLSWNFNYK
tr|Q75UX1|Q75UX1_KIBAR  YYGRGPIQLSWNFNYK
tr|Q75UW7|Q75UW7_9ACTO  YYGRGPIQLSWNFNYK
tr|Q75UW1|Q75UW1_STRHI  YYGRGPIQLSWNFNYK
```

```
tr|Q75UX0|Q75UX0_KIBAR  YYGRGPIQLSWNFNYK
tr|D2Q3H4|D2Q3H4_9ACTO  YYGRGPIQLSWNFNYK
tr|A4XD26|A4XD26_SALTO  YYGRGPIQLSWNFNYN
tr|Q75UX2|Q75UX2_9ACTO  YYGRGPIQLSWNFNYN
tr|B5GNL7|B5GNL7_STRCL  YYGRGPIQLSWNFNYK
tr|C4DHS0|C4DHS0_9ACTO  YYGRGPIQ-SWNFNYK
tr|Q764N6|Q764N6_9ACTO  YYGRGPIQLSWNFNYK
tr|Q9RHJ8|Q9RHJ8_STRSQ  YYGRGPIQLSWNFNYK
tr|Q845Z9|Q845Z9_9ACTO  YYGRGPIQLSWNFNYK
tr|Q5J1K1|Q5J1K1_9ACTO  YYGRGPIQLSWNFNYK
tr|B8XA67|B8XA67_9ACTO  YYGRGPIQLSWNFNYK
tr|Q75UX7|Q75UX7_9PSEU  YYGRGPIQLSWNYNYK
tr|Q6V8K4|Q6V8K4_9ACTO  YYGKGPIQLSWNFNYK
tr|Q58GF8|Q58GF8_STRLN  YYGKGPIQLSWNFNYK
tr|B8XA66|B8XA66_9ACTO  YYGKGPIQLSWNFNYK
tr|B8XA65|B8XA65_9ACTO  YYGKGPIQLSWNFNYK
tr|B8XA69|B8XA69_9ACTO  YYGKGPIQLSWNFNYK
tr|B8XA59|B8XA59_9ACTO  YYGKGPIQLSWNFNYK
tr|B8XA61|B8XA61_9ACTO  YYGRGPIQLSWNFNYK
tr|B8XA60|B8XA60_9ACTO  YYGRGPIQLSWNFNYK
tr|B8XA62|B8XA62_9ACTO  YYGRGPIQLSWNFNYK
tr|B8XA58|B8XA58_9ACTO  YYGRGPIQLSWNFNYR
tr|B8XA63|B8XA63_9ACTO  YYGRGPIQLSWNFNYK
tr|C1YRN0|C1YRN0_NOCDA  YYGRGPIQLSWNYNYK
tr|D1WRR0|D1WRR0_9ACTO  YYGRGPIQLSWNFNYK
tr|B1VMB9|B1VMB9_STRGG  YYGRGPIQLSWNFNYK
tr|O50152|O50152_STRGR  YYGRGPIQLSWNFNYK
tr|Q8GI53|Q8GI53_9ACTO  YYGKGPIQLAWNYNYK
tr|Q75UW3|Q75UW3_9ACTO  YYGRGPIQLSWNYNYK
tr|Q75UW5|Q75UW5_PLARO  YYGRGPIQPSWNYNYK
tr|D2BDW7|D2BDW7_STRRD  YYGRGPIQLSWNFNYK
tr|Q75UX6|Q75UX6_9ACTO  YHGRGPIQLSWNFNYK
tr|B5GNL8|B5GNL8_STRCL  YYGRGPIQLSWNFNYK
tr|B1VMC0|B1VMC0_STRGG  YYGRGPIMFSWNFNYK
tr|D1WRQ9|D1WRQ9_9ACTO  YYGRGPIMFSWNFNYK
tr|D1XKV2|D1XKV2_9ACTO  YYGRGPIMFSWNFNYK
tr|B8XA64|B8XA64_9ACTO  YYGRGPIQLSWNFNYK
tr|Q75UW0|Q75UW0_CELCE  YYGRGPLQLSWNFNYR
tr|A9WKV3|A9WKV3_RENSM  YYGRGSLQLSWNYNYK
tr|A3Q1W7|A3Q1W7_MYCSJ  YFGRGPGQLSWNYNYR
tr|A1UIF3|A1UIF3_MYCSK  YFGRGPGQLSWNYNYR
tr|Q1B6M2|Q1B6M2_MYCSS  YFGRGPGQLSWNYNYR
tr|Q8STP5|Q8STP5_ENCCU  YHGRGFIQLSWPDNYK
tr|B7XK35|B7XK35_ENTBH  YYGRGFIQLSWPANYK
tr|B0XK59|B0XK59_CULQU  YYGRGYIQLTWDYNYR
tr|B0XJ44|B0XJ44_CULQU  YYGRGYIQLTWDYNYR
tr|Q16JP0|Q16JP0_AEDAE  YYGRGFIQLTHDYNYR
tr|B0WBS6|B0WBS6_CULQU  YYGRGYIQLTWDYNYR
tr|D2VI28|D2VI28_NAEGR  YKGRGPIQITGRANYR
tr|D2VMQ6|D2VMQ6_NAEGR  YKGRGPIQITGRSNYR
tr|D2VBA0|D2VBA0_NAEGR  YKGRGPIQITGRANYR
tr|D2VS08|D2VS08_NAEGR  YKGRGPIQITGRANYR
tr|Q08VR7|Q08VR7_STIAU  FKGRGPIQLTGRSNYR
tr|Q1CVS9|Q1CVS9_MYXXD  YKGRGPIQLTGRANYR
tr|C6BBQ6|C6BBQ6_RALP1  FMGRGLIQITGRANYA
tr|B3R5D7|B3R5D7_CUPTR  FRGRGLIQITGRANYA
tr|B4EG73|B4EG73_BURCJ  YRGGGLLQITGRYNFR
tr|B2JCY6|B2JCY6_BURP8  FCGRGCIQVTGRSNYA
tr|Q48J35|Q48J35_PSE14  YRGRGLIQITGRANYA
tr|Q4ZSR6|Q4ZSR6_PSEU2  YRGRGLIQITGRANYA
tr|Q87Y72|Q87Y72_PSESM  YRGRGLIQITGRANYA
tr|Q877M3|Q877M3_PSESM  YRGRGLIQITGRANYA
tr|C3JZM4|C3JZM4_PSEFS  YRGRGLLQITGRANYA
tr|Q48KG4|Q48KG4_PSE14  YRGRGLIQITGRANYA
tr|Q87ZN5|Q87ZN5_PSESM  YRGRGLIQVTGRDNYA
tr|Q48JX1|Q48JX1_PSE14  YRGRGLIQVTGRANYE
tr|Q4KF46|Q4KF46_PSEF5  YRGRGLIQVTGRANYE
tr|Q4ZMK9|Q4ZMK9_PSEU2  YRGRGLIQVTGRTNYE
tr|Q48NQ6|Q48NQ6_PSE14  YRGRGLIQVTGRANYE
tr|B0KT09|B0KT09_PSEPG  YRGRGLIQITGRDNYE
tr|B9YYW2|B9YYW2_9NEIS  YCGRGLIQITGRRNYE
tr|Q0EWC5|Q0EWC5_9PROT  YRGRGLIMVTGHDNYK
tr|B1WPV6|B1WPV6_CYAA5  FKGRGLIQLTGRANYR
tr|C4S3X5|C4S3X5_YERBE  FRGRGLKQITFLDNYY
tr|B1JI29|B1JI29_YERPY  FRGRGLKQITFLDNYY
tr|C9XYF8|C9XYF8_CROTZ  YRGRGLKQITFKANYE
tr|Q858F0|Q858F0_9CAUD  YRGRGLIQITGLNNYR
```

```
tr|B7UGP7|B7UGP7_ECO27  YRGRGLIQITGLNNYR
tr|B7NQQ3|B7NQQ3_ECO7I  YRGRGLIQITGLNNYR
tr|A1AE01|A1AE01_ECOK1  YRGRGLIQITGLNNYR
tr|Q286Y9|Q286Y9_9CAUD  YRGRGLIQITGLNNYR
tr|A6T8B6|A6T8B6_KLEP7  YRGRGLIQTTGLNNYR
tr|D0YLN0|D0YLN0_KLEVA  YRGRGLIQTTGLNNYR
tr|C4X6X0|C4X6X0_KLEPN  YRGRGLIQTTGLNNYR
tr|C8SZ03|C8SZ03_KLEPR  YRGRGLIQTTGLNNYR
tr|B5NGY0|B5NGY0_SALET  YRGRGLIQITGLNNYR
tr|B5MHX8|B5MHX8_SALET  YRGRGLIQITGLNNYR
tr|A9MSG1|A9MSG1_SALPB  YRGRGLIQITGLNNYR
tr|B5Q9Z2|B5Q9Z2_SALVI  YRGRGLIQITGLNNYR
tr|C8Q937|C8Q937_9ENTR  YRGRGIIQITGLSNYR
tr|A7MLQ6|A7MLQ6_ENTS8  YRGRGLIHITGLENYR
tr|C9Y1P6|C9Y1P6_CROTZ  YRGRGLIQITGLENYR
tr|B5QY63|B5QY63_SALEP  YRGRGLLQITGRENYV
tr|C1M3H7|C1M3H7_9ENTR  YRGRGLLQITGRENYA
tr|A9N3F1|A9N3F1_SALPB  YRGRGLIQITGLHNYR
tr|B4TRD8|B4TRD8_SALSV  YRGRGLIQITGLENYT
tr|B5CDL1|B5CDL1_SALET  YRGRGLIQITGLENYT
tr|Q8Z7W2|Q8Z7W2_SALTI  YRGRGLIQITGLENYT
tr|A9N4H1|A9N4H1_SALPB  YRGRGLIQITGLENYT
tr|Q8ZQH4|Q8ZQH4_SALTY  YRGRGLIQITGLENYT
tr|Q57Q94|Q57Q94_SALCH  YRGRGLIQITGLENYT
tr|B5PLD3|B5PLD3_SALET  YRGRGLIQITGLENYT
tr|B5FQV2|B5FQV2_SALDC  YRGRGLIQITGLENYT
tr|B6S2Y3|B6S2Y3_SALDU  YRGRGLIQITGLENYT
tr|D0ZLG3|D0ZLG3_SALT1  YRGRGLIQITGLENYT
tr|C9X7P3|C9X7P3_SALTD  YRGRGLIQITGLENYT
tr|B5N7J2|B5N7J2_SALET  YRGRGLIQITGLENYT
tr|Q8HA86|Q8HA86_9CAUD  YRGRGLIQITGLENYT
tr|B4TNF9|B4TNF9_SALSV  YRGRGLIQITGLENYT
tr|B5CCN5|B5CCN5_SALET  YRGRGLIQITGLENYT
tr|B4TMP0|B4TMP0_SALSV  YRGRGLIQITGLHNYR
tr|B5CBX4|B5CBX4_SALET  YRGRGLIQITGLHNYR
tr|Q06WC1|Q06WC1_SALTY  YRGRGLIQITGLHNYR
tr|C4TVC5|C4TVC5_YERKR  YRGRGLIQITGLDNYR
tr|D2TB12|D2TB12_ERWP6  YRGRGLIQVTGLENYR
tr|D0FSC0|D0FSC0_ERWPY  YRGRGLIQVTGLENYR
tr|A9ML97|A9ML97_SALAR  YRGRGLLQITGRENYT
tr|Q207V1|Q207V1_HARVE  FRGRGAMQLTHRSNYA
tr|Q207V0|Q207V0_HARVE  FRGRGAMQLTHRSNYA
tr|D2TE20|D2TE20_ERWP6  YIGRGLLHLTGRENYQ
tr|C8ZLP4|C8ZLP4_ERWPY  YIGRGLLHLTGRENYQ
tr|D2TE64|D2TE64_ERWP6  YIGRGLLHLTGRENYQ
tr|C8ZLP8|C8ZLP8_ERWPY  YIGRGLLHLTGRENYQ
tr|Q2SLQ7|Q2SLQ7_HAHCH  FKGRGLIQLTGRSNYS
tr|Q82VB8|Q82VB8_NITEU  YKGRGLIQLTGRANYR
tr|Q5ZGC9|Q5ZGC9_9CAUD  FKGRGYIQVTGRYNYT
tr|Q2G4P1|Q2G4P1_NOVAD  YLGRGPIQITGRANYR
tr|D2BY21|D2BY21_DICD5  YRGRGLIQLTGRDNYR
tr|C6CH56|C6CH56_DICZE  YRGRGLIQLTGRDNYR
tr|C6C827|C6C827_DICDC  FRGRGLIQLTGRDNYR
tr|C4RAI8|C4RAI8_9PROT  YRGRGLIQVTGKTNYA
tr|A4TVX0|A4TVX0_9PROT  YRGRGLIQVTGKANYA
tr|Q4KA35|Q4KA35_PSEF5  YRGRGLIQLTGANNYR
tr|B5LAX5|B5LAX5_9BURK  YRGRGLIQLTGRDNYE
tr|C6MHH7|C6MHH7_9PROT  YRGRGYIQLTGKDNYS
tr|A4NUP3|A4NUP3_HAEIN  YRGRGLIQITGKDNYA
tr|Q56AZ0|Q56AZ0_TREHY  YRGRGIIQLTGKNNYK
tr|B9USB1|B9USB1_BRAHW  YRGRGIIQLTGKNNYK
tr|B9USE1|B9USE1_9SPIR  YRGRGIIQLTGKNNYE
tr|C1QF51|C1QF51_9SPIR  YRGRGIIQLTGKNNYK
tr|C1QEU5|C1QEU5_9SPIR  YRGRGIIQLTGKNNYK
tr|C1QGI4|C1QGI4_9SPIR  YRGRGIIQLTGKNNYK
tr|Q8PQ62|Q8PQ62_XANAC  YRGRGYIQLTGKDQYR
tr|Q3BYD6|Q3BYD6_XANC5  YRGRGYIQLTGKDQYR
tr|B2FJG3|B2FJG3_STRMK  YHGRGYLPLVGKENYE
tr|B4SNB5|B4SNB5_STRM5  YHGRGYLPLVGKENYE
tr|C1D6N5|C1D6N5_LARHH  YRGRGFIQLTGRDNYA
tr|Q5GX30|Q5GX30_XANOR  YKGKGIIQLTWKGTYN
tr|B2SI96|B2SI96_XANOP  YKGKGIIQLTWKGTYN
tr|A4XNG6|A4XNG6_PSEMY  YKGRGLIQITWKSAYQ
tr|B8IAI0|B8IAI0_METNO  FYGRGDVQLTHERNYI
tr|B8IIX3|B8IIX3_METNO  FYGRGDVQLTHERNYI
tr|B9JVZ7|B9JVZ7_AGRVS  WLGRGYVQLTHKGNYN
tr|D2PVE1|D2PVE1_9ACTO  YGGRGYIQLTGDFNYG
```

tr|B0VVF0|B0VVF0_ACIBS YKGRGLMQITYKKNYE
