# Supplementary material for: Structural Relationships in the Lysozyme Superfamily: Significant Evidence for Glycoside Hydrolase Signature Motifs
Source: PLoS One. 2010 Nov 9;5(11):e15388. doi: 10.1371/journal.pone.0015388 (PMC2976769; doi:10.1371/journal.pone.0015388)
Supplement: Figure S2 — Multiple alignment of GH22c motif sequences. (PDF) [file pone.0015388.s003.pdf]

Figure S2. Multiple alignment of GH22c motif sequences.

```
                                    10
                          ....|....|...
tr|B8YK79|B8YK79_CHICK  DYGILQINSRWWC
tr|B8YK77|B8YK77_GALLA  DYGILQINSRWWC
tr|B8YK75|B8YK75_GALSO  DYGILQINSRWWC
tr|B8YJN9|B8YJN9_CHICK  DSGILQINSRWWC
tr|B8YJP1|B8YJP1_CHICK  DSGILQINSRWWC
tr|B8YJT7|B8YJT7_CHICK  DFGILQINSRWWC
tr|B8YK73|B8YK73_GALVA  HYGILQINSRWWC
tr|B8YK69|B8YK69_BAMTH  HYGILQINSRWWC
tr|B8YK71|B8YK71_FRAPO  YYGIPQINSRWWC
tr|P84496|P84496_ALOAE  DYGILQINSRWWC
tr|Q6GPX0|Q6GPX0_XENLA  DYGILQINSRWWC
tr|B5XA65|B5XA65_SALSA  DYGIFQINSRYWC
tr|Q9DD55|Q9DD55_SALSA  DYGIFQINSRYWC
tr|C1BZN5|C1BZN5_ESOLU  DYGIFQINSRWWC
tr|A7M773|A7M773_9PLEU  DYGIFQINSRWWC
tr|B3VHW3|B3VHW3_PSEMX  DYGIFQINSRWWC
tr|Q4RZF0|Q4RZF0_TETNG  DYGIFQINSRWWC
tr|C1K9I4|C1K9I4_PERFV  DYGIFQINSRWWC
tr|Q2PZ29|Q2PZ29_SOLSE  DYGIFQINSRYWC
tr|B0LVZ3|B0LVZ3_SOLSE  DYGIFQMNSRYWC
tr|B5LV45|B5LV45_OREAU  DFGIFQINSYWWC
tr|B5LV44|B5LV44_OREAU  DFGIFQINNRWWC
tr|B5AE24|B5AE24_OREAU  DFGIFQINSRWWC
tr|C3V6M9|C3V6M9_ORYLA  DYGIFQINSYWWC
tr|A4Z8Q3|A4Z8Q3_SHEEP  DYGIFQINSHWWC
tr|C9EJ83|C9EJ83_SHEEP  DYGIFQINSHWWC
tr|D0ETJ4|D0ETJ4_CAPHI  DYGIFQINSHWWC
tr|A4Z8Q1|A4Z8Q1_BUBBU  DYGIFQINSRWWC
tr|B2R4C5|B2R4C5_HUMAN  DYGIFQINSRYWC
tr|Q0MRP5|Q0MRP5_9CETA  DYGIFQINSRWWC
tr|B8R1K4|B8R1K4_BOSMU  DYGIFQINSRWWC
tr|A9NJ73|A9NJ73_FELCA  DYGIFQINSRYWC
tr|Q6PDV1|Q6PDV1_RAT    DYGIFQINSRYWC
tr|Q29575|Q29575_PIG    DYGIFQINSRYWC
tr|A5HKM9|A5HKM9_BUBBU  DYGIFQINSKWWC
tr|A4Z8Q2|A4Z8Q2_BUBBU  DYGIFQINSKWWC
tr|D2HIV6|D2HIV6_AILME  DYGIFQINSRYWC
tr|B2RYD4|B2RYD4_RAT    DYGIFQINSRYWC
tr|Q8BM26|Q8BM26_MOUSE  SYGIFQINSRFWC
tr|Q8BM27|Q8BM27_MOUSE  SYGIFQINSRFWC
tr|Q9D249|Q9D249_MOUSE  SYGIFQINSRFWC
tr|Q5M8G0|Q5M8G0_XENTR  EYGIFQINSYWWC
tr|Q6DIU1|Q6DIU1_XENTR  EYGIFQINSYWWC
tr|Q90WE9|Q90WE9_RANCA  EYGIFQINSYWWC
tr|C1C526|C1C526_RANCA  DYGIFQINSKWWC
tr|B5KFT9|B5KFT9_TAEGU  DYGIFQINSKYWC
tr|D2H7P7|D2H7P7_AILME  EYGIFQLSSAWWC
tr|D2HWT4|D2HWT4_AILME  DYGIFQLNNKWWC
tr|A8MZ93|A8MZ93_HUMAN  NNGIFQINSRRWC
tr|B6VH81|B6VH81_CEBAP  NNGIFQINSRRWC
tr|B4DYD1|B4DYD1_HUMAN  NNGIFQINSRRWC
tr|D2GU68|D2GU68_AILME  NNGIFQINSRKWC
tr|D2HCM0|D2HCM0_AILME  DYGIFQINSFTWC
tr|Q24JW2|Q24JW2_DANRE  DYGIFQINSFKWC
tr|Q90YS5|Q90YS5_DANRE  DYGIFQINSFKWC
tr|Q9IBG5|Q9IBG5_CYPCA  DYGIFQINSFKWC
tr|B5AC76|B5AC76_CTEID  DYGIFQINSFKWC
tr|A2A9H7|A2A9H7_MOUSE  DYGIFQINSRYWC
tr|Q4R8K7|Q4R8K7_MACFA  DYGLFQINGHYWC
tr|Q78ID0|Q78ID0_MOUSE  GFGLFQIRDNEWC
tr|D2I648|D2I648_AILME  GYGLFQIRSHEWC
tr|Q86Q32|Q86Q32_DERVA  DYGIFQINNGYWC
tr|Q86L96|Q86L96_DERAN  DYGIFQINNGYWC
tr|B7P0W4|B7P0W4_IXOSC  DFGIFQINNGYWC
tr|Q685C9|Q685C9_9SCOR  EYGIFQISSRYWC
tr|Q685C4|Q685C4_9SCOR  EYGIFQISSRYWC
tr|Q685B2|Q685B2_9SCOR  EYGIFQISSRYWC
tr|Q685B3|Q685B3_9SCOR  EYGIFQISSRYWG
tr|Q685B0|Q685B0_9SCOR  EYGIFQISSRYWC
tr|Q685A2|Q685A2_9SCOR  EYGIFQISSRYWC
tr|Q685F2|Q685F2_9SCOR  EYGIFQISSRYWC
tr|Q685F3|Q685F3_9SCOR  EYGIFQISSRYWC
```

tr|Q685C8|Q685C8_9SCOR	EYGIFQISSRYWC
tr|Q685E0|Q685E0_9SCOR	EYGIFQISSRYWC
tr|Q685F9|Q685F9_9SCOR	EYGIFQISSRYWC
tr|Q685F6|Q685F6_9SCOR	EYGIFQISSRYWC
tr|Q685E5|Q685E5_9SCOR	EYGIFQISSRYWC
tr|Q685D9|Q685D9_9SCOR	EYGIFQISSRYWC
tr|Q685E1|Q685E1_9SCOR	EYGIFQISSRYWC
tr|Q685B1|Q685B1_9SCOR	EYGISQISSRYWG
tr|Q685A1|Q685A1_9SCOR	EYGISQISSRYRC
tr|Q685G3|Q685G3_BUTEU	EYGIFQISSRYWC
tr|Q685A9|Q685A9_9SCOR	EYGIFQISSRYWC
tr|Q685A7|Q685A7_9SCOR	EYGIFQISSRYWC
tr|Q685C1|Q685C1_9SCOR	EYGIFQISSRYWC
tr|Q685E2|Q685E2_9SCOR	EYGIFQINNRYWC
tr|Q685E7|Q685E7_9SCOR	EYGIFQTSTRYWW
tr|Q685E4|Q685E4_9SCOR	EYGRFQISNRYWC
tr|Q685F1|Q685F1_9SCOR	EYGIFQISSRYWC
tr|Q685F0|Q685F0_9SCOR	EYGIFQISSRYWW
tr|Q685C2|Q685C2_9SCOR	EYGIFQISSRYWC
tr|Q685G2|Q685G2_9SCOR	EYGIFQISSRYWC
tr|Q685A4|Q685A4_9SCOR	KYGIFQISSRYWC
tr|Q6IUF5|Q6IUF5_BRABE	DHGLFQINDYYWC
tr|Q86QP2|Q86QP2_BRABE	DHELFQINDYYWC
tr|C3ZXI8|C3ZXI8_BRAFL	DHGLFQINDHYWC
tr|C3ZFG3|C3ZFG3_BRAFL	DYGLFQINGYYWC
tr|Q86M91|Q86M91_ANOST	DYGIFQINNKFWC
tr|B2MV88|B2MV88_9DIPT	DYGIFQINNKYWC
tr|O02418|O02418_ANODA	DYGLFQINNKYWC
tr|Q6GU92|Q6GU92_ANOGA	DYGIFQINNAYWC
tr|B6DDP3|B6DDP3_ANODA	DYGLFQINNRYWC
tr|B6DDP4|B6DDP4_ANODA	DYGLFQINNRYWC
tr|Q7QJ81|Q7QJ81_ANOGA	DYGIFQINNYYWC
tr|Q6GU91|Q6GU91_ANOGA	DYGIFQINNYYWC
tr|A0NBE9|A0NBE9_ANOGA	YYGLFQLQSAYHC
tr|Q4ZJA6|Q4ZJA6_ANOGA	YYGLFQLQSAYHC
tr|A9LN32|A9LN32_RHOPR	DYGLFQINDHIWC
tr|Q26363|Q26363_MANSE	DYGLFQINDKYWC
tr|Q9TWY1|Q9TWY1_MANSE	DYGLFQINDKYWC
tr|Q8I808|Q8I808_AGRCO	DYGLFQINDKYWC
tr|Q2L7D2|Q2L7D2_ANTPE	DYGLFQINDKYWC
tr|Q6QEJ9|Q6QEJ9_9NEOP	DYGLFQINDKYWC
tr|Q9GNL4|Q9GNL4_SAMCR	DYGLFQINDKYWC
tr|B8YI23|B8YI23_HELZE	DYGLYQINDKYWC
tr|Q19R28|Q19R28_HELAM	DYGLYQINDKYWC
tr|O96862|O96862_HELVI	DYGLYQINDKYWC
tr|B6DZS0|B6DZS0_SPOLT	DYGLFQINDKYWC
tr|Q86FK1|Q86FK1_SPOEX	DYGLFQINDKYWC
tr|Q6QMF0|Q6QMF0_PSEIC	DYGLFQINDKYWC
tr|Q68KS6|Q68KS6_9NEOP	DYGLFQINDKYWC
tr|A9P5Q4|A9P5Q4_9NEOP	DYGLFQINDKYWC
tr|A9XXB6|A9XXB6_TRINI	DYGLFQINDNIWC
tr|B0WEH8|B0WEH8_CULQU	DWGLFQINDRYWC
tr|B0WEH9|B0WEH9_CULQU	DWGLFQINDRYWC
tr|B8RJ76|B8RJ76_CULTA	DWGLFQINDRYWC
tr|Q8T3T7|Q8T3T7_AEDAL	DWGLFQINDRYWC
tr|Q9GRF3|Q9GRF3_AEDAE	DWGLFQINDRYWC
tr|Q672J4|Q672J4_AEDAE	DWGLFQINDRYWC
tr|Q16V63|Q16V63_AEDAE	DYGLFQINNKYWC
tr|Q8T9V5|Q8T9V5_AEDAE	DYGLFQINNKYWC
tr|Q5MIY9|Q5MIY9_AEDAL	DYGLFQINNKYWC
tr|A0FIV6|A0FIV6_9DIPT	DYGLFQINNRYWC
tr|D1FPY3|D1FPY3_9DIPT	DYGLFQINSKYWC
tr|D1FPX6|D1FPX6_9DIPT	DYGLFQINSKYWC
tr|B5M0V6|B5M0V6_SIMVI	DYGLFQINSKYWC
tr|Q16TW8|Q16TW8_AEDAE	SYGIFQINSKQWC
tr|Q1DGZ3|Q1DGZ3_AEDAE	SYGIFQINSKQWC
tr|B0WUD1|B0WUD1_CULQU	SYGIFQINSKEWC
tr|Q7PT23|Q7PT23_ANOGA	NYGIFQINSKEWC
tr|Q7QJA6|Q7QJA6_ANOGA	NYGIFQINSKTWC
tr|B0WUD2|B0WUD2_CULQU	SYGIFQINSKEWC
tr|A1BW46|A1BW46_DROPS	SYGLFQINSKNWC
tr|A1BW47|A1BW47_DROMI	SYGLFQINSKNWC
tr|A1BW65|A1BW65_DROMI	SYGLFQINSKNWC
tr|A1BW45|A1BW45_DROAI	SYGLFQINSKNWC
tr|Q2PQQ7|Q2PQQ7_GLOMM	SYGLFQINSKNWC
tr|A5A143|A5A143_BOMMO	SYGLFQINNKDWC
tr|A5A142|A5A142_ANTMY	YYGLFQIGS-EWC

```
tr|D1KRL0|D1KRL0_MANSE  YYGLFQIGS-EWC
tr|B4GWI7|B4GWI7_DROPE  SLGLFQINSR-YC
tr|Q29I82|Q29I82_DROPS  SLGLFQINSR-YC
tr|Q9W4C2|Q9W4C2_DROME  NYGLFQINGR-FC
tr|B4I0P3|B4I0P3_DROSE  NYGLFQINGR-FC
tr|B3NV44|B3NV44_DROER  SYGLFQINGR-FC
tr|B4PZQ1|B4PZQ1_DROYA  NYGLFQINGR-FC
tr|B4L4L9|B4L4L9_DROMO  NYGLFQINER-YC
tr|B4M2J4|B4M2J4_DROVI  SYGLFQINGR-YC
tr|B4JN62|B4JN62_DROGR  NYGLFQINTR-FC
tr|B4MUJ9|B4MUJ9_DROWI  NYGLFQINGR-YC
tr|A1BWC8|A1BWC8_DROMI  NYGLFQINSKDYC
tr|A1BWC3|A1BWC3_DROMI  NYGLFQINSKDYC
tr|A1BWC2|A1BWC2_DROPS  NYGLFQINSKDYC
tr|B5E170|B5E170_DROPS  NYGLFQINSKDYC
tr|B4GGZ3|B4GGZ3_DROPE  NYGLFQINSKDYC
tr|B3MIT6|B3MIT6_DROAN  NYGLFQINSKNYC
tr|C5WLP0|C5WLP0_DROME  NYGLFQINSKDYC
tr|C0HBU0|C0HBU0_DROME  NYGLFQINSKDYC
tr|A1ZBX6|A1ZBX6_DROME  NYGLFQINSKDYC
tr|B4HQN0|B4HQN0_DROSE  NYGLFQINSKDYC
tr|B4QF38|B4QF38_DROSI  NYGLFQINSKDYC
tr|B3NJW6|B3NJW6_DROER  NYGLFQINNRDYC
tr|B4PA33|B4PA33_DROYA  NYGLFQINSKDYC
tr|B4MPJ4|B4MPJ4_DROWI  NYGLFQINSKDYC
tr|B4ME97|B4ME97_DROVI  NYGLFQINSRDYC
tr|A0SLC3|A0SLC3_MAYDE  SYGLFQINSKDYC
tr|Q95V66|Q95V66_LITVA  DYGIFQINNKYWC
tr|C0IXV4|C0IXV4_LITST  DYGIFQINNKYWC
tr|Q8IT75|Q8IT75_PENMO  DYGIFQINNKYWC
tr|Q86D81|Q86D81_MACRS  DYGIFQINNKHWC
tr|Q86D80|Q86D80_MACNP  DYGIFQINNKYWC
tr|A7XUF7|A7XUF7_PENMO  DYGIFQINNKYWC
tr|Q4KY21|Q4KY21_FENCH  DYGIFQINNKYWC
tr|C1IIX1|C1IIX1_FENME  DYGIFQINNKYWC
tr|C8CK83|C8CK83_FENIN  DYGIFQINNKYWC
tr|Q8I7X3|Q8I7X3_PENSE  DYGIFQINNKYWC
tr|Q86SC1|Q86SC1_PENJP  DYGIFQINNKYWC
tr|Q8WSQ8|Q8WSQ8_PENJP  DYGIFQINNKYWC
tr|B9VQ21|B9VQ21_9EUCA  DYGLFQLNNKYWC
tr|Q177Z3|Q177Z3_AEDAE  YHGMFQLSDEYWC
tr|B0W3Z5|B0W3Z5_CULQU  YHGMFQLSDEYWC
tr|Q7Q6R2|Q7Q6R2_ANOGA  YHGMFQLSDEYWC
tr|C8C411|C8C411_TRICA  DHGLFQISQIYWC
tr|C8C412|C8C412_TRICA  DHGLFQISQIYWC
tr|B6RQP3|B6RQP3_SITZE  DYGILQISEKFWC
tr|Q9VSA5|Q9VSA5_DROME  DHGLFQISDLYWC
tr|B4QKX1|B4QKX1_DROSI  DHGLFQISDLFWC
tr|B4HIV3|B4HIV3_DROSE  DHGLFQISDLYWC
tr|B4PCB0|B4PCB0_DROYA  DHGLFQISDLYWC
tr|B3M961|B3M961_DROAN  DHGLFQISDLFWC
tr|B4H299|B4H299_DROPE  DHGLFQISDLYWC
tr|B5DR06|B5DR06_DROPS  DHGLFQISDLYWC
tr|B4KWW1|B4KWW1_DROMO  DHGLFQISDLYWC
tr|B4LH11|B4LH11_DROVI  DHGLFQISDLYWC
tr|B4IWP9|B4IWP9_DROGR  DHGLFQISDLYWC
tr|B4MY10|B4MY10_DROWI  DHGLFQISDLYWC
tr|D2KMR3|D2KMR3_BOMMO  TRPTTAITAKYWT
tr|A5H9H9|A5H9H9_9NEOP  DYGLFQINDRYWC
tr|Q8IAD0|Q8IAD0_9NEOP  DYGLFQINDRYWC
tr|Q8IAD1|Q8IAD1_9NEOP  DYGLFQINDRYWC
tr|A5H9H7|A5H9H7_9NEOP  DYGLFQINDRYWC
tr|B4J5J4|B4J5J4_DROGR  DWGLFQINDRYWC
tr|B4LKY2|B4LKY2_DROVI  DWGLFQINDRYWC
tr|B4KPK0|B4KPK0_DROMO  DWGLFQINDRYWC
tr|B3NPV1|B3NPV1_DROER  DWGLFQINDRYWC
tr|B4P6B7|B4P6B7_DROYA  DWGLFQINDRYWC
tr|B4QHH6|B4QHH6_DROSI  DWGLFQINDRYWC
tr|A1ZAB8|A1ZAB8_DROME  DWGLFQINDRYWC
tr|B4HST5|B4HST5_DROSE  DWGLFQINDRYWC
tr|B3MH65|B3MH65_DROAN  DWGLFQINDRYWC
tr|Q28Z62|Q28Z62_DROPS  DWGLFQINDRYWC
tr|B4GI84|B4GI84_DROPE  DWGLFQINDRYWC
tr|B4L917|B4L917_DROMO  DYGIFQINDRYWC
tr|B4L918|B4L918_DROMO  DYGIFQINDRYWC
tr|B4LCC7|B4LCC7_DROVI  DYGIFQINDRYWC
tr|A4V9X3|A4V9X3_DROSI  DYGIFQINDLYWC
```

```
tr|A4V9X4|A4V9X4_DROSI  DYGIFQINDLYWC
tr|A4V9X6|A4V9X6_DROSI  DYGIFQINDLYWC
tr|A4V9X5|A4V9X5_DROSI  DYGIFQINDLYWC
tr|B4QLX5|B4QLX5_DROSI  DYGVFQINDLYWC
tr|B3NJF2|B3NJF2_DROER  DYGIFQINDLYWC
tr|B4PCR4|B4PCR4_DROYA  DYGIFQINDLYWC
tr|B4LCC9|B4LCC9_DROVI  DYGIFQINNYYWC
tr|B4LCC8|B4LCC8_DROVI  DYGIFQINDYYWC
tr|B4L916|B4L916_DROMO  DYGIFQINNYYWC
tr|B4L913|B4L913_DROMO  DYGIFQINNYYWC
tr|B4LCC5|B4LCC5_DROVI  DYGIFQINNYYWC
tr|B5DR84|B5DR84_DROPS  DYGIFQINDYYWC
tr|B4H5I6|B4H5I6_DROPE  DYGIFQINDYYWC
tr|B5DR88|B5DR88_DROPS  DYGIFQINDYYWC
tr|B5DR91|B5DR91_DROPS  DYGIFQINDYYWC
tr|B4H5I8|B4H5I8_DROPE  DYGIFQINDYYWC
tr|B4PD47|B4PD47_DROYA  DYGIFQINDYYWC
tr|B4PD44|B4PD44_DROYA  DYGIFQINDYYWC
tr|B4HVP1|B4HVP1_DROSE  DYGIFQINDYYWC
tr|B3NJF7|B3NJF7_DROER  DYGIFQINDYYWC
tr|B4QLX9|B4QLX9_DROSI  DYGIFQINDYYWC
tr|B4HVP0|B4HVP0_DROSE  DYGIFQINDYYWC
tr|B4PD45|B4PD45_DROYA  DYGIFQINDYYWC
tr|B4MXX7|B4MXX7_DROWI  DYGIFQINDYYWC
tr|B5DR89|B5DR89_DROPS  DYGIFQINDYYWC
tr|B4H5I7|B4H5I7_DROPE  DYGIFQINDYYWC
tr|B5DR85|B5DR85_DROPS  DYGIFQINDYYWC
tr|B5DR87|B5DR87_DROPS  DYGIFQINDYYWC
tr|B4H5I9|B4H5I9_DROPE  DYGIFQINDYYWC
tr|B4LCD1|B4LCD1_DROVI  DYGIFQINDLYWC
tr|B4PD49|B4PD49_DROYA  DYGIFQINDLYWC
tr|Q6XHI6|Q6XHI6_DROYA  DYGIFQINDLYWC
tr|B4NSY7|B4NSY7_DROSI  DYGIFQINDLYWC
tr|B3DMZ0|B3DMZ0_DROME  DYGIFQINDLYWC
tr|Q29EU6|Q29EU6_DROPS  DYGIFQINDLYWC
tr|B4MXY1|B4MXY1_DROWI  DYGIFQINDLYWC
tr|B3M543|B3M543_DROAN  DYGIFQINDLYWC
tr|B3M549|B3M549_DROAN  DYGIFQINNYYWC
tr|B3M548|B3M548_DROAN  DYGIFQINNYYWC
tr|B3M545|B3M545_DROAN  DYGIFQINNYYWC
tr|B5DR81|B5DR81_DROPS  DYGIFQINNYYWC
tr|B3NJG0|B3NJG0_DROER  DYGIFQINNKYWC
tr|B4PD48|B4PD48_DROYA  DYGIFQINNKYWC
tr|B3M544|B3M544_DROAN  DYGIFQINNKYWC
tr|B4H5J1|B4H5J1_DROPE  DYGIFQINDYYWC
tr|B5DR82|B5DR82_DROPS  AYGIFQINDYYWC
tr|B3M554|B3M554_DROAN  DYGLFQINDRYWC
tr|B3GQR5|B3GQR5_MUSDO  DYGIFQINNYYWC
tr|Q7YT17|Q7YT17_MUSDO  DYGIFQINNYYWC
tr|B4J6D4|B4J6D4_DROGR  DHGIFQINDRIWC
tr|B5DR83|B5DR83_DROPS  NYGIFQISNEYWC
tr|B4JLE8|B4JLE8_DROGR  NYGIFQIGN-HWC
tr|B4J6D3|B4J6D3_DROGR  NYGIFQISDGWWC
tr|A1Z9D5|A1Z9D5_DROME  DYGLFQISDRYWC
tr|Q2TPW4|Q2TPW4_TRIBS  DNGLFQINDRYWC
tr|Q7YZS5|Q7YZS5_TRIIF  DNGLFQINDRFWC
tr|A9LN31|A9LN31_RHOPR  DNGLFQINDRIWC
tr|A9X7P1|A9X7P1_TRIIF  YYGLFQISDRYWC
tr|B7PQG4|B7PQG4_IXOSC  DHGIFQINDKHWC
tr|D2HWT5|D2HWT5_AILME  EYGLFQINNKFWC
```
