# Supplementary material for: Structural Relationships in the Lysozyme Superfamily: Significant Evidence for Glycoside Hydrolase Signature Motifs
Source: PLoS One. 2010 Nov 9;5(11):e15388. doi: 10.1371/journal.pone.0015388 (PMC2976769; doi:10.1371/journal.pone.0015388)
Supplement: Figure S3 — Multiple alignment of GH22i motif sequences. (PDF) [file pone.0015388.s004.pdf]

Figure S3. Multiple alignment of GH22i motif sequences.

```
                               ....|....
tr|Q8IU26|Q8IU26_VENPH  LSCGYFQIK
tr|C8CBP0|C8CBP0_VENPH  LSCGYFQIK
tr|Q9BLE0|Q9BLE0_VENPH  LSCGYFQIK
tr|Q8ITT7|Q8ITT7_9BIVA  LSCGYFQIK
tr|Q8ITT8|Q8ITT8_9BIVA  LSCGYFQIK
tr|Q8ITU2|Q8ITU2_MYTED  LSCGPFQIK
tr|Q8ITT9|Q8ITT9_MYTGA  LSCGPFQIK
tr|D0QS77|D0QS77_9BIVA  LSCGPFQIK
tr|Q8ITU0|Q8ITU0_9BIVA  LSCGPFQIK
tr|Q8ITU1|Q8ITU1_9BIVA  LSCGPFQIK
tr|D0QSI1|D0QSI1_9BIVA  LSCGPFQIK
tr|D0QS81|D0QS81_9BIVA  LSCGPFQIK
tr|D0QSJ1|D0QSJ1_9BIVA  LSCGPFQIK
tr|D0QS79|D0QS79_9BIVA  LSCGPFQIK
tr|D0QS75|D0QS75_9BIVA  LSCGPFQIK
tr|D0QSB7|D0QSB7_9BIVA  LSCGPFQIK
tr|D0QSA8|D0QSA8_9BIVA  LSCGPFQIK
tr|D0QS86|D0QS86_9BIVA  LSCGPFQIK
tr|A5LHX1|A5LHX1_MYTGA  NSCGYMQIK
tr|Q2XPU8|Q2XPU8_MYTED  NSCGYMQIK
tr|Q9BI29|Q9BI29_CHLIS  DSCGYFQLK
tr|Q9TP22|Q9TP22_CHLIS  DSCGYFQLK
tr|A3KDX2|A3KDX2_CRAGI  ESCGYYQIK
tr|A9CPZ8|A9CPZ8_CRAGI  ESCGYYQIK
tr|A3KDX3|A3KDX3_CRAGI  YSCGYFQIK
tr|O76358|O76358_CAEEL  LSCGYYQIK
tr|O76357|O76357_CAEEL  LSCGYYQIK
tr|Q19698|Q19698_CAEEL  LSCGYYQIK
tr|A8XC38|A8XC38_CAEBR  LSCGYYQIK
tr|Q9GYQ2|Q9GYQ2_CAEEL  IGCGYFRLN
tr|A8XRH9|A8XRH9_CAEBR  IGCGYFRLN
tr|A8PY35|A8PY35_BRUMA  YGCGYFRLN
tr|Q0ZME1|Q0ZME1_9ANNE  LSCGPFQIK
tr|Q25091|Q25091_HIRME  LSCGPYQIK
tr|Q6TP50|Q6TP50_ASTRU  LSCGPYQIK
tr|A0MT08|A0MT08_STIJA  LSCGPYQIK
tr|C3ZBZ4|C3ZBZ4_BRAFL  LSCGPFQIQ
tr|C3ZBZ3|C3ZBZ3_BRAFL  LSCGPFQIQ
tr|C3YM12|C3YM12_BRAFL  DSCGPYQIK
```
