# Supplementary material for: Structural Relationships in the Lysozyme Superfamily: Significant Evidence for Glycoside Hydrolase Signature Motifs
Source: PLoS One. 2010 Nov 9;5(11):e15388. doi: 10.1371/journal.pone.0015388 (PMC2976769; doi:10.1371/journal.pone.0015388)
Supplement: Figure S4 — Multiple alignment of GH23 motif sequences. (PDF) [file pone.0015388.s005.pdf]

# Figure S4. Multiple alignment of GH23 motif sequences.

```
                                      10
                             ....|....|
tr|P84503|P84503_ANSCY  NGFGLMQVDK
tr|P84504|P84504_RHEAM  NGFGLMQVDR
tr|Q5FW09|Q5FW09_XENTR  NAFGLMQVDK
tr|Q5XK71|Q5XK71_XENLA  NAFGLMQVDK
tr|Q1M163|Q1M163_GADMO  NGFGLMQVDK
tr|B9TU22|B9TU22_GADMO  NGFGLMQVDK
tr|A6PYF4|A6PYF4_GADMO  NGFGLMQVDK
tr|A6PYF3|A6PYF3_GADMO  IGFGLMQVDK
tr|C1BHT6|C1BHT6_ONCMY  NGFGLMQVDK
tr|C1BHK0|C1BHK0_ONCMY  NGFGLMQVDK
tr|C1BGU6|C1BGU6_ONCMY  NGFGLMQVDK
tr|A6PZ97|A6PZ97_SALSA  KGFGLMQVDK
tr|B5XET9|B5XET9_SALSA  KGFGLMQVDK
tr|B5XAN4|B5XAN4_SALSA  KGFGLMQVDK
tr|B5XCN8|B5XCN8_SALSA  KGFGLMQVDK
tr|B5XDU9|B5XDU9_SALSA  KGFGLMQVDK
tr|B5X9P5|B5X9P5_SALSA  KGFGLMQVDK
tr|B5XA96|B5XA96_SALSA  KGFGLMQVDK
tr|C1BW35|C1BW35_ESOLU  NGFGLMQVDK
tr|Q0KFS4|Q0KFS4_SALSA  KGFGLMQVDK
tr|C1BL43|C1BL43_OSMMO  NGFGLMQVDK
tr|Q6DH80|Q6DH80_DANRE  NGFGLMQVDK
tr|B5AFK7|B5AFK7_LABRO  NGFGLMQVDK
tr|A3KP47|A3KP47_DANRE  NGFGLMQVDK
tr|B5AC75|B5AC75_CTEID  YAFGIMQIDK
tr|C3XXG2|C3XXG2_BRAFL  NGYGLMQVDI
tr|C3XXG0|C3XXG0_BRAFL  NGYGLMQVDI
tr|C3YR54|C3YR54_BRAFL  NGFGLMQIDR
tr|B3RIZ1|B3RIZ1_TRIAD  NGYGLMQVDK
tr|Q08NK4|Q08NK4_STIAU  NGFGLMQVDK
tr|A8D9Q3|A8D9Q3_LARCR  NAWGLMQVDV
tr|A8D9Q7|A8D9Q7_LARCR  NAWGLMQVDV
tr|Q5XU03|Q5XU03_SINCH  NGWGLMQVDV
tr|A7M774|A7M774_9PLEU  NAWGLMQVDV
tr|B1B725|B1B725_SOLSE  NAWGLMQVDV
tr|A8D3J6|A8D3J6_LATCA  KAWGLMQVDV
tr|B2RFG5|B2RFG5_SPAAU  NAWGLMQVDV
tr|Q4RZR7|Q4RZR7_TETNG  KAWGLMQVDV
tr|B9ELT2|B9ELT2_SALSA  KAFGLMQIDT
tr|C1BF55|C1BF55_ONCMY  RGFGLMQVDV
tr|C1BQ11|C1BQ11_9MAXI  NAFGLMQVDK
tr|C1BHE7|C1BHE7_ONCMY  NAFGLMQVDK
tr|B2ZSY1|B2ZSY1_ANAPL  NAFGLMQVDK
tr|C3YYL0|C3YYL0_BRAFL  NGFGLMQVDK
tr|D2HDC7|D2HDC7_AILME  LKFGLMQLDK
tr|A5PJT9|A5PJT9_BOVIN  LKFGLMQLDK
tr|C9J4J0|C9J4J0_HUMAN  LKFGLMQLDK
tr|C9JBA4|C9JBA4_HUMAN  LKFGLMQLDK
tr|Q496G2|Q496G2_HUMAN  LKFGLMQLDK
tr|D2HDC6|D2HDC6_AILME  DGIGVVQ-DP
tr|Q53RV9|Q53RV9_HUMAN  DRTSMVQ-DP
tr|Q0VE18|Q0VE18_MOUSE  SGLGMVK-ET
tr|Q4T499|Q4T499_TETNG  NCFGLMQINK
tr|Q7YXC1|Q7YXC1_OIKDI  YGWGLMQVDR
tr|Q7YXC2|Q7YXC2_OIKDI  YGWGLMQVDR
tr|Q7YXC3|Q7YXC3_OIKDI  YGWGLMQVDR
tr|Q075V3|Q075V3_9BIVA  HAYGILQCDI
tr|Q2HNY7|Q2HNY7_AEQIR  HAYGILQCDI
tr|C4TJC8|C4TJC8_CRAGI  NAYGIMQCDV
tr|C3ZH25|C3ZH25_BRAFL  NGWGIMQVDK
tr|B0TES1|B0TES1_HELMI  -ARGLMQLMP
tr|C0GKJ2|C0GKJ2_9FIRM  -ALGLMQLMP
tr|C5VQ07|C5VQ07_CLOBO  -AIGLMQLTP
tr|A7VZ93|A7VZ93_9CLOT  -ARGLMQIMP
tr|D2Z7E0|D2Z7E0_9BACT  -ASGLMQLMP
tr|Q0VPM5|Q0VPM5_ALCBS  -AQGLMQLMP
tr|B4X1W0|B4X1W0_9GAMM  -AQGLMQLMP
tr|Q2BMQ9|Q2BMQ9_9GAMM  -AQGLMQLMP
tr|B5JXR2|B5JXR2_9GAMM  -AQGLMQLMP
tr|Q1ZHF2|Q1ZHF2_9GAMM  -AMGLMQLMP
tr|C8NDD9|C8NDD9_9GAMM  -AMGLMQLMP
tr|C7LR87|C7LR87_DESBD  -AQGLMQIMP
```

```
tr|B8DI65|B8DI65_LISMH  -ATGLMQLMA
tr|Q92DT7|Q92DT7_LISIN  -ATGLMQLMY
tr|Q722I0|Q722I0_LISMF  -ATGLMQLMY
tr|C1L0Z8|C1L0Z8_LISMC  -ATGLMQLMY
tr|C8K7E6|C8K7E6_LISMO  -ATGLMQLMY
tr|Q4ELS3|Q4ELS3_LISMO  -ATGLMQLMY
tr|Q4EU87|Q4EU87_LISMO  -ATGLMQLMY
tr|D2PAV5|D2PAV5_LISMO  -ATGLMQLMY
tr|D2NZI0|D2NZI0_LISMO  -ATGLMQLMY
tr|C8K9V3|C8K9V3_LISMO  -ATGLMQLMY
tr|C8JY83|C8JY83_LISMO  -ATGLMQLMY
tr|Q8Y925|Q8Y925_LISMO  -ATGLMQLMY
tr|Q8GMY2|Q8GMY2_LISMO  -ATGLMQLMY
tr|A0AGH2|A0AGH2_LISW6  -ATGLMQLMY
tr|O64046|O64046_BPSPC  -AMGLMQLMP
tr|B1HNX9|B1HNX9_LYSSC  -AQGLMQLMP
tr|A3IFZ4|A3IFZ4_9BACI  -AQGLMQLMP
tr|Q9K900|Q9K900_BACHD  -ASGLMQLMP
tr|Q3AF19|Q3AF19_CARHZ  -AMGLMQLMP
tr|C8W6G7|C8W6G7_DESAS  -ALGLTQLMP
tr|C0QET8|C0QET8_DESAH  -VRGLMQVTT
tr|B8FJF4|B8FJF4_DESAA  -VKGLMQITR
tr|B3QU47|B3QU47_CHLT3  -AKGLMQVVP
tr|Q11RC3|Q11RC3_CYTH3  -ACGLMQVMP
tr|Q7NWF6|Q7NWF6_CHRVO  -ASGLMQLMP
tr|C0Z4A8|C0Z4A8_BREBN  -ARGLLQIMP
tr|A7BWV5|A7BWV5_9GAMM  YGRGLMQIDY
tr|Q7VF48|Q7VF48_HELHP  -AYGLMQVVP
tr|C9PJB4|C9PJB4_VIBFU  -AFGLMQIVP
tr|A6D3Q2|A6D3Q2_9VIBR  -AVGIMQVLP
tr|Q1INF2|Q1INF2_ACIBL  HGVGLVQIDI
```
