# Supplementary material for: Structural Relationships in the Lysozyme Superfamily: Significant Evidence for Glycoside Hydrolase Signature Motifs
Source: PLoS One. 2010 Nov 9;5(11):e15388. doi: 10.1371/journal.pone.0015388 (PMC2976769; doi:10.1371/journal.pone.0015388)
Supplement: Figure S5 — Multiple alignment of GH24v motif sequences. (PDF) [file pone.0015388.s006.pdf]

Figure S5. Multiple alignment of GH24v motif sequences.

```
                                     10
                              ....|....|....|.
tr|Q7M2A4|Q7M2A4_BPT2         IYKDTEGYYTIGIGHL
tr|Q06EK2|Q06EK2_BPR32        IYKDTEGYYTIGIGHL
tr|C3V2B5|C3V2B5_BPR51        IYKDTEGYYTIGIGHL
tr|C3V1I9|C3V1I9_9CAUD        IYKDTEGYYTIGIGHL
tr|Q7Y2B5|Q7Y2B5_BPR69        LYKDTEGFWTIGIGHL
tr|C4MZK9|C4MZK9_9CAUD        LYKDTEGYWTIGIGQL
tr|A8R9C2|A8R9C2_9CAUD        LYKDTEGYWTIGIGQL
tr|Q56EM5|Q56EM5_9CAUD        VYWDSEGYPTVGIGHL
tr|Q6U9G4|Q6U9G4_9CAUD        VYWDSEGYPTVGIGHL
tr|Q19CN7|Q19CN7_9CAUD        VYWDSEGYPTVGIGHL
tr|Q6U979|Q6U979_9CAUD        VYWDHLGYPTVGIGHL
tr|Q56EE1|Q56EE1_9CAUD        VYWDHLGYPTVGIGHL
tr|Q19CF2|Q19CF2_9CAUD        VYWDHLGYPTIGIGHL
tr|Q76YA6|Q76YA6_9CAUD        VYWDTEGYPTIGIGHL
tr|Q8SDG3|Q8SDG3_9CAUD        WYLDSEGYPTIGIGHL
tr|A7XF92|A7XF92_9CAUD        WYLDSEGYPTIGIGHL
tr|C4MYV6|C4MYV6_9CAUD        WYLDSEGYPTIGIGHL
tr|Q76YN5|Q76YN5_9CAUD        WYLDSEGYPTIGIGHL
tr|Q56BJ4|Q56BJ4_9CAUD        WYYDVKGY-TIGIGHF
tr|Q06EH6|Q06EH6_BPR32        VYWDTEGYPTIGIGHL
tr|C3V1L1|C3V1L1_9CAUD        VYWDTEGYPTIGIGHL
tr|C3V2D8|C3V2D8_BPR51        VYWDTEGYPTIGIGHL
tr|Q7Y4Y4|Q7Y4Y4_BPR69        VYWDHLGYPTVGIGHL
tr|C4MZP0|C4MZP0_9CAUD        VYWDTEGYPTIGIGHL
tr|Q5QBY1|Q5QBY1_9CAUD        VYWDTEGYPTIGIGHL
tr|A4G7B2|A4G7B2_HERAR        AYQDHLGFWTIGVGIL
tr|A6T031|A6T031_JANMA        AYEDHLSFLTIGIGRL
tr|A9I970|A9I970_BORPD        AYRDHLGYLTIGVGRL
tr|B9CB79|B9CB79_9BURK        IYVDTVGKVSGGIGRN
tr|B9BQE8|B9BQE8_9BURK        IYVDTVGKVSGGIGRN
tr|B9CGW2|B9CGW2_9BURK        IYVDTVGKVSGGIGRN
tr|B9BX46|B9BX46_9BURK        IYVDTVGKVSGGIGRN
tr|B9BBE6|B9BBE6_9BURK        IYIDTVGKVSGGIGRN
tr|A2W8F0|A2W8F0_9BURK        IYVDTVGKVSGGIGRN
tr|Q2T018|Q2T018_BURTA        IYTDTVGKVSGGIGRN
tr|C5AEP5|C5AEP5_BURGB        PYVDTVGKITIGVGRN
tr|B9TDP0|B9TDP0_RICCO        PYLDTKGIQTTAVGHN
tr|B0VGF1|B0VGF1_9BACT        PYRCTAGKLTIGIGRN
tr|B0VF72|B0VF72_9BACT        PYRCTAGKLTIGIGRN
tr|Q5N403|Q5N403_SYNP6        PYRCTAGRLTIGIGRN
tr|Q31Q81|Q31Q81_SYNE7        PYRCTAGRLTIGIGRN
tr|A6F0L1|A6F0L1_9ALTE        PYLDTVGKLTVGYGRN
tr|A9MK11|A9MK11_SALAR        PYIDALGYPTVGVGFK
tr|A8T9J0|A8T9J0_9VIBR        PYYCSEGYPTIGIGQK
tr|Q5G7N2|Q5G7N2_9CAUD        PYKDSLGYPTIGYGIK
tr|C8PDP7|C8PDP7_9PROT        IYKDSLGKATIGYGFL
tr|C8PIK2|C8PIK2_9PROT        IYEDTRGYKTIGYGFL
tr|C9PG81|C9PG81_VIBFU        PYRCSNQKLTIGYGRN
tr|A9ZHG7|A9ZHG7_COXBU        LYKDSVGKWTIGYGRN
tr|A9KG49|A9KG49_COXBN        LYKDSVSKWTIGYGRN
tr|B6J707|B6J707_COXB1        LYKDSVGKWTIGYGRN
tr|Q83CS4|Q83CS4_COXBU        LYKDSVGKWTIGYGRN
tr|A9NCQ7|A9NCQ7_COXBR        LYKDSVGKWTIGYGRN
tr|B6J063|B6J063_COXB2        LYKDSVGKWTIGYGRN
tr|A8EV01|A8EV01_ARCB4        VYKCPAGFDTIGYGRN
tr|C1SGF3|C1SGF3_9BACT        PYVCPAGKWTIGYGYN
tr|Q933T0|Q933T0_HELPY        IYTDKTGHPTIGYGYN
tr|O25106|O25106_HELPY        IYTDKTGHPTIGYGYN
tr|B6JKR4|B6JKR4_HELP2        IYTDKTGHPTIGYGYN
tr|D0JYN5|D0JYN5_HELP5        VYADKTGHPTIGYGYN
tr|B9XTI9|B9XTI9_HELPY        IYTDKTGHPTIGYGYN
tr|A9V884|A9V884_MONBE        TYVDTTGHKTICYGFN
tr|A7S7F4|A7S7F4_NEMVE        MYLDIKGIKTIGVGYN
tr|Q22BQ6|Q22BQ6_TETTH        VYLDTKGIPTIGIGFN
tr|B5QB53|B5QB53_SALVI        VYKDHLGYPTIGYGHL
tr|A7MG17|A7MG17_ENTS8        PYNDTEGFLTVGFGHK
tr|A4SN28|A4SN28_AERS4        RYKDSLGYWTIGYGHL
tr|Q8H9R6|Q8H9R6_9CAUD        WYKDSLGYWTGGYGHL
tr|A9J749|A9J749_9CAUD        WYKDSLGKITGGYGHL
tr|C6DKJ8|C6DKJ8_PECCP        KYRDTAGKWTIGYGHL
tr|Q8GCR0|Q8GCR0_PECCC        KYRDTAGKWTIGYGHL
tr|C9XUR2|C9XUR2_CROTZ        KYQDAVGKWTIGYGHL
```

tr|C6BJ95|C6BJ95_RALP1	TYLDAVGKPTIGYGHL
tr|B2UGR3|B2UGR3_RALPJ	TYLDAVGKPTIGYGHL
tr|D3F184|D3F184_9ACTN	PYDDPAGHATVGYGHL
tr|C8N775|C8N775_9GAMM	MYYDAAGLPTIGVGHL
tr|C8NCA4|C8NCA4_9GAMM	MYLDSAGLPTIGVGHL
tr|A3YYR1|A3YYR1_9SYNE	PRIDSLGNTVIGFNHV
tr|C9MTK8|C9MTK8_9BACT	PYNDSKGFATIGVGHL
tr|C7Z8W0|C7Z8W0_NECH7	IYKDAAGYPTVGYGHL
tr|Q2GND9|Q2GND9_CHAGB	VYNDPAGHPTVGYGHL
tr|C7Z967|C7Z967_NECH7	VYIDATGNPTVGYGHL
tr|C5AYQ1|C5AYQ1_METEA	AYRDSVGVWTIGIGHT
tr|A9W2L1|A9W2L1_METEP	AYRDSVGVWTIGIGHT
tr|C7CKV4|C7CKV4_METED	AYRDSVGGWTIGIGHT
tr|B7KTM6|B7KTM6_METC4	AYRDSVGVWTIGIGHT
tr|B1ZE94|B1ZE94_METPB	AYRDSVGVWTIGIGHT
tr|B0UJ17|B0UJ17_METS4	AYKDSVGVWTIGVGIT
tr|B5PT00|B5PT00_SALHA	AYPDSRGIPTIGVGHT
tr|A9MNN1|A9MNN1_SALAR	AYPDSRGIPTIGVGHT
tr|Q5G8R3|Q5G8R3_9CAUD	AYPDSRGIPTIGVGHT
tr|Q56117|Q56117_9CAUD	AYPDSRGIPTIGVGHT
tr|Q5PG10|Q5PG10_SALPA	AYPDSRGIPTIGVGHT
tr|B5FLT9|B5FLT9_SALDC	AYPDSRGIPTIGVGHT
tr|B5BDL3|B5BDL3_SALPK	AYPDSRGIPTIGVGHT
tr|B4A6C6|B4A6C6_SALNE	AYPDSRGIPTIGVGHT
tr|A8CGF4|A8CGF4_BPP22	AYSDSRGIPTIGVGHT
tr|A3VTL8|A3VTL8_9PROT	VYDDGVGIWTIGYGHT
tr|Q94ML9|Q94ML9_9CAUD	KAVPTEKHYTIGYGHY
tr|B3VMQ1|B3VMQ1_BPPH2	KAVPTEKHYTIGYGHY
tr|B7SSN6|B7SSN6_BPNF	KAVPTEKYYTIGYGHY
tr|B4SF16|B4SF16_PELPB	TYVCPGGKLTIGYGHT
tr|B6SD17|B6SD17_9VIRU	AYQCSAGRWTIGYGHT
tr|B6SCY8|B6SCY8_9VIRU	AYQCRAGRWTLGYGHT
tr|Q2NTT9|Q2NTT9_SODGM	AYQCSADRWTIGYGHT
tr|Q2NTN7|Q2NTN7_SODGM	AYQCSADRWTIGYGHT
tr|Q6HAD9|Q6HAD9_9ENTR	AYQCSANVWSIGYGHT
tr|B6VNM2|B6VNM2_PHOAA	AYQCSANVWTIGYGHT
tr|Q2A096|Q2A096_9VIRU	AYQCRAGIWTIGYGHT
tr|Q4LBS9|Q4LBS9_SODGL	AYQCRAGIWTIGYGHT
tr|B9MGZ0|B9MGZ0_DIAST	PYICPAGYWTIGYGHL
tr|C6NTT6|C6NTT6_9GAMM	PYVCPAGYWTIGYGHL
tr|A6T1M1|A6T1M1_JANMA	PYVCPAGFWTIGYGHL
tr|C6XGM6|C6XGM6_LIBAP	RDIG-GGAWTIGYGHT
tr|D1Q094|D1Q094_9BACT	RDSG--GKPTIGYGHT
tr|C3X1U7|C3X1U7_OXAFO	AYQDSKGLWTIGYGHT
tr|C4U1T3|C4U1T3_YERKR	PYQCSAGVWTSGIGHT
tr|C4SNR0|C4SNR0_YERFR	PYQCSAGVWTSGIGHT
tr|C9XYT3|C9XYT3_CROTZ	PYQCSAGVWTSGIGHT
tr|B7NIL7|B7NIL7_ECO7I	PYQCSAGVWTDGIGNT
tr|Q8FJH5|Q8FJH5_ECOL6	PYQCSAGVWTDGIGNT
tr|B7UMS0|B7UMS0_ECO27	PYQCSAGVWTDGIGNT
tr|B7MYT1|B7MYT1_ECO81	PYQCSAGVWTDGIGNT
tr|B5FS42|B5FS42_SALDC	PYQCSAGVWTDGIGNT
tr|A7ZYD1|A7ZYD1_ECOHS	PYQCSAGVWTDGIGNT
tr|Q8Z351|Q8Z351_SALTI	PYQCSAGVWTDGIGNT
tr|B7MJI0|B7MJI0_ECO45	PYQCSAGVWTDGIGNT
tr|B1IWU6|B1IWU6_ECOLC	PYQCSAGVWTDGIGNT
tr|C8QCC9|C8QCC9_9ENTR	PYQCDAGKWTDGIGNT
tr|Q4FSX3|Q4FSX3_PSYA2	AYQDTGKVWTIGYGHT
tr|D0BX92|D0BX92_9GAMM	AYQDTGGVWTIGYGTI
tr|A9INC3|A9INC3_BART1	AYKDAIGVWTIGYGHT
tr|Q6G4P4|Q6G4P4_BARHE	AYKDAIGVWTIGYGHT
tr|A9IPJ6|A9IPJ6_BART1	AYKDAIGVWTIGYGHT
tr|C6AD90|C6AD90_BARGA	AYKDAIGVWTIGYGHT
tr|C6ABV9|C6ABV9_BARGA	AYKDAIGVWTIGYGHT
tr|A9IY00|A9IY00_BART1	AYKDAIGVWTIGYGHT
tr|C6AAE8|C6AAE8_BARGA	AYKDAIGVWTIGYGHT
tr|Q6G209|Q6G209_BARHE	AYQDSIGVWTIGYGHT
tr|Q6FYR6|Q6FYR6_BARQU	AYQDAVGLWTIGYGHT
tr|C6AD78|C6AD78_BARGA	AYRDTACVWTIGYGHT
tr|C6ABV0|C6ABV0_BARGA	AYRDTACVWTIGYGHT
tr|A9ITY9|A9ITY9_BART1	AYRDTACIWTIGYGHT
tr|A9IY31|A9IY31_BART1	AYRDTACIWTIGYGHT
tr|Q6G365|Q6G365_BARHE	AYRDASGVWTIGYGHT
tr|C6ADN3|C6ADN3_BARGA	AYQDTSGVWTIGYGHT
tr|A9IN99|A9IN99_BART1	AYEDSGGIWTIGYGHT
tr|C6ABT0|C6ABT0_BARGA	AYEYSGGVWTIGYGHT
tr|A1URH0|A1URH0_BARBK	AYQDAVGIWTIGHGHT

tr|Q7P041|Q7P041_CHRVO AYQDMVGVWTIGYGHT
tr|Q8PNJ6|Q8PNJ6_XANAC SYVCPAGKLTIGYGHT
tr|C8CLH9|C8CLH9_9CAUD PYKDIVGVWTVCYGHT
tr|D2U1M6|D2U1M6_9ENTR PYKDGGGVLTVCYGHT
tr|Q1RHJ8|Q1RHJ8_RICBR PYYCPAGLKTIGYGHV
tr|A8GV89|A8GV89_RICB8 PYYCPAGLKTIGYGHV
tr|Q2N9G4|Q2N9G4_ERYLH VYRDVAGYPTVGVGHL
tr|A3WB87|A3WB87_9SPHN VYRDVAGYPTVGVGHL
tr|A5P716|A5P716_9SPHN VYRDVAGYPTVGVGHL
tr|C3L4H1|C3L4H1_AMOA5 VYKDVAGIETIGYGHV
tr|C5AG15|C5AG15_BURGB YYNDSKGYCTVGWGHL
tr|B2JV34|B2JV34_BURP8 YYDDSKGYCTVGWGHL
tr|A9MN07|A9MN07_SALAR AYNDSEGYCTIGYGHL
tr|Q8ZLC6|Q8ZLC6_SALTY KYRDRQGNWVIGYGHM
tr|Q5PJR8|Q5PJR8_SALPA KYRDRQGNWVIGYGHM
tr|D0ZKG2|D0ZKG2_SALT1 KYRDRQGNWVIGYGHM
tr|B5RGS1|B5RGS1_SALG2 KYRDRQGNWVIGYGHM
tr|B5R406|B5R406_SALEP KYRDRQGNWVIGYGHM
tr|B5FKM6|B5FKM6_SALDC KYRDRQGNWVIGYGHM
tr|B5BHQ0|B5BHQ0_SALPK KYRDRQGNWVIGYGHM
tr|B4T8E1|B4T8E1_SALHS KYRDRQGNWVIGYGHM
tr|B4SWF3|B4SWF3_SALNS KYRDRQGNWVIGYGHM
tr|C9X727|C9X727_SALTD KYRDRQGNWVIGYGHM
tr|B5P1B9|B5P1B9_SALET KYRDRQGNWVIGYGHM
tr|B5MX04|B5MX04_SALET KYRDRQGNWVIGYGHM
tr|B5C3G2|B5C3G2_SALET KYRDRQGNWVIGYGHM
tr|Q57IL7|Q57IL7_SALCH KYRDRQGNWVIGYGHM
tr|Q8Z279|Q8Z279_SALTI KYRDRQGNWVIGYGHM
tr|C0Q164|C0Q164_SALPC KYRDRQGNWVIGYGHM
tr|B4TZ04|B4TZ04_SALSV KYRDRQGNWVIGYGHM
tr|B5Q7E2|B5Q7E2_SALVI KYRDRQGNWVIGYGHM
tr|B5PC54|B5PC54_SALET KYRDRQGNWVIGYGHM
tr|B5C9I7|B5C9I7_SALET KYRDRQGNWVIGYGHM
tr|B5F9H2|B5F9H2_SALA4 KYRDRQGNWVIGYGHM
tr|A9MUN0|A9MUN0_SALPB KYRDRQGNWVIGYGHM
tr|B5PRV6|B5PRV6_SALHA KYRDRQGNWVIGYGHM
tr|B5NSD2|B5NSD2_SALET KYRDRQGNWVIGYGHM
tr|B5ND52|B5ND52_SALET KYRDRQGNWVIGYGHM
tr|B4A5H0|B4A5H0_SALNE KYRDRQGNWVIGYGHM
tr|B3YCV0|B3YCV0_SALET KYRDRQGNWVIGYGHM
tr|B5MHG3|B5MHG3_SALET KYRDRQGNWVIGYGHM
