# Supplementary material for: Structural Relationships in the Lysozyme Superfamily: Significant Evidence for Glycoside Hydrolase Signature Motifs
Source: PLoS One. 2010 Nov 9;5(11):e15388. doi: 10.1371/journal.pone.0015388 (PMC2976769; doi:10.1371/journal.pone.0015388)
Supplement: Figure S6 — Multiple alignment of GH24l motif sequences. (PDF) [file pone.0015388.s007.pdf]

Figure S6. Multiple alignment of GH24l motif sequences.

```
                              ....|....
tr|O30554|O30554_NEIGO  SDASGRYQF
tr|D1DW23|D1DW23_NEIGO  SDASGRYQF
tr|D1DJA2|D1DJA2_NEIGO  SDASGRYQF
tr|D1DCT5|D1DCT5_NEIGO  SDASGRYQF
tr|Q5K692|Q5K692_NEIGO  SDASGRYQF
tr|D1E2S1|D1E2S1_NEIGO  SDASGRYQF
tr|D1EFJ4|D1EFJ4_NEIGO  SDASGRYQF
tr|B9TBF9|B9TBF9_RICCO  TTAAGMYQI
tr|D1TAW4|D1TAW4_9BURK  TTAAGLYQI
tr|A2SGW7|A2SGW7_METPP  SSAAGRYQL
tr|D1UM48|D1UM48_9BURK  STAAGAYQF
tr|B2KJU8|B2KJU8_BURPS  SSAAGAYQF
tr|Q0K1M1|Q0K1M1_RALEH  SSAAGAYQF
tr|A7FIU8|A7FIU8_YERP3  TSAAGRYQF
tr|C4SUV2|C4SUV2_YERFR  TSAAGRYQF
tr|D2C286|D2C286_DICD5  TSAAGRYQF
tr|C5A0Z7|C5A0Z7_ECOBW  STGAGRYQL
tr|C6ZCX1|C6ZCX1_LAMBD  STGAGRYQL
tr|C5W1U5|C5W1U5_ECOBB  STGAGRYQL
tr|C6EJG0|C6EJG0_ECOBD  STGAGRYQL
tr|Q716B5|Q716B5_BPSFV  STAAGRYQL
tr|B7UG35|B7UG35_ECO27  STAAGRYQL
tr|Q0T7S1|Q0T7S1_SHIF8  STAAGRYQL
tr|B3H979|B3H979_ECOLX  STAAGRYQL
tr|C8UCS0|C8UCS0_ECO1A  STAAGRYQL
tr|A5VW81|A5VW81_9CAUD  STAAGRYQL
tr|A1ADL8|A1ADL8_ECOK1  STAAGRYQL
tr|Q1R938|Q1R938_ECOUT  STAAGRYQL
tr|B7MGZ0|B7MGZ0_ECO45  STAAGRYQL
tr|Q9MCT2|Q9MCT2_BPHK0  STAAGRYQL
tr|D3GYY0|D3GYY0_ECOLX  STAAGRYQL
tr|C6ZR65|C6ZR65_9CAUD  STAAGRYQL
tr|B9UDJ1|B9UDJ1_9CAUD  STAAGRYQL
tr|D2A7X6|D2A7X6_SHIF2  STAAGRYQL
tr|C8TV15|C8TV15_ECO26  STAAGRYQL
tr|C1HNI2|C1HNI2_9ESCH  STAAGRYQL
tr|Q9MCN4|Q9MCN4_BPHK7  STAAGRYQL
tr|Q77IT9|Q77IT9_BPHK6  STAAGRYQL
tr|Q57SQ6|Q57SQ6_SALCH  STAAGRYQL
tr|C0PZH3|C0PZH3_SALPC  STAAGRYQL
tr|A7ZWN9|A7ZWN9_ECOHS  STAAGRYQL
tr|B3BQ98|B3BQ98_ECO57  STAAGRYQL
tr|B3HV98|B3HV98_ECOLX  STAAGRYQL
tr|B3WIU8|B3WIU8_ECOLX  STAAGRYQL
tr|B3A9F3|B3A9F3_ECO57  STAAGRYQL
tr|B3AU10|B3AU10_ECO57  STAAGRYQL
tr|B2PPB9|B2PPB9_ECO57  STAAGRYQL
tr|B2NUN6|B2NUN6_ECO57  STAAGRYQL
tr|B2P6W5|B2P6W5_ECO57  STAAGRYQL
tr|B3B464|B3B464_ECO57  STAAGRYQL
tr|B3BIH7|B3BIH7_ECO57  STAAGRYQL
tr|B3BZ35|B3BZ35_ECO57  STAAGRYQL
tr|B6ZZG5|B6ZZG5_ECO57  STAAGRYQL
tr|B5YXF1|B5YXF1_ECO5E  STAAGRYQL
tr|C6URV2|C6URV2_ECO5T  STAAGRYQL
tr|Q8X705|Q8X705_ECO57  STAAGRYQL
tr|C1HJG8|C1HJG8_9ESCH  STAAGRYQL
tr|B7MJP3|B7MJP3_ECO45  STAAGRYQL
tr|A1AEH9|A1AEH9_ECOK1  STAAGRYQL
tr|Q1R868|Q1R868_ECOUT  STAAGRYQL
tr|C8U911|C8U911_ECO10  STAAGRYQL
tr|B7M8F2|B7M8F2_ECO8A  STAAGRYQL
tr|B3HTJ2|B3HTJ2_ECOLX  STAAGRYQL
tr|B2N2G4|B2N2G4_ECOLX  STAAGRYQL
tr|B3WTB5|B3WTB5_ECOLX  STAAGRYQL
tr|Q8Z617|Q8Z617_SALTI  STAAGRYQL
tr|B7NA40|B7NA40_ECOLU  STAAGRYQL
tr|Q8SBE0|Q8SBE0_BPSF5  STAAGRYQL
tr|B5FL63|B5FL63_SALDC  STAAGRYQL
tr|B7LJL2|B7LJL2_ESCF3  STAAGRYQL
tr|B3I771|B3I771_ECOLX  STAAGRYQL
tr|B2U3U2|B2U3U2_SHIB3  STAAGRYQL
tr|B5XR49|B5XR49_KLEP3  STAAGRYQL
```

```
tr|B1GS82|B1GS82_9CAUD  STAAGRYQV
tr|B3RGM4|B3RGM4_9CAUD  SSAAGRYQV
tr|B2FRB3|B2FRB3_STRMK  SSAAGRYQF
tr|B4SMC4|B4SMC4_STRM5  SSAAGRYQF
tr|B8KZT2|B8KZT2_9GAMM  SSAAGRYQF
tr|Q88G71|Q88G71_PSEPK  SSAAGRYQF
tr|Q0BEL5|Q0BEL5_BURCM  STAAGRYQI
tr|Q6J1Q5|Q6J1Q5_9CAUD  STAAGRYQI
tr|A4LDY2|A4LDY2_BURPS  STAAGRYQI
tr|A4JDH1|A4JDH1_BURVG  STAAGRYQL
tr|Q9MC90|Q9MC90_BPD3   STAAGRYQL
tr|B5S318|B5S318_RALSO  STAAGRYQL
tr|Q8XYQ8|Q8XYQ8_RALSO  STAAGRYQL
tr|B4SNI7|B4SNI7_STRM5  STGAGRYQL
tr|B5PLH9|B5PLH9_SALET  STASGRYQQ
tr|C9XHB2|C9XHB2_SALTD  STASGRYQQ
tr|B5MYZ0|B5MYZ0_SALET  STASGRYQQ
tr|Q6K1I1|Q6K1I1_9CAUD  STASGRYQQ
tr|B5MU90|B5MU90_SALET  STASGRYQQ
tr|O80309|O80309_BP186  STASGRYQQ
tr|C6V0R3|C6V0R3_ECO5T  STASGRYQQ
tr|B7MEC0|B7MEC0_ECO45  STASGRYQQ
tr|B5YUG0|B5YUG0_ECO5E  STASGRYQQ
tr|A1A9F8|A1A9F8_ECOK1  STASGRYQQ
tr|C1NGQ4|C1NGQ4_9ESCH  STASGRYQQ
tr|B3BVK4|B3BVK4_ECO57  STASGRYQQ
tr|B3AVQ9|B3AVQ9_ECO57  STASGRYQQ
tr|B3A901|B3A901_ECO57  STASGRYQQ
tr|Q76ZD6|Q76ZD6_9CAUD  STASGRYQQ
tr|B1EPP6|B1EPP6_9ESCH  STASGRYQQ
tr|B2N3V9|B2N3V9_ECOLX  STASGRYQQ
tr|B2PP35|B2PP35_ECO57  STASGRYQQ
tr|B2PD69|B2PD69_ECO57  STASGRYQQ
tr|B2NUI4|B2NUI4_ECO57  STASGRYQQ
tr|Q7Y4E4|Q7Y4E4_BPP2   STASGRYQQ
tr|B3BLU1|B3BLU1_ECO57  STASGRYQQ
tr|B1LMX9|B1LMX9_ECOSM  STASGRYQQ
tr|A6TE68|A6TE68_KLEP7  STASGRYQQ
tr|C6CF31|C6CF31_DICZE  STAAGRYQQ
tr|D2BWX0|D2BWX0_DICD5  STAAGRYQQ
tr|C6C704|C6C704_DICDC  STAAGRYQQ
tr|B8L8I5|B8L8I5_9GAMM  SNASGRYQF
tr|B8L9S2|B8L9S2_9GAMM  SNASGRYQF
tr|Q46YK5|Q46YK5_RALEJ  SNAAGRYQH
tr|Q6UYK3|Q6UYK3_9CAUD  STASGRYQQ
tr|A4SL92|A4SL92_AERS4  STAAGRYQF
tr|A5X9I5|A5X9I5_9CAUD  STAAGRYQF
tr|B0JGM5|B0JGM5_MICAN  STAAGRYQF
tr|C4U050|C4U050_YERKR  SSAAGAYQV
tr|B4E744|B4E744_BURCJ  SSAAGAYQI
tr|B8HW77|B8HW77_CYAP4  TTAAGRYQF
tr|Q8DK42|Q8DK42_THEEB  TTAAGRYQF
tr|B0CAN4|B0CAN4_ACAM1  TTAAGRYQF
tr|B7KAY2|B7KAY2_CYAP7  TTAAGRYQF
tr|B4B7H5|B4B7H5_9CHRO  STAAGRYQF
tr|B4VYB5|B4VYB5_9CYAN  TTAAGRYQF
tr|Q3M520|Q3M520_ANAVT  STAAGRYQI
tr|Q8YTG5|Q8YTG5_ANASP  STAAGRYQI
tr|A0ZIR9|A0ZIR9_NODSP  STAAGRYQI
tr|B2J3F8|B2J3F8_NOSP7  STAAGRYQI
tr|P74105|P74105_SYNY3  STASGRYQF
tr|B5W3E0|B5W3E0_SPIMA  TTAAGRYQF
tr|Q117E2|Q117E2_TRIEI  STAAGRYQM
tr|B4WPE7|B4WPE7_9SYNE  TTAAGRYQF
tr|A3IRW1|A3IRW1_9CHRO  STAAGRYQF
tr|B1WYC8|B1WYC8_CYAA5  STAAGRYQF
tr|B7JUQ2|B7JUQ2_CYAP8  STAAGRYQF
tr|Q55936|Q55936_SYNY3  STAAGRYQF
tr|A0YIW4|A0YIW4_9CYAN  TTAAGRYQL
tr|A4CXH0|A4CXH0_SYNPV  SAAAGAYQF
tr|A5GJU0|A5GJU0_SYNPW  SAAAGAYQF
tr|A3Z536|A3Z536_9SYNE  SAAAGAYQF
tr|Q05R76|Q05R76_9SYNE  SAAAGAYQF
tr|Q0I904|Q0I904_SYNS3  SAAAGAYQF
tr|Q7V8D3|Q7V8D3_PROMM  SAAAGAYQF
tr|A2CAU6|A2CAU6_PROM3  SAAAGAYQF
tr|Q3AYC4|Q3AYC4_SYNS9  SAAAGAYQF
```

```
tr|Q065Z3|Q065Z3_9SYNE  SAAAGAYQF
tr|D0CI57|D0CI57_9SYNE  SAAAGAYQF
tr|Q3AKT8|Q3AKT8_SYNSC  SAAAGAYQF
tr|Q7U664|Q7U664_SYNPX  SAAAGAYQF
tr|Q3AY16|Q3AY16_SYNS9  SAAAGAYQF
tr|Q067I0|Q067I0_9SYNE  SAAAGAYQF
tr|D0CJC1|D0CJC1_9SYNE  SAAAGAYQF
tr|Q3AJM5|Q3AJM5_SYNSC  SAAAGAYQF
tr|Q7U6M5|Q7U6M5_SYNPX  SAAAGAYQF
tr|Q7V7S4|Q7V7S4_PROMM  SAAAGAYQF
tr|A2C9Z4|A2C9Z4_PROM3  SAAAGAYQF
tr|A5GL12|A5GL12_SYNPW  SAAAGAYQF
tr|A4CUP6|A4CUP6_SYNPV  SAAAGAYQF
tr|Q0IA84|Q0IA84_SYNS3  SAAAGAYQF
tr|A3Z6Z0|A3Z6Z0_9SYNE  SAAAGAYQF
tr|Q05T44|Q05T44_9SYNE  SAAAGAYQF
tr|A3Z1Y9|A3Z1Y9_9SYNE  SAAAGAYQF
tr|B5IJ50|B5IJ50_9CHRO  SAAAGAYQF
tr|A5GTW9|A5GTW9_SYNR3  SAAAGAYQF
tr|B5IL03|B5IL03_9CHRO  SAAAGAYQF
tr|A3Z1B0|A3Z1B0_9SYNE  SAAAGAYQM
tr|A5GTZ0|A5GTZ0_SYNR3  SAAAGAYQF
tr|Q8ZS41|Q8ZS41_ANASP  STAAGAYQM
tr|Q3M149|Q3M149_ANAVT  STAAGAYQM
tr|Q10XQ1|Q10XQ1_TRIEI  SDAAGRYQF
```
