# Supplementary material for: Structural Relationships in the Lysozyme Superfamily: Significant Evidence for Glycoside Hydrolase Signature Motifs
Source: PLoS One. 2010 Nov 9;5(11):e15388. doi: 10.1371/journal.pone.0015388 (PMC2976769; doi:10.1371/journal.pone.0015388)
Supplement: Figure S7 — Multiple alignment of GH46 motif sequences. (PDF) [file pone.0015388.s008.pdf]

Figure S7. Multiple alignment of GH46 motif sequences.

```
                                     10
                               ....|....|....|..
tr|D1XP65|D1XP65_9ACTO  DGRGYTAGIIGFCSGTG
tr|C9NEY0|C9NEY0_9ACTO  DGRGYTAGIIGFCSGTG
tr|Q82LJ5|Q82LJ5_STRAW  DGRGYTAGIIGFCSGTG
tr|Q9RJ88|Q9RJ88_STRCO  DGRGYTAGIIGFCSGTG
tr|C9E0E7|C9E0E7_STRLI  DGRGYTAGIIGFCSGTG
tr|A0AD68|A0AD68_STRAM  DGRGYTAGIIGFCSGTG
tr|B4VD22|B4VD22_9ACTO  DQRGYTAGIIGFCSGTG
tr|B1Q2K4|B1Q2K4_9ACTO  DGRGYTGGIIGFCSGTH
tr|B5GIH8|B5GIH8_9ACTO  DGRGYTAGIIGFCSGTG
tr|D2AW85|D2AW85_STRRD  DGRGYTAGIIGFCSGTG
tr|B5GRN3|B5GRN3_STRCL  DGRGYTAGVAGFCSGTG
tr|B5HRQ8|B5HRQ8_9ACTO  DGRGYTAGIIGFCSGTS
tr|C6WIU3|C6WIU3_ACTMD  DGRGYTAGIIGFCSGTG
tr|Q9LBG4|Q9LBG4_9PSEU  DGRGYTAGIIGFCSGTG
tr|A9WUI6|A9WUI6_RENSM  DDRGYTGGIIGFTSGTG
tr|C2BY78|C2BY78_LISGR  DGRGYTAGIIGFCSGTG
tr|D3J1N7|D3J1N7_9BURK  MG-GYTGGIIGFTSGTS
tr|C9NEX9|C9NEX9_9ACTO  DGRGYTAGIIGFCSGTG
tr|D1XP66|D1XP66_9ACTO  DGRGYTAGVIGFCSGTG
tr|B5HFR3|B5HFR3_STRPR  DGRGYTAGIIGFCSGTG
tr|Q8KZM5|Q8KZM5_9PSED  DGRGYTGGLIGFTSGTG
tr|A7KBW5|A7KBW5_9MICO  DGRGYTGGLIGFTSGTG
tr|Q7WT07|Q7WT07_9ACTO  DGRGYTAGIIGFCSGTG
tr|D1XSH1|D1XSH1_9ACTO  DGNGYTAGIVGFCSGTN
tr|B5H4T0|B5H4T0_STRPR  DGNGYTAGIIGFCSGTN
tr|Q82A68|Q82A68_STRAW  DGRGYTAGVIGFCTGTH
tr|A7Z8H9|A7Z8H9_BACA2  DGRGYTCGRAGFTTATG
tr|A0EQW7|A0EQW7_9BACI  DGRGYTCGRAGFTTATG
tr|Q9ET84|Q9ET84_BACAM  DGRGYTCGRAGFTTATG
tr|B9W6G0|B9W6G0_BACSU  DGRGYTCGRAGFTTATG
tr|D0FHB6|D0FHB6_BACSU  DGRGYTCGRAGFTTATG
tr|Q0PVM7|Q0PVM7_BACAM  DGRGYTCGRAGFTTATG
tr|O24825|O24825_9BACL  DERGYSIGIFGATTGGP
tr|Q2PWA1|Q2PWA1_9BACL  DDRGYTIVLFGATTGGS
tr|Q7NR53|Q7NR53_CHRVO  DKRGYTIGIFGATTGGP
tr|Q9XDS6|Q9XDS6_BURGA  DDRGYTMGIFGATTGGP
tr|Q7M0I1|Q7M0I1_BACCO  DQPAYPFAIGGASADGG
tr|Q8VU68|Q8VU68_BACCO  GQPAYPFAIGGASADGG
tr|A7RBE5|A7RBE5_PBCVA  DGRGWTVTLYGACSGTG
tr|A7IWR8|A7IWR8_PBCVN  DGRGWTVTLYGACSGTG
tr|Q84608|Q84608_PBCV1  DGRGWTVTIYGACSGTG
tr|O12288|O12288_9PHYC  DGRGWTVTIYGACSGTG
tr|A7J6E1|A7J6E1_PBCVF  DGRGWTVTIYGACSGTG
tr|A7ITH1|A7ITH1_PBCVM  DGRGWTVTIYGACSGTG
tr|A7K8G4|A7K8G4_9PHYC  DGRGWTVTLYGACSGTG
tr|B2J4Y1|B2J4Y1_NOSP7  DGRGVTIGIYGATTGKG
```
